# Unraveling the Inconsistencies of Cardiac Differentiation Efficiency Induced by the GSK3β Inhibitor CHIR99021 in Human Pluripotent Stem Cells

**DOI:** 10.1016/j.stemcr.2018.03.023

**Published:** 2018-04-26

**Authors:** Filip Laco, Tsung Liang Woo, Qixing Zhong, Radoslaw Szmyd, Sherwin Ting, Fahima Jaleel Khan, Christina L.L. Chai, Shaul Reuveny, Allen Chen, Steve Oh

**Affiliations:** 1 Bioprocessing Technology Institute, 20 Biopolis Way, Centros #06-01, Singapore 138668, Singapore; 2 Department of Pharmacy, Faculty of Science, National University of Singapore, 18 Science Drive 4, Singapore 117543, Singapore; 3 Institute of Molecular and Cell Biology, 61 Biopolis Drive, Proteos #03-01, Singapore 138673, Singapore

**Keywords:** CHIR99021, cell cycle, cardiomyocytes, differentiation, pluripotent stem cells, TCF7L1, β-catenin

## Abstract

Cardiac differentiation efficiency is hampered by inconsistencies and low reproducibility. We analyzed the differentiation process of multiple human pluripotent stem cell (hPSC) lines in response to dynamic GSK3β inhibition under varying cell culture conditions. hPSCs showed strong differences in cell-cycle profiles with varying culture confluency. hPSCs with a higher percentage of cells in the G1 phase of the cell cycle exhibited cell death and required lower doses of GSK3β inhibitors to induce cardiac differentiation. GSK3β inhibition initiated cell-cycle progression via cyclin D1 and modulated both Wnt signaling and the transcription factor (TCF) levels, resulting in accelerated or delayed mesoderm differentiation. The TCF levels were key regulators during hPSC differentiation with CHIR99021. Our results explain how differences in hPSC lines and culture conditions impact cell death and cardiac differentiation. By analyzing the cell cycle, we were able to select for highly cardiogenic hPSC lines and increase the experimental reproducibility by predicting differentiation outcomes.

## Highlights

• Lineage variety and cell culture density affect the cell cycle in hPSCs
• CHIR99021 is cytotoxic to hPSCs with reduced S/G2/M cell-cycle phases
• Cardiac differentiation reproducibility depends on cell-cycle consistency in hPSCs
• Cell cycle and TCF protein levels modulate CHIR99021-induced differentiation

## Teaser

In this article, Laco and colleagues show that human pluripotent stem cells are characterized by individual cell-cycle profiles. Changes in cell-cycle profiles, mainly due to cell culture density, affect Wnt pathway-induced cardiac differentiation and cell death when GSK3β inhibitors are applied. We show that cell-cycle validation is crucial for stem cell quality and experimental reproducibility.

## Introduction

Glycogen synthase kinase-3β (GSK3β) has multiple cellular substrates, and they play strategic roles in various essential physiological processes, such as development, the cell cycle, and apoptosis. The main focus of GSK3β in stem cells is associated with its role as a signal transduction factor of the canonical Wnt/β-catenin pathway through the modulation of the GSK3β/β-catenin protein complex via Wnt ligands. GSK3β phosphorylates β-catenin, among other proteins (e.g., cyclin D1), leading to their degradation. The absence of Wnt ligands or the inhibition of GSK3β by growth factors (e.g., fibroblast growth factor 2) and small molecules (e.g., CHIR99021) suppresses substrate phosphorylation by inactivating GSK3β (McCubrey et al., 2014). The canonical Wnt/β-catenin signaling pathway has been suggested to regulate the self-renewal of human pluripotent stem cells (hPSCs) (Sato et al., 2004). Inactivated GSK3β allows the accumulation of β-catenin in the cellular cytosol, which transfers to the nucleus. Nuclear β-catenin forms a complex with transcription factor (TCF) proteins to activate the Wnt pathway gene targets (McCubrey et al., 2014). These Wnt gene targets affect the expression of pluripotency and developmental factors associated with the primitive streak and the germ layers (Hodar et al., 2010). Short-term Wnt induction maintains pluripotency, whereas long-term induction via GSK3β inhibition induces stem cell differentiation to endo- and mesoderm derivatives (Huang et al., 2015) and can further solely regulate the developmental division of the mesoderm into the paraxial and lateral mesoderm, which gives rise to the cardiac lineage (Tan et al., 2013). Efficient cardiac differentiation has been demonstrated with GSK3β inhibition via the small-molecule inhibitor CHIR99021 (CHIR) (Lian et al., 2012). However, the reproducibility of the protocol requires cell line- and cell culture-dependent optimization and can easily lead to heterogeneous differentiation results (Sepac et al., 2012). Moreover, it is not clear how a single transient induction with a GSK3β inhibitor is able to direct highly efficient lineage specification toward cardiomyocytes. Therefore, we studied the effect of CHIR induction in hPSC lines to understand its dynamics and facilitate mesoderm formation resulting in cardiac differentiation.

CHIR is a kinase inhibitor of GSK3α and GSK3β, with off-target effects on kinases within the CDK2-cyclin A2/E cell-cycle complex (An et al., 2014). Moreover, GSKα/β regulates the cell cycle via the mediation of cyclin D1/E (McCubrey et al., 2014) and the chromatin alignment of mitotic cells (Tighe et al., 2007, Yoshino and Ishioka, 2015). GSK inhibitors, such as AR-A014418, CHIR99021, CHIR98014, BIO, and SB-216763, have been reported to induce dose-dependent cell apoptosis in cancer and mouse embryonic stem cells (Naujok et al., 2014, Yoshino and Ishioka, 2015). hPSC differentiation with GSK3β inhibitors often underreports aspects of cell death, which are an essential part of developmental processes and applied bioprocess technologies. Therefore, in this study, we examined the effect of CHIR not only on hPSC line differentiation but also on cytotoxicity, cell growth, and the cell cycle.

We demonstrated that CHIR affected the cell cycle and differentiation simultaneously during the initial phase of differentiation. Changes in cell culture (e.g., cell culture density) affect the cell cycle and the dose dependency of CHIR to induce cardiac differentiation. The denser the cell cultures and the lower the S and G2 cell-cycle phases of hPSCs, the stronger was the cytotoxic effect of CHIR induction and the lower were the required doses of this inhibitor to induce cardiac differentiation, which led to decreased cardiac differentiation efficiency. Moreover, CHIR-induced mesoderm and cardiac differentiation by TCF level modulation and cell-cycle cyclin expression. Increased CHIR concentrations accelerated mesoderm development but required well-timed Wnt inhibition via TCF regulation to direct differentiation toward cardiomyocytes. We conclude that the differentiation process with GSK3β inhibition of hPSC lines should account for the initial compound cytotoxicity, the cell density, the cell-cycle state, the dose of CHIR, and the timing of TCF-regulated Wnt inhibition.

## Results

### GSK Inhibitor CHIR Regulates EB Formation and Cytotoxicity in hPSCs Depending on the Cell Cycle and the Cell Culture Density

In this section, the effect of culture confluency levels, namely, low (<50%), mid (70%), and high (>90%), on embryoid body (EB) formation and monolayer growth in the presence of CHIR was evaluated. We theorize that cell culture induces changes in the cell cycle, changing the cellular response to CHIR. We measured cell death and cell growth in terms of EB size, metabolism, and cell numbers, and related our findings to the cell-cycle phases of hPSCs.

**Figure 1:**
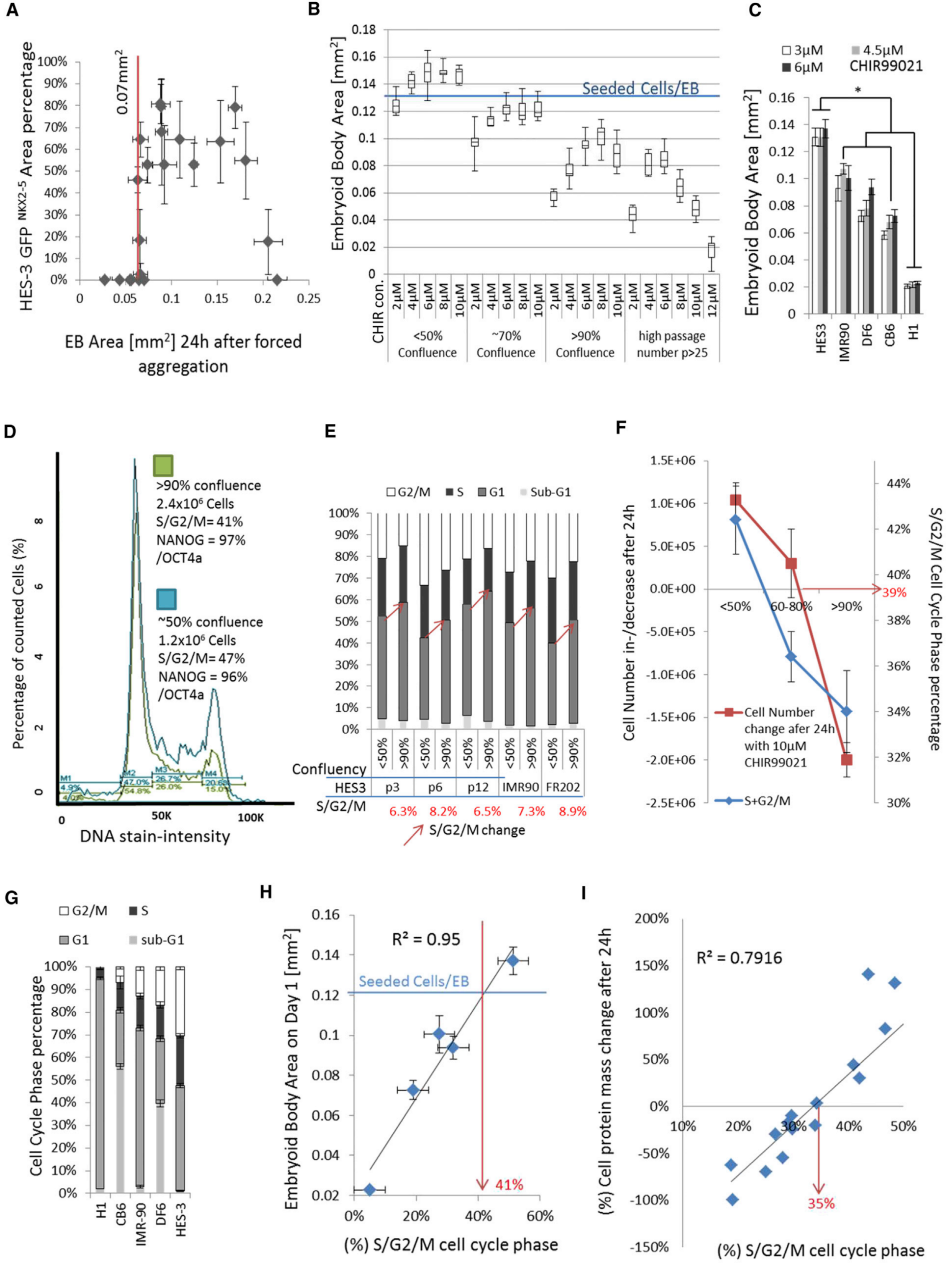
Analysis of the Interaction between Culture Conditions and Cell Cycle in Regard to CHIR Cytotoxicity (A) Correlation between initial EB area size on day 1 and percentage of the GFP area obtained after 10 days of differentiation (induction for 24 hr with 6 μM CHIR) (n = 4). (B) EB area size of 1-day-old forced aggregated EBs (12,500 cells/EB) generated from passage 10 (50%, 70%, and 90% culture confluency) and passage 25 (80% culture confluency) cell cultures (n = 16). (C) EB area size of 5 cell lines after 24 hr CHIR treatment (n = 10, ^∗^p ≤ 0.05). (D) Cell cycle, pluripotency, and cell count measurements of HES3 cells cultured at 50% and 90% culture density. (E) Cell-cycle profiles of IMR90, FR202, and HES3 passages 3, 6, and 12 cultured at 50% and 90% culture densities; average change of S/G2/M cell-cycle profile in hPSCs between 50% and 90% cell culture confluency was 7.4% ± 1.1% (n = 5). (F) Cell number change after treatment with CHIR and S/G2/M cell-cycle percentage differences of HES3 cells cultured at high (>90%), mid (60%–80%), and low (<50%) culture densities (n = 3). (G–I) Cell-cycle profiles of 5 hPSC lines (G) and their correlation between the percentage of S/G2/M cell-cycle phase (n = 3) and the measured EB area on day 1 after 24 hr CHIR induction (n = 8) (H). Correlation between percentage of S/G2/M cell-cycle phase of 17 hPSC lines and cell culture passages in monolayer cell culture and their change in cell protein mass (%) after 24 hr CHIR induction (I).

EB differentiation with the optimized EB standard protocol (Supplemental Experimental Procedures) induced a 50%–90% GFP/NKX2-5-positive EB area (Figure 1A). Interestingly, we observed in repeated HES3 differentiation experiments (n = 22, >160 EBs) using initial cell culture conditions, such as 60%–80% cell confluency, <10 cell culture passages, >85% NANOG and OCT4a cell population expression by flow cytometry, and a constant number of seeding cells (15,000 cells/EB), that a wide range of initial EB sizes were formed (0.025–0.225 mm^2^ or 2,500–22,500 cells/EB) on day 1. In addition, 22% of the experiments failed to induce GFP expression. Only HES3 EB sizes between 0.07 mm^2^ (>7,000 cells/EB) and 0.18 mm^2^ (<18,000 cells/EB) were able to express NKX2-5 (Figure 1A). Thus, we analyzed differences in cell culture conditions. The EB size decreased with increased culture density (Figure 1B). The EB size decreased when the CHIR concentration was <6 or >10 μM and in highly confluent cultures that were passaged repeatedly (Figure 1B). Cell loss was also induced in monolayer cultures with a 10 μM CHIR concentration with increasing cell density (Figure S1A). Individual cell lines showed different EB formation sizes after CHIR induction (Figure 1C). In addition, in monolayer cultures, we observed a similar cell line-dependent total protein loss or gain when the cells were cultured with 4–12 μM CHIR (Figure S1B, Table S2). The data indicate a cytotoxic effect of CHIR depending on the initial cell confluency and the cell line. We analyzed the pluripotency, mitotic activity, metabolites, and cell cycle of hPSC cells treated with CHIR to characterize differences between culture conditions and cell lines. Cell density did not affect the pluripotency population percentage of NANOG and OCT4a in HES3 cells (Figure 1D). The cell metabolism (MTT) of high-density cultures was reduced and declined upon differentiation induction (Figure S1C). The consumption of glucose and glutamine and the lactate production per cell were higher in less (<70%) confluent cell cultures, and a more acidified medium (pH 6.88) was observed in highly confluent cultures (>80%) (Figure S1A). Cell-cycle analyses of S/G2/M showed a difference of 7% (±1%) between high (>90%) and low (<50%) cell culture density across passages and cell lines (Figures 1D and 1E). Furthermore, we showed that an increase in cell density led to a decrease in the S/G/2M cell-cycle phases and a gradual loss of cell number after 24 hr with CHIR induction (Figures 1F and S1A). The cell cycle was shown to vary among cell lines, clones, and passage numbers (Figures 1E, 1G, and S1D; Table S2). We correlated the S/G2/M cell-cycle phases of five cell lines with the resulting EB formation area after 24 hr. In addition, we repeated that experiment in monolayer cultures, measuring protein mass changes in correlation with the S/G2/M or G2/M cell-cycle percentage (Figures 1G and 1H; Table S2). The data showed clear correlations between the cell cycle and both EB area and cell protein mass changes.

Our results demonstrate that CHIR was essential for EB formation but also induced cell death and growth in a dose-dependent manner in both EB and monolayer cultures. hPSC lines, clones, and passages showed significant differences in their S/G2/M cell-cycle profiles. Highly confluent cultures showed reduced metabolic activity and had a 7% lower S/G2/M cell-cycle phase when compared with 70%–50% confluent cell cultures. The difference in metabolism translated to cell growth or cell death after CHIR induction, resulting in over 50% cell mass difference between high and low cell culture confluence. CHIR was significantly cytotoxic in culture conditions and cell lines with a low (<35%–41%) S/G2/M cell-cycle phase. Conclusively, a high S/G2/M cell-cycle phase of >42% increased the cell mass by approximately 8% per S/G2/M cell-cycle profile percentage in hPSCs.

### CHIR Induces Initial Cell Death and Subsequent Cell Proliferation via Cell-Cycle Progression

In this section, we hypothesize that two separate effects of cell death and cell growth are induced upon CHIR induction in cells of a certain cell-cycle phase. Therefore, the fraction of apoptotic cells in a cell-cycle phase was identified, and the recovery of hPSCs post-CHIR treatment in terms of cell numbers, the cell cycle, and γ-tubulin and cyclin expression were examined.

**Figure 2:**
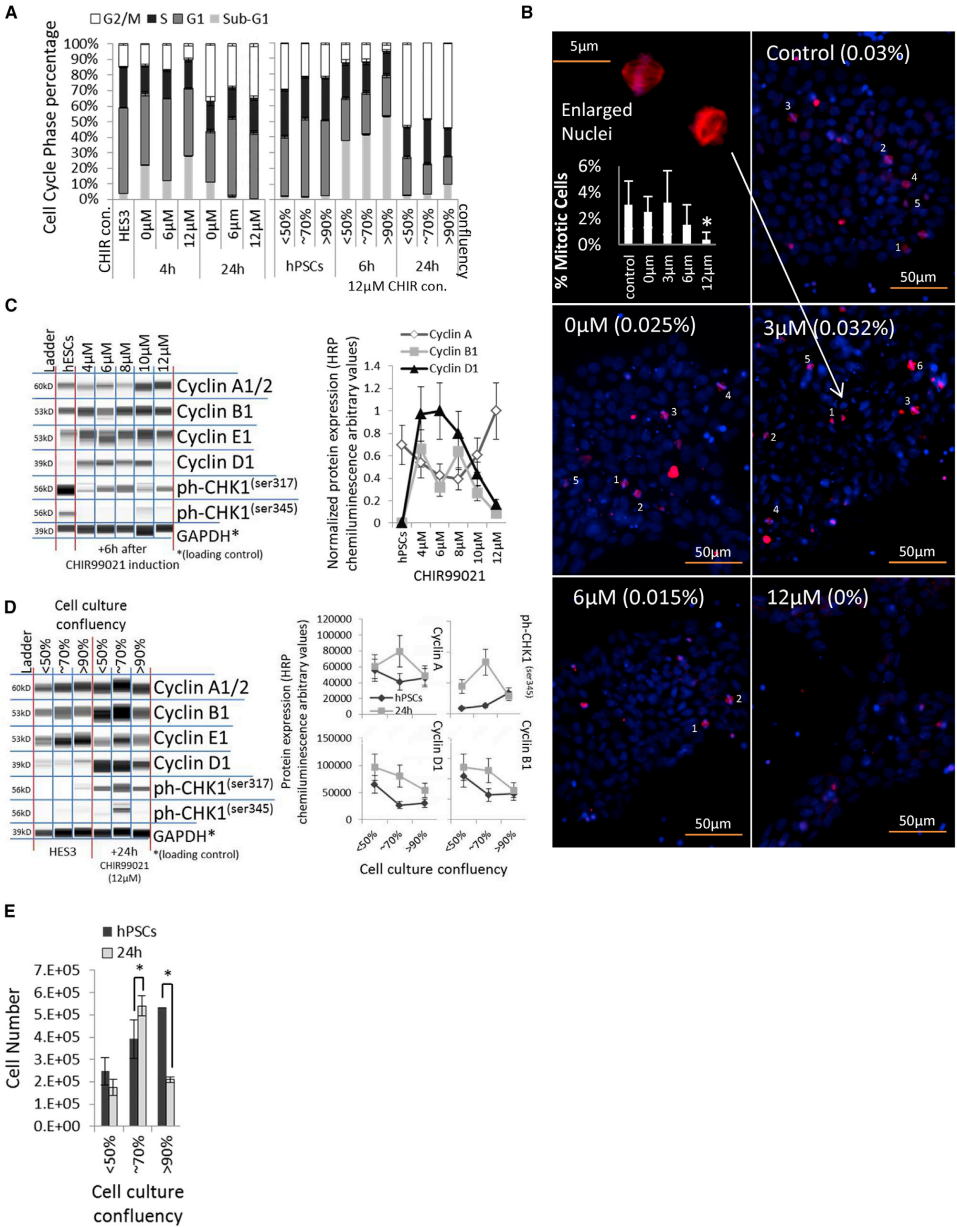
Analysis of hPSC Cell Cycle and Cyclins during CHIR Induction (A) Cell-cycle profile of HES3 cultures after induction with 0–12 μM CHIR, and cell-cycle profile of hPSCs cultured at 50%, 70%, and 90% culture confluency after 12 μM CHIR induction (n = 3). (B) Nuclei; DAPI (blue) and spindle apparatus of mitotic cells; γ-tubulin (red) staining of monolayer HES3 cultures treated for 8 hr with 0–12 μM CHIR. Numbers in brackets give the percentage of positively stained cells (n = 3 with 10 regions of interest each, ^∗^p ≤ 0.05). (C) Whole-cell protein blot expressions and quantitative analyses of HES3 cells after 4–12 μM CHIR induction. (D) Whole-cell protein blot expressions and quantitative analyses of HES3 cultures at 50%, 70%, and 90% culture confluency after 12 μM CHIR induction. (E) Cell number of hPSC cultures at 50%, 70%, and 90% culture confluency after 12 μM CHIR treatment (n = 3, ^∗^p ≤ 0.05).

Cell death of hPSCs occurred within 4–6 hr after differentiation initiation. The percentage of aberrant cells (sub-G1 percentage) increased, whereas the G1 and G2/M cell-cycle percentages decreased significantly in >90% confluent cell cultures and in cell cultures treated with both 0 and 12 μM CHIR (Figure 2A). The higher the CHIR concentration, the fewer proliferating, γ-tubulin-stained cells were quantified (Figure 2B). Moreover, the cell-cycle progression markers cyclin A and B1 were reduced in most cell culture conditions after 12 μM CHIR induction, indicating a loss of proliferating cells in the G2 cycle phase (Figure S2A). However, 24 hr after CHIR induction, the S/G2/M cell-cycle profiles were significantly increased in all conditions (Figure 2A), which indicates the hyperproliferation of the remaining non-apoptotic cell population. Cell-cycle progression is indicated by an increased S/G2/M cell-cycle profile in hPSCs and can be activated by cyclin D1 overexpression (Lim and Kaldis, 2013) as a result of GSK3β inhibition (McCubrey et al., 2014). Cyclin D1 expression levels responded to CHIR in a dose-dependent manner in HES3 EBs (Figure S2B). Induction with 4–8 μM CHIR induced cyclin D1 and B1 expression after 6 hr (Figure 2C). Cyclin D1, A1, and B1 remained expressed after 24 hr in cell culture, with <70% confluency in monolayer and EB cultures (Figures 2D and S2C), resulting in significant cell proliferation in comparison with high-density cell cultures (Figure 2E). We compared cyclin expression in four cell lines and found that cyclin D1 induction was induced in cell lines that showed an increased EB area after 24 hr of induction with CHIR (Figures S2D and S2E). Moreover, these cell lines expressed Troponin T after 14 days (Figure 2E). In addition, we confirmed cyclin D1 expression levels responded to CHIR in a dose-dependent manner in monolayer experiments with three cell lines (Figure S2F). Cyclin D1 was saturated at the peak Troponin T expression of each individual cell line, indicating a correlation between cyclin D1 expression and cardiac development (Figures S2F and S2G).

Interestingly, cell-cycle checkpoints and markers for DNA damage repair, such as CHK2, yH2A.X, p16, p21, GADDA45 (data not shown), and CHK1^ser345/317^ (Figures S2A and 2C), were not expressed or weakly expressed in hPSCs.

Our results demonstrate that cell death is transient upon cardiac differentiation with CHIR. Proliferative G2 cells diminished with increased cell confluency and CHIR doses. hPSCs did not express markers for DNA damage, indicating a loss of cell-cycle-driven DNA repair mechanisms (Desmarais et al., 2012, Desmarais et al., 2016). CHIR induced cyclin D1 expression. Hence, cell-cycle progression was induced, increasing cells in the S/G2/M cell-cycle phases and the expression of cyclin A and B1 after 24 hr. Cell growth was significantly increased with a culture confluency of <70%. However, differences in CHIR concentration and cell line dependency were noted.

### Cardiac Differentiation Efficiency of hPSCs with CHIR Is Regulated through the Cell Cycle, a Critical Factor for Differentiation Reproducibility and Cell Line Selection

We theorize in this section that the cell-cycle differences of hPSCs in response to CHIR affect cell fate decisions. Therefore, the cell-cycle and pluripotency changes in different culture conditions were examined and correlated with cardiac differentiation expression results.

**Figure 3:**
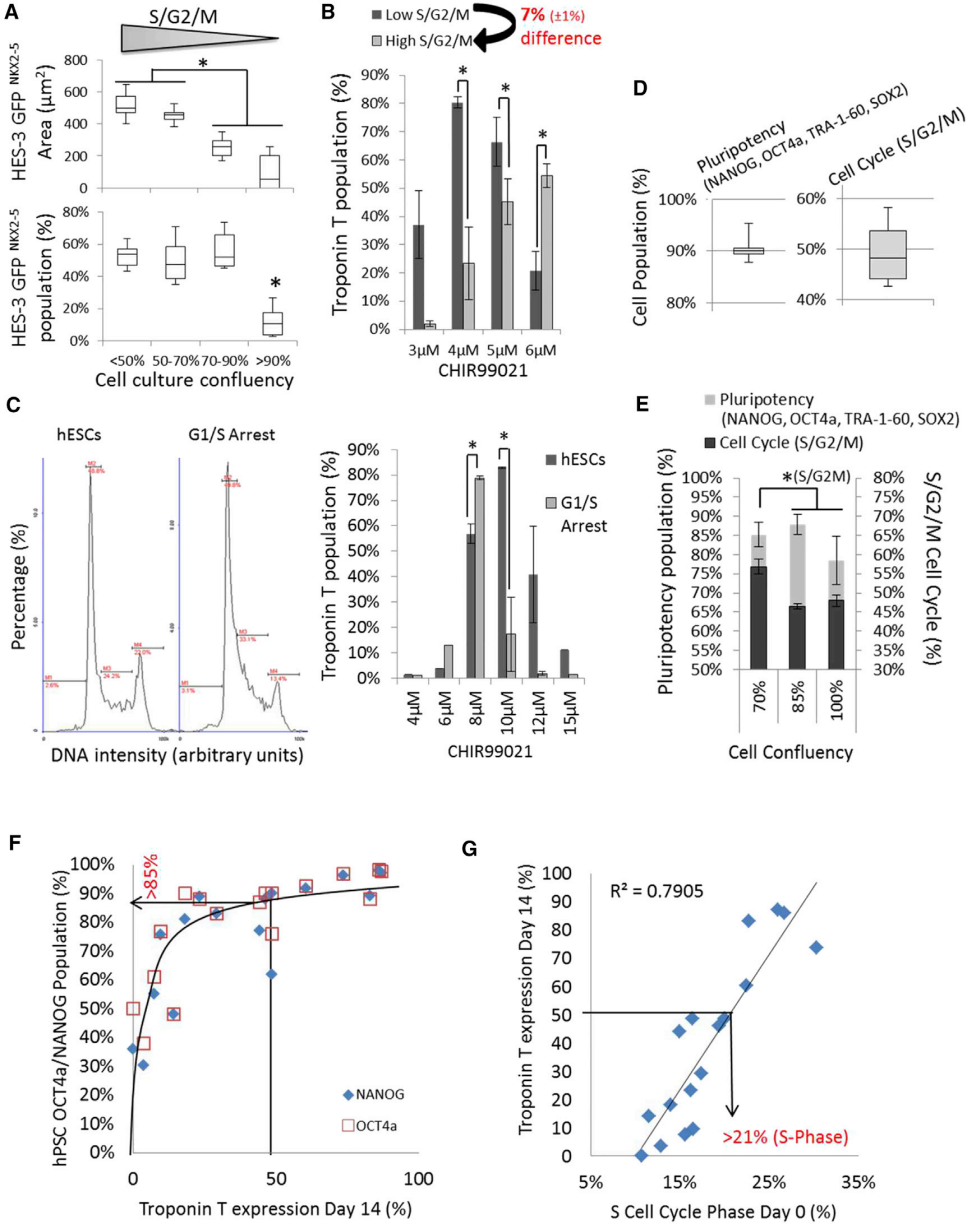
Analysis of hPSC Cell Cycles and Pluripotency in Relationship to Cardiac Differentiation with CHIR (A and B) Day 11 single-EB GFP/NKX2-5 expression area and percentage of differentiated (induction for 24 hr with 6 μM CHIR) HES3 cells cultured at <50%, 50%–70%, 70%–90%, and >90% culture confluency of low to high S/G2/M cell-cycle profiles (n = 8) (A). Day 11 Troponin T flow cytometry of EBs from differentiated IMR90 cell cultures with a 7% difference in S/G2/M cell-cycle profile (n = 4, ^∗^p ≤ 0.05) (B). (C) Cell-cycle profiles of HES3 cells and G1/S cell-cycle-arrested cells. Troponin T expression of differentiated HES3 with CHIR on day 14 (n = 3, ^∗^p ≤ 0.05). (D) Average pluripotency flow cytometer population of pluripotency markers and the percentage of positive population of the S/G2/M cell-cycle profile in hPSCs (n = 12). (E) Average pluripotency flow cytometer population of pluripotency markers and the percentage of positive population of the S/G2/M cell-cycle profile in hPSCs (n = 3, ^∗^p ≤ 0.05). (F) Asymptotic correlation of NANOG and OCT4a flow cytometry population (%) of 17 monolayer hPSC cell cultures on day 0 against peak Troponin T cardiomyocyte population on day 14 after 4–12 μM CHIR induction. (G) Correlation of peak Troponin T cardiomyocyte population on day 14 after 4–12 μM CHIR induction of 17 independent monolayer hPSC cultures against S cell-cycle phase (%) on day 0.

The NKX2-5-positive EB area decreased with increasing cell culture density (decreasing S/G2/M cell-cycle percentage) (Figure 3A), and the cardiac differentiation efficiency dropped significantly from 50% to 15% GFP/NKX2-5 expression at >90% culture confluency (Figure 3A). Similar results were observed in monolayer cultures with the IMR90 and FR202 cell lines, whereas HES3 cells were not affected at a 12 μM CHIR concentration (Figure S3A). However, a lower CHIR concentration was able to rescue the cardiac efficiency of high versus low (culture density) S/G2/M cell-cycle profile differences of 7% in IMR90 and HES3 EBs (Figures 3B and S3B). Alternatively, a timely induction with a Wnt inhibitor was able to partially rescue cardiac differentiation (Figure S3C). The results were confirmed by monolayer experiments with three additional cell lines (Figure S3D). Moreover, cell-cycle arrest of hPSCs at the G2 and G1/S transition-induced NKX2-5 expression at a lower CHIR concentration than observed under non-arrested culture conditions (Figure 3C). G1/S-arrested cells showed reduced cell growth and G2 arrest that led to cell death (Figure S3E), indicating an apoptotic sensitivity toward proliferating cells, as demonstrated previously. Further, we analyzed the pluripotency expression in hPSC cultures. hPSCs with equally high pluripotency marker expression of 90% (NANOG, OCT4a, TRA-1-60, and SOX2) showed large variations across their S/G2/M cell-cycle profiles (Figure 3D). An increase in cell culture confluency from 70% to 85% first led to a decrease of 8% in the S/G2/M cell-cycle profile, and a further increase of cell culture confluency from 85% to 90% decreased the expression of pluripotency markers (Figure 3E). In three tested hPSC lines cultured at >90% confluency, reduced S/G2/M cell-cycle profiles (three out of three cell lines) and pluripotency (one out of three cell lines) were observed when compared with <70% culture confluence (Figure S3F). This finding indicates that the loss of pluripotency might have contributed to the changes in cardiac differentiation. Hence, we compared NANOG and OCT4a expression with Troponin T expression after differentiation (Figure 3F; Table S2). The data showed a positive asymptotical correlation of NANOG and OCT4a expression with Troponin T expression. Although NANOG and OCT4a cell populations above 85% are likely to induce the cardiac differentiation of >50% of the Troponin T population, these markers were not sufficient to predict the cardiac differentiation efficiency across all cell lines. The cell cycle proved to be more sensitive than NANOG and OCT4a expression for indicating changes in cell culture and the resulting Troponin T expression (Figures 3D and 3E). Hence, we correlated the different cell-cycle phases with Troponin T expression. The S/G2/M and S-phase cell-cycle profiles showed a linear correlation with the cardiac differentiation results (R^2^ = 0.6/0.8) across several cell lines (Figure 3G; Table S2). The data show that an S phase cell-cycle percentage of above 21% in multiple hPSC lines is indicative of the induction of >50% Troponin T expression with an optimized CHIR concentration.

In summary, a combined high S/G2/M cell-cycle profile percentage (S phase >21%, G2/M >20%) and a pluripotency of >85% (NANOG and OCT4a) were indicative markers for high-efficiency cell lines, which were likely to achieve a cardiac differentiation of over 50%–90% with a single CHIR induction. Moreover, reductions of the S/G2/M cell cycle by 5%–7% based on the initial cell culture confluency led to a loss of cardiac differentiation efficiency, which was restored by reducing the CHIR concentration by 2 μM in monolayers and 1.5 μM in EB culture experiments. We conclude that a high and stable S/G2/M cell-cycle profile in hPSCs is an important indicator of cell line selection, in addition to pluripotency markers, and an essential factor for cardiac differentiation reproducibility with CHIR.

### GSK Inhibition with CHIR Is Controlled by the Wnt Signaling Pathway via TCF Levels

In this section, the protein expression levels of the Wnt signaling pathway were examined after CHIR induction. We measured quantitatively the time- and dose-dependent activation of Wnt protein expression, such as TCFs and β-catenin, in EB and monolayer cultures of different densities and identified the cellular locations of the expressed proteins. We hypothesize that cell culture and cell-cycle differences have an effect on β-catenin/Wnt and TCF regulation upon CHIR99021 induction.

**Figure 4:**
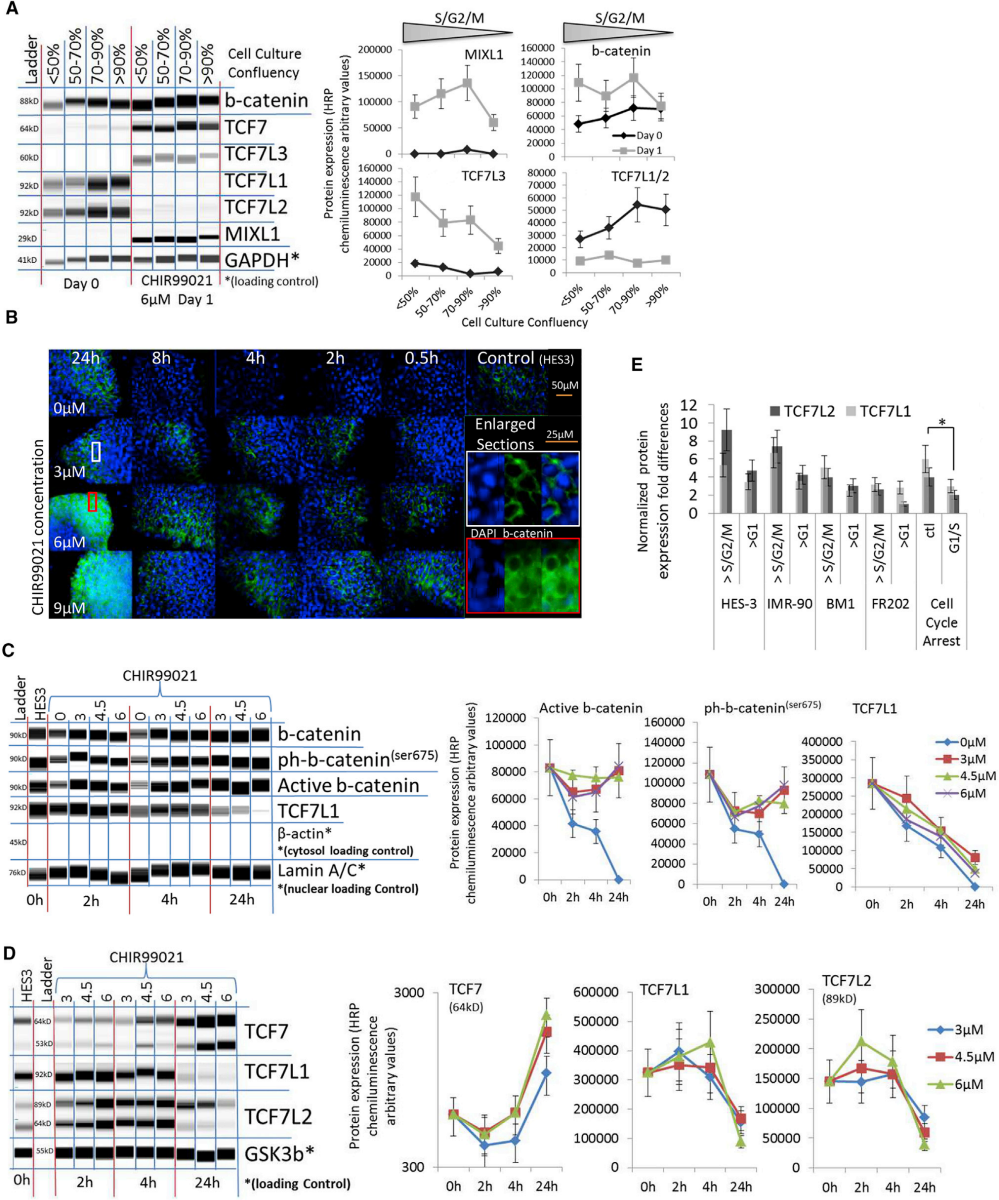
β-Catenin and TCF Modulations after CHIR Induction (A) Whole-cell protein blot expressions and quantitative analyses of HES3 cells cultured at <50%, 50%–70%, 70%–90%, and >90% cell culture confluency after CHIR induction. (B) Fluorescence microscopy of immuno-cytochemistry-stained HES3 cells against active β-catenin (green) and DAPI (blue) after CHIR induction. (C) Nuclear fraction protein blot expressions and quantitative analyses of HES3 EBs after CHIR induction. Note sample CHIR 0 μM degraded after 24 hr. (D) Whole-cell protein blot expressions and quantitative analyses of HES3 EBs after CHIR induction. (E) Normalized TCF7L1/2 protein expression folds of hPSCs cultured at 90% cell confluency with increased G1 cell-cycle phase compared with 70% cell confluency, with an increased S/G2/M and average TCF7L1/2 protein expressions of hPSCs after G1/S cell-cycle arrest (n = 3, ^∗^p ≤ 0.05).

The S and G2 cell-cycle phases are known to induce β-catenin/Wnt pathway signaling via TCF modulations (Ding et al., 2014), which are a potent driver of cardiac differentiation. We measured TCF levels and expression of the primitive streak development marker MIXL1 in EB and monolayer cultures of different confluency and various cell lines with high to low S/G2/M cell-cycle profiles (Figure 4A, S4A, and S4B). TCF7L1/2 was expressed in hPSCs and downregulated in all cell lines and culture conditions after CHIR induction (Figures 4A, S4A, and S4B). Cell lines and culture conditions with a comparatively higher S/G2/M cell-cycle profile showed increased MIXL1 and T-Brachyury and Wnt activator TCF7/TCF7L3 expression when compared with high cell seeding conditions and cell lines with low S/G2/M cell-cycle profiles (Figures 4A, S4A, and S4B). Interestingly, β-catenin was not upregulated or weakly upregulated in dense culture conditions with a lower S/G2/M cell-cycle percentage (Figures 4A, S4A, and S4B). The current hypothesis in Wnt-induced cardiac differentiation focuses on a Wnt/β-catenin-driven mechanism of nuclear β-catenin transfer and accumulation (McCubrey et al., 2014, Ye et al., 2012). Active β-catenin was located in the cellular membrane and did not increase during the first 4–8 hr of CHIR treatment (Figures 4B and S4C). β-Catenin accumulated in the cytosol after 24 hr when 6–9 μM CHIR was applied (Figure 4B). Interestingly, the nuclear protein expression levels of the β-catenins showed no significant changes with 3–6 μM CHIR treatment in EBs (Figures 4B and 4C). However, a CHIR concentration of >6 μM induced β-catenin and transcriptional β-catenin expression in monolayer cultures (Figures S4A and S4B). Nuclear β-catenin translocation was observed with the FR202 cell line at 15 μM CHIR (Figure S4D). Nuclear protein expression of β-catenin was limited in IMR90 cells but increased significantly in the FR202 cell line (Figures S4E and S4F). Moreover, we observed that cell-cycle profile differences of 5%–6% inhibited β-catenin expression in IMR90 cells and limited β-catenin expression in the FR202 cell line; nevertheless, TCF and primitive streak markers were expressed (Figures S4A and S4B). Moreover, we observed a significant decline of nuclear and cytosolic TCF7L1 levels 4 or 6 hr after CHIR induction in EB and monolayer cultures with all tested CHIR concentrations and the successive induction of primitive streaks and the TCF7 and TCF7L3 markers 24 hr later (Figures 4A, 4C, S4A, S4B, S4E, and S4F).

The transcription of TCF7 and TCF7L3 is induced by β-catenin nuclear translocation, whereas TCF7L1/2 is a negative regulator of the nuclear Wnt signaling pathway (Yi et al., 2011). Thus, we measured TCF7, TCF7L1, and TCF7L2 protein expression levels during CHIR induction. The expression of TCF7L1 and TCF7L2 decreased, while TCF7 increased in a dose-dependent manner in EB and monolayer cultures with the HES3, IMR90, and FR202 cell lines (Figures 4C, 4D, S4E, and S4F). Moreover, we measured the TCF7L1/2 protein expression of >90% confluent (>G1 cell-cycle phase) hPSCs and 70% confluent hPSCs with a relatively increased S/G2/M phase (Figure 4E). TCF7L1/2 expression was decreased in dense cell culture conditions (>G1 cell-cycle phase) in most cell lines. Dense culture conditions required lower (1.5–2 μM) CHIR concentrations to induce cardiac differentiation efficiently, as shown previously (Figures 3B and S3D). The data indicate that cell culture conditions affected the cell cycle of hPSCs, which modulated the levels of Wnt, inhibiting TCF7L1/2 proteins; thus, the CHIR concentration for cardiac differentiation had to be adjusted. Furthermore, we confirmed that G1/S cell-cycle arrest reduced TCF7L1/2 expression in IMR90 and FR202 cells and increased transcriptional β-catenin expression (Figure 4E and S4G). Moreover, cyclin A and cell-cycle checkpoints were expressed after cell-cycle arrest. The data showed that TCF7L1/2 was modulated by cell-cycle manipulations, but additional factors, such as β-catenins, cyclins, and cell death, are part of the complex mechanism of Wnt regulation during CHIR-induced differentiation.

In summary, modulations of β-catenin levels after CHIR induction were cell line- and cell culture dependent. β-Catenin translocated from the membrane to the cytosol and increased β-catenin levels in hPSCs when cultured at 50%–70% culture confluency with over >6 μM CHIR. Interestingly, hPSCs with a culture confluency of 90% and a reduced S/G2/M cell-cycle profile showed a small increase in β-catenin levels. Nevertheless, all tested concentrations and culture conditions induced primitive streaks, the early mesoderm and TCF7 and TCF7L3 marker expression. More importantly, the Wnt regulators TCF7L1 and TCF7L2 decreased in the nuclear fraction prior to TCF7 and TCF7L3 expression. Overall, TCF levels responded within 4–24 hr to CHIR induction in a manner dependent on dose and culture conditions, indicating that TCFs play a key role as a Wnt regulatory mechanism.

### GSK Inhibition with CHIR Enhances Mesoderm Differentiation Independent of Cell Culture and Cell-Cycle Manipulation

In this section, primitive streak and early mesoderm development was examined in terms of transcription factor and protein expression. We measured the development of EB and monolayer cultures after CHIR induction at various doses and with cell-cycle arrest and compared the results with the cardiac differentiation efficiency. We hypothesize that both the CHIR doses and the cell cycle affect mesoderm development and therefore cardiac differentiation.

**Figure 5:**
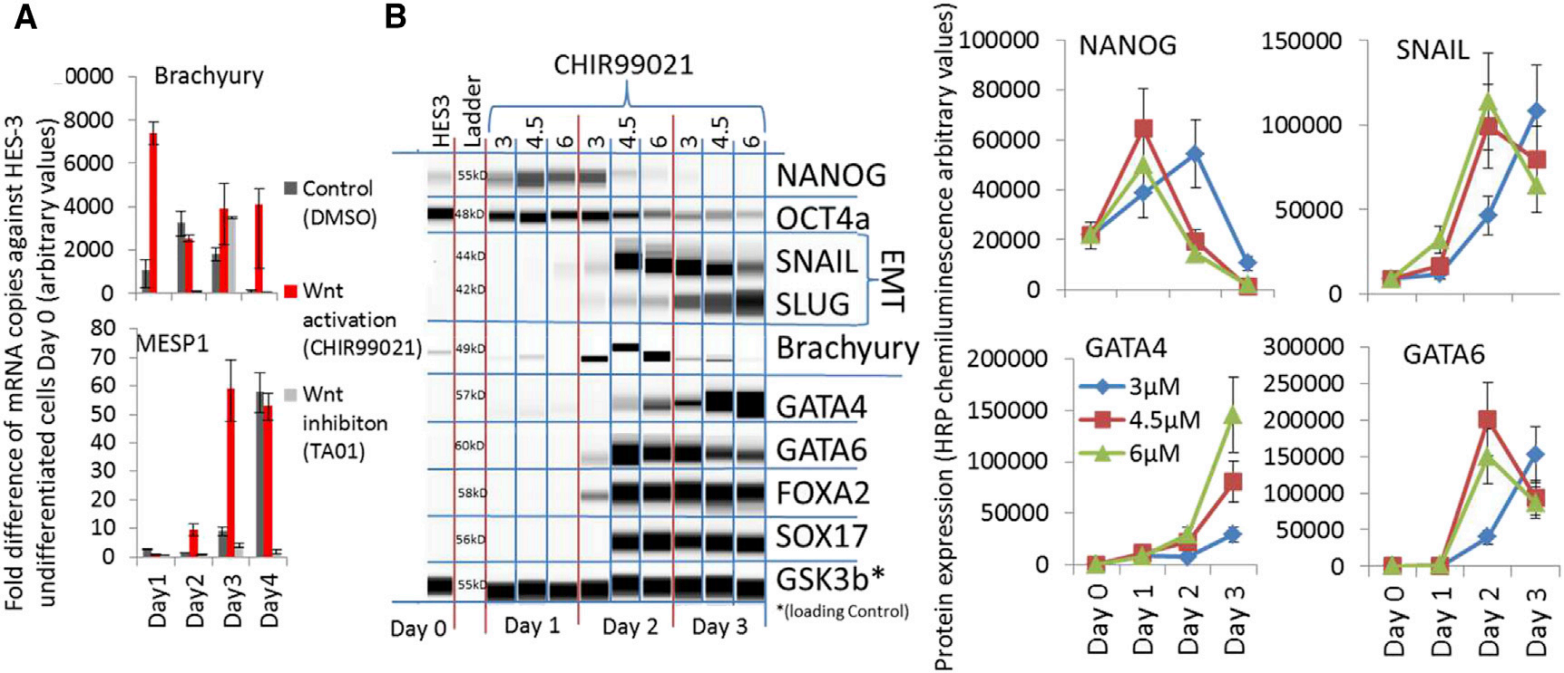
Acceleration of Mesodermal Development after CHIR Induction (A) qPCR analyses of fold increase/decrease over HES3 control after treatment of HES3 EBs with 3 μM CHIR, DMSO control, and 5 μM Wnt inhibitor TA-01 for 24 hr (n = 3). (B) Whole-cell protein blot expressions and quantitative analyses of HES3 cells and differentiating EBs after CHIR induction.

The expression of T-Brachyury was induced on day 1 after CHIR treatment, followed by MESP1 expression on days 2–4 in hPSC EBs (Figure 5A). These mesodermal inductions were also observed in the non-treated (DMSO) and Wnt-inhibited EBs (TA01) cultures, with a delay of 24–48 hr (Figure 5A). Further, we measured the dose dependency of CHIR in endo-mesoderm development. CHIR concentrations (4.5–6 μM) induced endothelial-mesenchymal transition (Slug, Snail), endo-mesoderm development (GATA4, -6, FOXA2, and SOX17) and pluripotency decline (NANOG and OCT4a) on day 2, whereas a lower CHIR concentration of 3 μM resulted in a 24-hr delay in the protein expression and persistent expression of pluripotent markers (Figure 5B). Similar results were obtained using a microcarrier-based bioprocess culture system with the IMR90 cell line (Figure S5A). CHIR dose-dependent primitive streak induction was also confirmed with four additional lines (X.13, IMR90, 4Skin, and Donor 5) in monolayer experiments (Figure S5B). The induction of T-Brachyury and MIXL1 was strongly cell line dependent (Figure S5B). Interestingly, cell confluency differences with a 5%–6% S/G2/M cell-cycle profile difference did not affect T-Brachyury expression, as shown previously (Figures S4A and S4B). Therefore, we tested the effect of cell-cycle arrest on mesoderm development. The flow cytometry profiles of T-Brachyury and PDGFRa were largely unaffected by G1/S phase-arrested hPSC cultures, but Troponin T expression showed strong differences between the tested conditions (Figure S5C). The Troponin T expression did not correlate with T-Brachyury, MIXL1, or PDGFRa expression (Figures S5B and S5C).

In summary, CHIR-induced primitive streak and mesoderm development (MIXL1, MESP1, T-Brachyury, GATA4, and PDGFRa) in hPSCs maintained in EBs, monolayers, and microcarrier-based aggregate cultures. The onset and progression of mesoderm development was CHIR concentration and cell line dependent. Mesoderm development was less affected by hPSC culture confluency/density and cell-cycle changes, but cardiac differentiation was strongly affected by confluency/density and cell-cycle changes.

### TCF7L1 and TCF7L2 Expression Is Prolonged by Small-Molecule Wnt Inhibitors to Induce the Expression of Mesoderm Markers and the Cardiac Progenitor NKX2-5

In this section, the depletion of CHIR and the time and dose effects of Wnt inhibitors post-CHIR induction were examined. Wnt pathway proteins, TCF expression, and mesoderm and early cardiac development markers were measured. Reversing the effect of CHIR is thought to limit Wnt signaling and induce differentiation.

**Figure 6:**
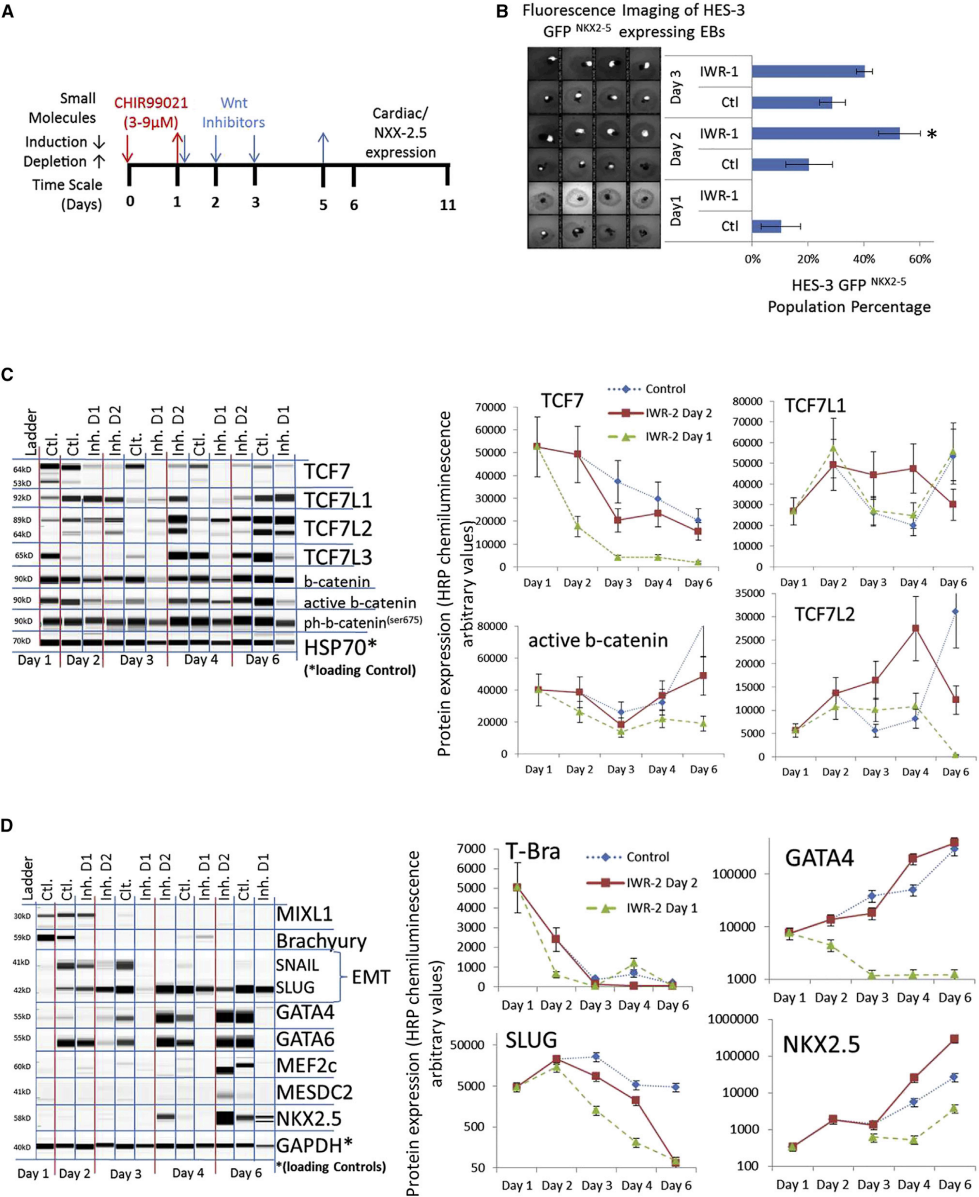
TCF7L1/2 Expression Regulates Wnt Inhibition and Early Cardiac Differentiation (A and B) Induction timing for CHIR and Wnt inhibitors during the differentiation process (A). Differentiated HES3 EBs on day 11 expressing GF/NKX2-5 area in white and GFP/NKX2-5 population percentage (B). HES3 EBs were cultured with 6 μM CHIR for 24 hr and with/without 2.5 μM IWR-1 induction on day 1–3 (n = 4, ^∗^p ≤ 0.05). (C) Whole-cell protein blot expressions and quantitative analyses of HES3 EBs after 6 μM CHIR induction (ctl.) and 2.5 μM IWR-1 inhibition at day 1 (inh. D1) and day 2 (inh. D2). (D) Protein blot expressions and quantitative analyses of whole-cell HES3 EBs after 6 μM CHIR induction (ctl.) and 2.5 μM IWR-1 inhibition at day 1 (inh. D1) and day 2 (inh. D2).

Cardiac differentiation can be enhanced by external Wnt inhibition (IWR-1) when applied 1–3 days after CHIR induction in EBs (Figures 6A and 6B; Table S1) and monolayer cultures (Table S2). We measured a strong increase of the mRNA expression levels of the Wnt-negative regulator DKK-1 after CHIR induction (Figure S6A), but protein expression levels of DKK-1 and ph-GSK3β^(ser9)^ were low (Figure S6B). Therefore, we analyzed the protein expression levels of TCFs in three EB cultures with IWR-1 (2.5 μM) induction on day 1 (0% GFP/NKX2-5) or day 2 (50% GFP/NKX2-5) or without IWR-1 induction (25% GFP/NKX2-5) as the control condition (Figure 6B). The TCF7L1 and TCF7L2 levels increased after CHIR withdrawal on day 2 but returned to their previous levels on day 3 (Figure 6C). IWR-1 prolonged TCF7L1 and TCF7L2 expression when applied on day 2 (Figure 6C). When applied on day 1, β-catenin, TCF7, primitive strike, mesoderm, and EMT markers were downregulated early by IWR-1, indicating that TCF7 and β-catenin levels might have affected the expression of these direct Wnt gene targets, which disturbed further mesoderm marker expression (Figure 6D). IWR-1 induction on day 2 reduced GATA-4, GATA-6, and EMT on day 3 but induced significantly higher expression of GATA-4 on day 4 and an early onset of the cardiac progenitor marker NKX2-5 (Figure 6D). Other muscle mesoderm markers (MEF2c and MESDC2) were expressed on day 6 (Figure 6D).

In summary, this experiment shows that CHIR depletion leads to a transient increase of Wnt, inhibiting TCF7L1 and TCF7L2 expression levels. Moreover, IWR-1 prolonged the expression of TCF7L1 and TCF7L2, which supported cellular TCF regulation and increased specialization toward the mesoderm and early cardiac lineages.

## Discussion

### Cytotoxicity of the GSK Inhibitor CHIR in hPSC Cultures Alters Cardiac Differentiation

The importance of small-molecule GSK3β inhibitors is evident in hPSC differentiation and may become an industrial standard for the development of cell-based therapy. However, GSK3β inhibition with CHIR in hPSC lines (n > 12) is cytotoxic to cell cultures, especially to those with a low S/G2/M cell-cycle phase. hPSCs are characterized by a high G2 phase (Kapinas et al., 2013). An increase of the G1 phase and a loss of the G2/M cell-cycle phase is due to colony culture conditions and ECM dependency, which restrict cell expansion at the center of the colonies (Jacobs et al., 2016). An accumulation of high sub-G1 and G1 cell-cycle phases in hPSCs is likely due to medium acidification of densely cultured cells (Jacobs et al., 2016), which is known to affect cell growth, metabolism (Chen et al., 2010), and pluripotency (Gupta et al., 2017), and to induce genotoxic stress in hPSCs (Bárta et al., 2010). Cell death occurred upon CHIR induction, especially with high-cell-density cultures. CHIR-induced cyclin D1 expression in hPSCs. Cyclin D1 is known to activate essential genes for S phase entry and DNA synthesis (Lim and Kaldis, 2013), and overexpression could lead to rapid cell growth and replication stress under conditions of restricted mitogenic signaling, bypassing key cell-cycle checkpoints (Qie and Diehl, 2016). Therefore, it can be assumed that CHIR99021-induced expression of cyclin D1 forced the hPSCs into cell-cycle progression, leading to a high rate of premitotic apoptosis due to an accumulation of genetic instability after exposure to genotoxic stress in acidified medium from high-density cell cultures. Although hPSCs have a robust DNA damage repair mechanism prior to G2 entry (Vitale et al., 2017), they have been shown to fail single-strand DNA damage repair due to replication stress and to induce apoptosis during the intra-S cell-cycle phase (Desmarais et al., 2012, Desmarais et al., 2016) or lose pluripotency (Vitale et al., 2017). Moreover, the S cell-cycle phase correlated with cardiac differentiation efficiency. In this regard, culture conditions (e.g., cell density, cell passaging, and media) define an initial genomic hPSC state that translates to the differential response to CHIR (Gonzalez et al., 2016). Therefore, it can be assumed that these intrinsic and extrinsic factors induce replicative stress, leading to genomic instabilities. Upon CHIR induction, an immediate cytotoxic effect is present due to cell-cycle progression. Moreover, cardiogenic efficiency with CHIR depends strongly on the genomic stability of hPSCs and could be an indicator of cardiac differentiation efficiency and other CHIR-induced differentiation methods.

### TCFs as Wnt Regulators in Mesoderm and Cardiac Differentiation

Wnt induction is directly involved in primitive streak patterning and mesoderm differentiation (e.g., MIXL1, T-Brachyury, ISL1, and BMP4) (Hodar et al., 2010). The accumulation of nuclear β-catenin is thought to induce Wnt gene transcription. Induction with >7 μM CHIR has been shown to induce cytosolic and nuclear β-catenin accumulation (Kempf et al., 2016, Mendjan et al., 2014). However, we show that β-catenin levels are not significantly changed with <6 μM CHIR in EB differentiation protocols. Interestingly, primitive streak and mesoderm marker expression did not depend on the cytosolic or nuclear enrichment of β-catenin. Moreover, TCF levels responded to CHIR in a concentration-dependent manner via GSK3β inhibition. TCFs consist of four paralogs in humans. TCF7L3 expression promotes WNT target activation, whereas TCF7L1 expression represses WNT targets. The expression of TCF7 and TCF7L2 can be associated with both actions depending on the cellular system (Cadigan, 2012). TCF7L1 and TCF7L2 were highly expressed in hPSCs and decreased upon CHIR stimulation, whereas TCF7 and TCF7L3 were weakly expressed but increased strongly after GSK3β inhibition with CHIR. This finding indicates that TCF7L1 and TCF7L2 act as Wnt repressors and that TCF7 and TCF7L3 are Wnt activators in hPSCs. A potential mechanism was observed with a mouse embryonic model in which TCF7L1 was inactivated by β-catenin with CHIR and WNT3a to induce gene transcription (Shy et al., 2013). Interestingly, our data indicate that TCF regulation is induced by the inhibition of GSK3β via CHIR prior to β-catenin nuclear induction. However, it remains unclear how Wnt-regulated transcription targets of primitive streak and mesoderm development are induced. A possible explanation could be that β-catenin was metabolized prior to detection by interacting with TCF7L1/2, as described by Shy et al. (2013) and Hikasa and Sokol (2011). This possibility would explain why cell lines with low TCF7L1/2 expression, such as FR202, exhibit β-catenin translocation, whereas HES3 and IMR90 lacked evidence. Further, Wnt transcription could be induced by pre-existing nuclear expression levels of β-catenin upon depletion of the nuclear Wnt transcription inhibitors TCF7L1 and TCF7L2. This process would lead to Wnt gene target transcription, including TCF7 and TCF7L3 expression. A delayed nuclear induction of β-catenin in combination with the TCF7 and TCF7L3 proteins would amplify Wnt gene transcription and induce a chain of developmental signals toward cardiac differentiation. Although we demonstrate the importance of TCFs here, the role of β-catenin remains paramount, as nuclear levels of β-catenin are essential for the modulation of Wnt transcription and cardiac differentiation.

### Dynamic Mechanism of Mesoderm-Cardiac Differentiation

**Figure 7:**
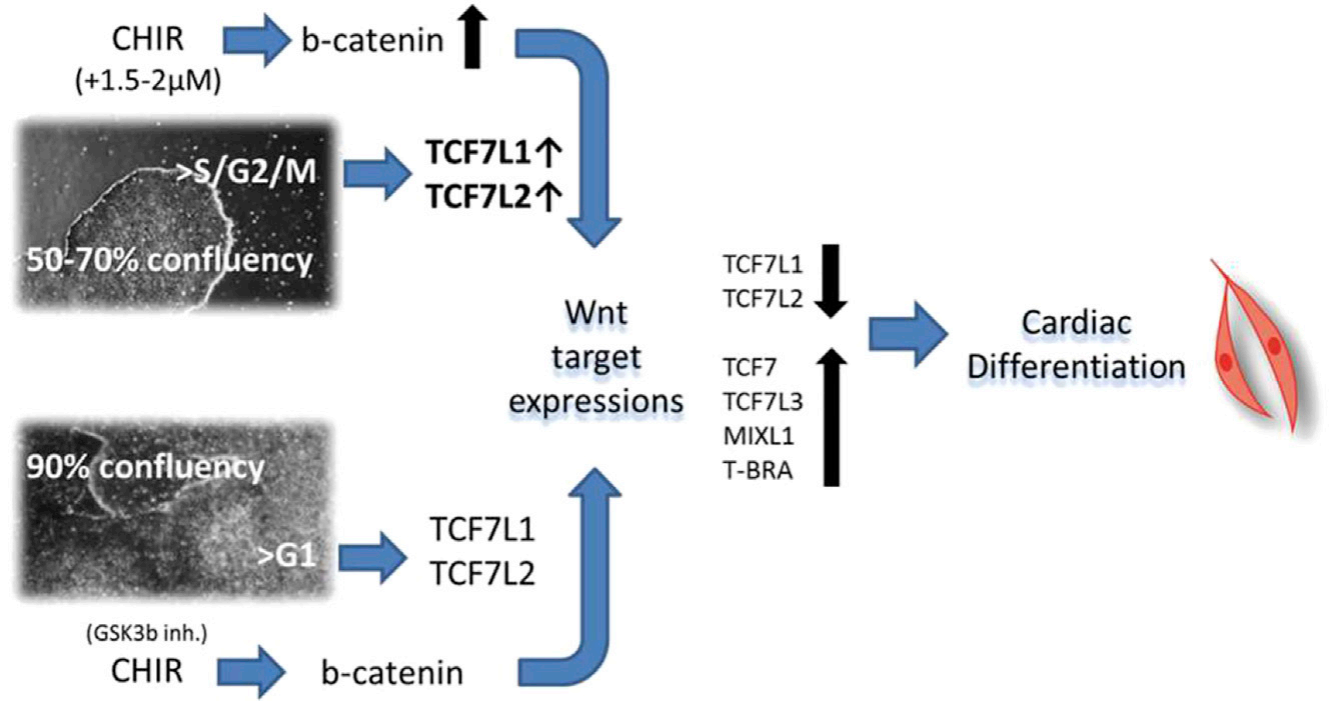
CHIR Modulation in Varying Cell Culture Densities A lower cell culture confluency (50%–70%) of hPSCs showed an increased S/G2M cell-cycle phase which elevated the expression levels of Wnt inhibitors TCF7L1/2 when compared with higher cell culture densities (90%). CHIR induction required an additional 1.5–2 μM to induce higher β-catenin levels via GSK3b inhibition in order to suppress TCF7L1/2, and activate sufficient levels of the Wnt gene target expression (TCF7, TCF7L3, MIXL1, and T-BRA). A successive time-sensitive Wnt modulation lead to a comparable cardiac differentiation in both cell culture formats.

The CHIR time course and dose can delay or accelerate mesoderm development and lead to a variety of lineages. CHIR activated Wnt by decreasing TCF7L1 and TCF7L2 and inducing TCF7 and TCF7L3. A low CHIR concentration has been shown to lead to endoderm development, which can induce hepatocyte differentiation (Siller et al., 2015). Prolonged or 2- to 3-fold higher CHIR concentrations induced high expression of primitive streak and mesoderm markers, followed by endothelial-mesenchymal transition and cardiovascular progenitor development. A critical step was CHIR withdrawal, which leads to Wnt inhibition and was marked by a transient TCF7L1 and TCF7L2 induction. Wnt inhibition was shown to be essential for suppressing the cardiac deregulator MSX1 (Rao et al., 2015), which induced the cardiovascular markers GATA4 and NKX-2.5. Wnt inhibition induced by CHIR depletion and TCF7L1 and TCF7L2 upregulation was insufficient to induce cardiomyocytes when the initial CHIR doses were too high or temporally overextended. Wnt inhibitors, such as IWP-2, IWR-1, and TA01/02, have been found to support cardiac differentiation during this particular time window (Laco et al., 2014). The transient expression of the Wnt repressors TCF7L1 and TCF7L2 was prolonged for the duration of IWR-1 induction, leading to controlled cardiac differentiation. However, the initial variations in culture conditions (cell confluency and cell cycle) limited the reproducibility. The S and G2 cell-cycle phases are known to induce β-catenin/Wnt pathway signaling via TCF modulations (Ding et al., 2014). A mid to low cell culture confluency (50%–70%) resulted in increased S/G2/M percentages and TCF7L1/2 expression and required lower CHIR doses to induce cardiac differentiation (Figure 7). CHIR induces β-catenin, which interacts with and depletes TCF7L1/2 (Shy et al., 2013) to induce timely Wnt and mesoderm induction, leading to cardiac differentiation (Figure 7). Variations in TCF7L1/2 levels and/or the cell cycle will therefore lead to alternative differentiation results when CHIR concentrations are kept constant. Moreover, pluripotency marker expression levels were often unaffected by cell-cycle changes. Thus, cell-cycle validation is crucial for stem cell quality and differentiation efficiency.

In conclusion, GSK inhibition via CHIR manipulates the cell cycle and TCF/Wnt regulation simultaneously. Our findings not only demonstrate how GSK inhibition interconnects the cell-cycle and cell-fate decisions to direct mesoderm and cardiomyocyte development in hPSCs, but also help to elucidate how the proliferation and cell death of hPSCs are induced during differentiation. Moreover, we elucidate the importance of the cell cycle in CHIR-driven differentiation methods. Thus, we propose that the cell-cycle profile should be part of the regular quality control criteria for hPSCs in order to improve experimental consistency and cell line selection for high differentiation efficiency to cardiac and other mesoderm-derived cell types.

## Experimental Procedures

### General Mesoderm-Cardiac Differentiation Protocol

The protocol was adapted from Lian et al. (2012). In brief, when hPSCs maintained on Geltrex-coated plates achieved the desired confluence, the cells were treated with the GSK3β inhibitor CHIR (Selleckchem) in differentiation medium for 24 hr. The differentiation medium was either phenol red-free bSFS medium for single EB and Aggrewell differentiation or RPMI/B27-insulin (Life Technologies) for monolayer and microcarrier differentiation. The differentiation medium was changed to remove/reduce CHIR concentrations during the first 3 days. An optional treatment with Wnt inhibitors, such as IWR-1 (Tocris), IWP-2 (Stemgent), or TA01 (Tocris), followed on day 2/3. Wnt inhibitors were removed during the medium change on day 5/6. The cells were maintained in differentiation medium with daily medium changes. The cells were incubated at 37°C in a humidified atmosphere with 5% CO_2_. All small molecules were solubilized in DMSO (Sigma). The optimization of the protocol can be found in Table S1 and the Supplemental Experimental Procedures.

## Author Contributions

F.L. designed the experiments. F.L., T.L.W., F.J.K., and Q.Z. performed the experiments. F.L. and T.L.W. performed the computational analysis of the data. F.L. interpreted the experiments and data; additionally, R.S. interpreted the cell-cycle-related experiments and data. F.J.K., S.T., and A.C. provided experimental data related to cardiac bioprocessing and technology translation. F.L. wrote the paper with input from S.O., A.C., C.L.L.C., and S.R.

## Supplemental Information

Document S1. Supplemental Experimental Procedures, Figures S1–S7, and Tables S1–S3

Document S2. Article plus Supplemental Information
