# Supplementary material for: Unraveling the Inconsistencies of Cardiac Differentiation Efficiency Induced by the GSK3β Inhibitor CHIR99021 in Human Pluripotent Stem Cells
Source: Stem Cell Reports. 2018 Apr 26;10(6):1851–66. doi: 10.1016/j.stemcr.2018.03.023 (PMC5989659; doi:10.1016/j.stemcr.2018.03.023)
Supplement: Document S1. Supplemental Experimental Procedures, Figures S1–S7, and Tables S1–S3 [file mmc1.pdf]

Stem Cell Reports, Volume *10*

Supplemental Information

Unraveling the Inconsistencies of Cardiac Differentiation Efficiency Induced by the GSK3β Inhibitor CHIR99021 in Human Pluripotent Stem Cells

Filip Laco, Tsung Liang Woo, Qixing Zhong, Radoslaw Szmyd, Sherwin Ting, Fahima Jaleel Khan, Christina L.L. Chai, Shaul Reuveny, Allen Chen, and Steve Oh

Supplemental Experimental Procedures

Work flow, method and analyses of single embryoid body cardiac differentiation

We established a high throughput single embryoid body screening method with a cardiac reporter cell line HES3 NKX2-5$^{eGFP/w}$ to measure embryoid body formation, cell growth, cell cycle, cytotoxicity, mesoderm to cardiac differentiation and cellular signalling pathway of Wnt/beta-catenin. Using this system we investigated the effect of the dose and induction timing of GSK3β inhibitor CHIR and application of subsequent Wnt inhibitors on the process of mesoderm and cardiomyocyte differentiation. Moreover, the high throughput system enabled the investigation of the effect of different cell culture condition such as the initial cell culture densities and different cell lines during the embryoid body formation and differentiation process (Figure S7A). Critical findings were confirmed and repeated with a panel of 11 hPSC lines in monolayer culture systems.

In our workflow we evaluated monolayer cell cultures of human embryonic reporter stem cells line HES3 NKX2-5$^{eGFP/w}$ for pluripotency, viability, cell culture density and cell cycle. The cell cultures were dissociated and force aggregated to form single EBs. Several attempts to generate EBs by simply force aggregation of suspended cells failed; addition of Rock inhibitor Y27632 allowed HES3 to form aggregates but the resulting EBs were brittle and did not differentiate to cardiomyocytes (Figure S7B). A combination of CHIR, Y27632 and extracellular matrix (ECM) substrates such as Matrigel® was able to improve single EB formation (Figure S7B). We optimised time and dose concentration of HES3 derived cardiomyocytes and were able to show that single induction of CHIR induced cardiac differentiation efficiency of >85% Nkx2-5 expression (Figure S7C, Table 1S). The small molecule Wnt inhibitors were found not to be essential as their Wnt inhibitory function could be replaced by timely CHIR depletion. However, our full data set showed that small molecules inhibitors such as IWR-1 increase significantly the NKX-2.5 expression when CHIR was applied at higher concentration or for extended time course (Table S1). In addition, timely induction of IWR-1 was essential for the cardiac differentiation efficiency of certain cell lines in monolayer experiments (e.g. 4skin, DF6) which followed Lian et al. 2011 method (Table S2). A standard protocol was applied with an initial supplementation of Matrigel® (50mg/ml), 5µM Y27631 and 6 µM CHIR for 24 h to induce a robust EB formation and differentiation (Figure S7D). During the differentiation process, EBs were lysed at early and late developmental stages (Day 0-6) and protein levels were quantitatively analysed by antibody detection via capillary electrophoresis western blots (Figure S7A).

EB size was evaluated by measuring the whole EB area (Figures S7E-F). The measured area was found to correlate with the cell number count of dissociated EBs at 10100 cells/0.1mm^2 (Figure S7G).

GFP/NKX2-5 expression is an essential marker for progenitor and early cardiomyocytes (Lyons et al., 1995). Fluorescence microscope assisted screens of EBs were able to identify and calculate the portion of the HES3 NKX2-5$^{eGFP/w}$ GFP area of the whole EB area (Figures S7E-F). Flow cytometry analysis showed that HES3 NKX2-5$^{eGFP/w}$ GFP staining was found to co-stained with Troponin T (Figure S7G). Thus cardiac differentiation efficiency of HES3 NKX2-5$^{eGFP/w}$ reporter cell line was evaluated from day 6 by imaging the GFP expression area of the EB (GFP area). GFP image analyses were found to correlate with GFP flow cytometry of single cell dissociated EBs (r^2= 0.91) (Figure S7G).

Cell Culture

Embryonic stem cell lines HES3 and HES3$^{NKX2-5eGFP/w}$ (reporter cell line for cardiac differentiation was kindly provided by David Elliot Monash University Australia), WA01 (H1), WA07 (H7), human induced pluripotent stem cell (iPS) lines iPS(IMR90)-1 (IMR-90), iPS(Foreskin)-1 (4Skin), iPS-DF6-9-9T (DF6), IISH1i-BM1 (BM1),and IISH3i-CB6 (CB6) (WiCell Research Institute), Donor 5 and X.13 (derived at the Institute of Molecular and Cell Biology, Singapore, kindly provided by Dr. Jonathan Loh) and FR201 and FR202 (derived at the Bioprocessing Technology Institute, Singapore, kindly provided by Dr. Alan Lam) were cultured on Geltrex® coated tissue culture plates. All cell lines used for Aggrewell® or Single EB experiments were cultured in Essential 8™ media (Life Technologies). All cell lines used in monolayer and microcarrier experiments were cultured in mTeSR™1 (Stemcell Technologies). HES3 and HES3$^{NKX2-5eGFP/w}$ were cultured in both media types for comparison in different

differentiation protocols. Media was refreshed daily and cultures were passaged mechanically with STEMPRO® EZPassage tool (Life Technologies) every 3–6 days at about 70% cell plate confluency. Cell cultures were incubated at 37°C in a humidified atmosphere with 5% CO2.

Single and Aggrewell® EB formation

Protocol was adapted from (Ng et al., 2005) and the Stemcell Technologies™ manual for Aggrewell® EB formation. Briefly: HES3$^{NKX2\text{-}5eGFP/w}$ cells were dissociated with TrypLE (Invitrogen) and seeded at 1.5 × 10^4 cells/well in ultra-low attachment 96-well clear round bottom plates (Corning) in bSFS medium 150µl/Well. bSFS composition (Ting et al., 2013) : DMEM supplemented with 2mML-glutamine, 0.182 mM sodium pyruvate, 1% non-essential amino acids, 5.6 mg/l transferrin (all from Life Technologies), 0.1 mM β-mercaptoethanol (Sigma), 20 µg/L sodium selenite (Sigma), 0.25% (w/vol) Bovine Serum Albumin (MP) and 0.25% (w/v) Hysoy (Sheffield Bioscience)] HES3$^{NKX2\text{-}5eGFP/w}$, H1, H7, IMR-90, DF6 and CB6 cells were seeded at 4.5× 10^6 cells/well in Aggrewell® plates (Corning) in bSFS medium 2.5ml/Well. Cells were forced to aggregate by spinning down at 800 rpm for 1min and incubated for 24 h with 1.5-12 µM CHIR99021 (Selleckchem), 5 µM Y27632 (Selleckchem) and 50 µg/ml Matrigel® (BD, UK) at 37°C and 5% CO2 to allow EB formation. Media changes and initial cell seeding in 96-well plates was assisted by liquid handling unit Viaflow Assist (Integra, US) for volume and cell seeding accuracy.

Microcarrier HES3 Culture and Cardiac Differentiation

Protocol is described by (Ting et al., 2014) and available at http://dx.doi.org/10.1016/j.scr.2014.06.002

Fluorescence microscopy assisted EB screening

Dual images of phase contrast and fluorescence for green fluorescence protein detection (GFP) were taken of each EB in 96-wells U-bottom plates with an inverted Nikon Eclipse Ti-E microscope b/w camera. The 96-well plate was mounted on a fully automated movable stage which allowed precise positioning and focusing of each well and EB (Nikon). The culture plates were kept at 37˚C with 5% CO_2 via an on-stage incubator. A full screen of 96-wells took 3 min with our high speed processing and storage hardware (Nikon). Images were taken at a resolution of 1.62 µm/pixel at fixed settings for phase contrast imaging: light intensity 60%, exposure 2 ms, for fluorescence imaging: light intensity 29%, exposure 700 ms. Images were saved as .tif stack format for further processing and quantification with Image J®. Images were processed to quantify the GFP size of HES-3 *NKX2-5*$^{eGFP/w}$, and EB size with Image J®. Briefly: Fluorescence and phase contrast images stacks were reduced to 8-bit greyscale, background subtracted, optionally inverted, processed to black/white images with image threshold. EB or GFP pixel area was calculated with Image J® particle analyser. Pixel area was calculated back to area (mm^2).The area was converted to the actual cell number my measuring the dissociated cell numbers of different sized EBs. A pixel area of 0.1 mm^2 = 10,100 Cells (±951 Cells). GFP percentage was calculated following the equation: GFP percentage = (GFP area)/(EB area). Only EBs between 0.08-0.2 mm^2 were used for GFP area calculations to eliminate technical measurement errors. 4-8 EBs were used as technical replicates per tested condition to allow for statistical analyses.

Flow Cytometry

Cells were harvested, and dissociated into single cells using TrypLE™ Express (Life Technologies) for 7–10 min in a heating block under constant shaking (Thermomixer comfort, Eppendorf) at 37°C,. Dissociated samples were pipetted through a 20 µm nylon mesh Multi Screen® (Millipore). Cells were fixed in Fix and Perm solution® medium A (Life Technologies) for 15 min at room temperature (RT). Cells were incubated with primary antibodies (Supplemental Table 3) in 1% bovine serum albumin (BSA) with 0.2% Triton X-100 in PBS for 30 min. After that cells were washed with blocking buffer 1% BSA in PBS, and incubated with a 1:500 dilution of Alexa Fluor 647 conjugated goat anti-mouse secondary antibody (Life Technologies), Alexa Fluor 488 conjugated goat anti-rabbit secondary antibody (Life Technologies) for 20 min in blocking buffer. Cells were washed with blocking buffer and analysed on a flow cytometer (GUAVA easy Cyte 8HT, Millipore) using standard filter sets for secondary antibodies and green fluorescence protein (GFP) of the HES3 $^{NKX2\text{-}5eGFP/w}$ cell line. The GFP expression of HES3

NKX2-5eGFP/w cells from single EB experiment were live analysed and did not require fixation and staining. Analyses were performed with Flowjo®.

Immunohistochemistry

Cultured cells were fixed with 4% paraformaldehyde and blocked for 1 h in 3% BSA/PBS. Primary antibodies (Table S3) were incubated overnight at 4 °C in 0.2% Triton X-100, 3% BSA/PBS. Thereafter, Alexa Fluor 488, 594 and 647 conjugated goat anti-mouse/rabbit secondary antibodies (Life Technologies) were incubated for 2 h at 1:500 dilution in 3% BSA/PBS. Cell imaging was performed with a Nikon eclipse *Ti*-E inverted fluorescence microscope. Pseudo colours were added for a better visualization of the cyto -antibody stains with Image J®.

Metabolic MTT assay

MTT (5 mg/ml) solution (Sigma) was added to dissociate 1.5×10^4 cells/well and differentiating EBs in 96-wells and incubated at 37 °C in a humidified atmosphere of 5% CO2 for 3 h, after which the MTT desorb solution (acidified isopropanol) was added to each well. The plate was shaken for 15 min to dissolve the formazan. The absorbance was measured at 550–570 nm in an Infinite® M200 (Tecan) microplate reader.

Metabolite analyses

Glucose, glutamine, lactate, and ammonia concentrations were analysed using Bioprofile 100 plus (NOVA). pH was measured with a pH meter (Camlab). Methods for analyses and calculating specific metabolites consumption / production rates are described previously by (Chen et al., 2010).

Cell Cycle analyses

Cell cycle analyses were performed by using NucleoCounter NC-3000 (Chemometec) according to the manufacturer's handbook. Briefly: Cells and EBs were dissociated with TrypLE™ Express (Life Technologies) and fixed with 70% ethanol in PBS for 2 h at 2°C followed by incubation of 1 µg/ml DAPI (Sigma), 0.1% Trition-X (Sigma) in PBS for 5 min at 37 °C. DAPI count, size and intensity staining was measured with a NucleoCounter NC-3000 (Chemometec). Cell cycle plots were analysed with FlexiCyte™ software (Chemometec) to calculate cell cycle states in percentage of sub-G1, G1-, S-, G2/M-phase.

Cell Cycle arrest/synchronization

hPSCS were arrested at the G1/S phase by adding 2 mM Thymidine (Sigma) for 18 h in culture media under cell culture condition. G2 arrest was induced by adding 10ng/ml Nocodazole (Sigma) for 12h in culture media under cell culture condition. After the incubation cells were washed with PBS prior further culture or differentiation.

Cell Count

Cell number and cell viability was determined by the nuclei count method with DAPI and Acridine Orange using NucleoCounter NC-3000 (Chemometec) according to the manufacturer's handbook.

Cell lyses and protein quantification assay

Cytosol and nuclear protein fractions were collected and lysed with NE-PER Nuclear and Cytoplasmic Protein Extraction Reagents (Life Technology) with addition of Halt Protease & Phosphatase Inhibitor cocktail (Life Technology) and 1 mM PMSF according to the manufacturer's protocol. Whole protein was extracted with lysis buffer CST (Cell Signaling Technology) containing 1mM phenylmethylsulfonyl fluoride (PMSF) (Sigma) and 1 x Halt Protease & Phosphatase Inhibitor cocktail (Life Technology). Cell lysis solutions were incubated for 15min at 4°C with periodically shaking followed by 10 min spin down at 16000rpm in a microcentrifuge. The supernatant was collected for protein quantification, capillary western blot and stored at -80°C. The protein content of cell lysis solutions and albumin protein standards (Life Technology) were analysed with a colorimetic DC Protein Assay (Bio-rad) according to the manufacture protocol. Light absorbance was measured against a wavelength of 750nm with an Infinite® M200 (Tecan) microplate reader. Protein mass (mg/ml) of the lysis solution was calculated from the albumin protein standards curve.

Capillary western blot and protein antibody Immuno detection, quantification and statistical analyses

Protein identification and quantification was carried out using a Simple Western system (Peggy Sue™, Protein Simple, R&D), a fully automated western capillary based protein separation and detection system (Harris, 2015). All materials with the exception of the primary antibodies were purchased from Protein Simple. The manufactures protocol for size separation and machine settings for size separation (12-230kD) were applied (www.proteinsimple.com). Briefly: Proteins lysates with 1 mg/ml protein content were denaturised with DTT and SDS at 95°C. Samples were size separated through a gel matrix that allow protein identification between 12-230kD. The separated proteins were immobilized to the capillary wall by photo-activation. Followed by blocking, primary antibody (Supplementary Table 3) and secondary horseradish peroxidase conjugated antibody incubation. Protein detection was initiated by induction of Luminol/Peroxidase substrate. The resulting chemiluminescent was measured with a photosensitive camera over a 15 min incubation time. The protein band identification and the quantification of the chemiluminescence were performed with Compass™ software (Proteinsimple, R&D) according to the manufactures protocol. Chemiluminescence values were transformed into western bot image data and line/bar charts with Compass™ software and Excel®. Protein samples were grouped from individual EB and monolayer experiments. A minimum of 48 EBs were used to include a wide range of experimental variations. Protein samples were prepared with up to 3 biological replicates for capillary electrophoresis and antibody detection. A maximum of 12 experimental sample conditions were run simultaneously in individual capillaries. 2-4 Antibodies were detected per sample condition in multiplex analyses. Up to 8 runs per biological sample were performed. Differences between the capillaries and subsequent runs were monitored by an internal system controls and loading controls such as GAPDH, GSK3b, lamin A/C and actin. Relative expression values were obtained by normalizing against loading controls and internal standard across all 8 columns. The protein quantification standard deviation was a found to be 10-25% across all capillaries.

Quantitative RT-PCR

Total RNAwas isolated using the RNeasy Mini Kit (Qiagen) from EBs following the supplier's protocol. Reverse transcription was carried out with 1 µg total RNA using SuperScript III (Invitrogen). Real-time PCR was performed applying a standard two-step amplification protocol on an ABI 7500 system (Applied Biosystem) to detect mRNA expression (primer sequences are, Dkk-1F: 5′ ACCCAGGCTCTGCAGTCA 3′, Dkk-1R: 5′ CCTGCAGGCGAGACAGAT 3′; MESP-1F: 5′ GACGTGCTGGCTCTGTTG 3′, MESP-1R: 5′ TGTCACTTGGGCTCCTCAG 3′, T-bra F: 5′ AATTTGGTCCAGCCTTGGAAT 3′, T-bra R: 5′ CGTTGCTCACAGACCACAG 3′). Relative expression values were obtained by normalizing Cτ values of the tested genes to the Cτ values of the house keeping gene GAPDH using the ΔΔCτ method (Schmittgen and Livak, 2008).

Statistical Analyses

All experiments including EB area, GFP area/percentage, flow cytometry, immunostaining quantification, cell cycle, qPCR, cell numbers and metabolic data were performed at least 3 times. The number of experiments is indicated at the figure caption as n=experiments in independent experiments. All data are expressed as the mean ±SEM using the statistical software GraphPad Prism®, version 4.1. Comparisons of two data sets were statistically analysed with Student's t-test. Multiple comparisons between more than three groups were performed using analysis of variance with one-way Anova multiple comparison tests. A p value is shown when the difference between compared groups is significant. The statistical significance was indicated by *= ($p < 0.05$). The capillary western blot data provides information from a single experiment. Each experiment was analysed individually and statistics were performed from biological replicates and technical replicates system as described earlier. Key experiments leading to general claims were performed with at least 2 additional cell lines and/or with 2 alternative methods, which were included in the supplemental figures.

Supplemental References

Chen, X., Chen, A., Woo, T.L., Choo, A.B.., Reuveny, S., Oh, S.K.., 2010. Investigations into the metabolism of 2D colony and suspended microcarrier cultures of human embryonic stem cells in serum free media. Stem Cells Dev. 19.

Harris, V.M., 2015. Protein Detection by Simple WesternTM Analysis, in: Kurien, B.T., Scofield, R.H. (Eds.), Western Blotting: Methods and Protocols. Springer New York, New York, NY, pp. 465–468. doi:10.1007/978-1-4939-2694-7_47

Lyons, I., Parsons, L.M., Hartley, L., Li, R., Andrews, J.E., Robb, L., Harvey, R.P., 1995. Myogenic and morphogenetic defects in the heart tubes of murine embryos lacking the homeo box gene Nkx2-5. Genes Dev. 9, 1654–1666. doi:10.1101/gad.9.13.1654

Ng, E.S., Davis, R.P., Azzola, L., Stanley, E.G., Elefanty, A.G., 2005. Brief report Forced aggregation of defined numbers of human embryonic stem cells into embryoid bodies fosters robust , reproducible hematopoietic differentiation. Differentiation 106, 1601–1603. doi:10.1182/blood-2005-03-0987.Supported

Schmittgen, T., Livak, K., 2008. Analyzing real-time PCR data by the comparative C(T) method. Nat. Protoc. 3, 1101–8.

Ting, S., Chen, A., Reuveny, S., Oh, S., 2014. An intermittent rocking platform for integrated expansion and differentiation of human pluripotent stem cells to cardiomyocytes in suspended microcarrier cultures. Stem Cell Res. 13, 202–213. doi:10.1016/j.scr.2014.06.002

Ting, S., Lecina, M., Chan, Y.-C., Tse, H.F., Reuveny, S., Oh, S.K., 2013. Nutrient supplemented serum-free medium increases cardiomyogenesis efficiency of human pluripotent stem cells. World J. Stem Cells 5, 86–97. doi:10.4252/wjsc.v5.i3.86

Supplemental Figure S1

A

Images HES3 (Day 0)	Culture Confluence	Cell Number	viability	sub-G1	G1	S	G2/M	pH	qGlucose (g/1e^{6}cells/Day)	qGlutamin (mmol/1e^{6}cells/Day)	qLactate (g/1e^{6}cells/Day)	Yield (Lac/Glc)
a	40%	1.62E+06	90%	6%	51%	21%	21%	7.07	0.56	0.46	1.00	1.78
b	60%	2.94E+06	93%	5%	58%	17%	19%	6.96	0.40	0.23	0.62	1.54
c	80%	3.06E+06	91%	3%	59%	17%	19%	6.88	0.42	0.20	0.65	1.55
d	100%	4.06E+06	92%	4%	59%	19%	16%	6.88	0.29	0.12	0.48	1.64
Medium	mTeSR							7.2	2.5	3	0	

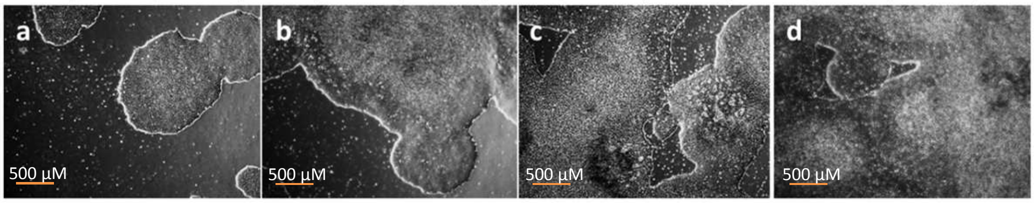

Images after 24h induction (10µM CHIR99021)	Culture Confluence	Cell Number	viability	sub-G1	G1	S	G2/M	pH	qGlucose (g/1e^{6}cells/Day)	qGlutamin (mmol/1e^{6}cells/Day)	qLactate (g/1e^{6}cells/Day)	Yield (Lac/Glc)
e	50%	2.66E+06	84%	3%	24%	19%	53%	8.06	0.36	0.08	0.47	1.28
f	70%	3.64E+06	86%	4%	19%	28%	48%	7.97	0.28	0.04	0.39	1.39
g	75%	2.96E+06	83%	6%	18%	28%	48%	7.99	0.35	0.06	0.50	1.41
h	70%	2.06E+06	79%	6%	18%	18%	56%	7.95	0.51	0.05	0.70	1.37
Medium	bSFS+10µM CHIR99021							8.2	4.5	1	0	

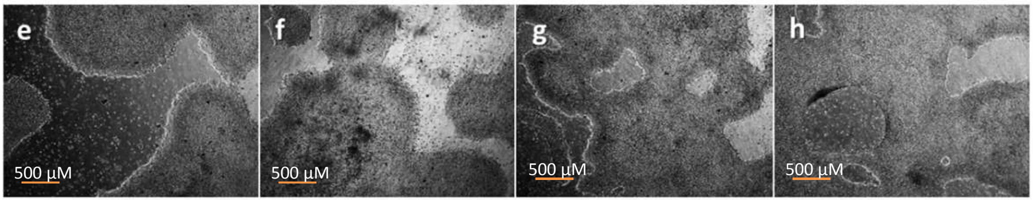

B

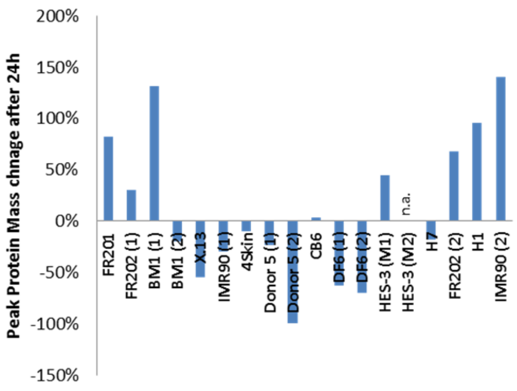

C

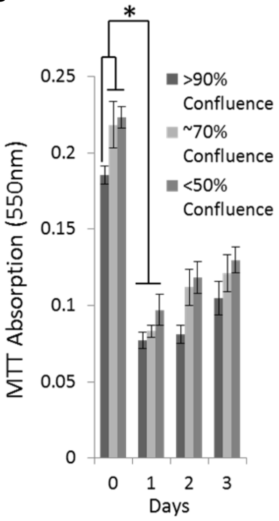

D

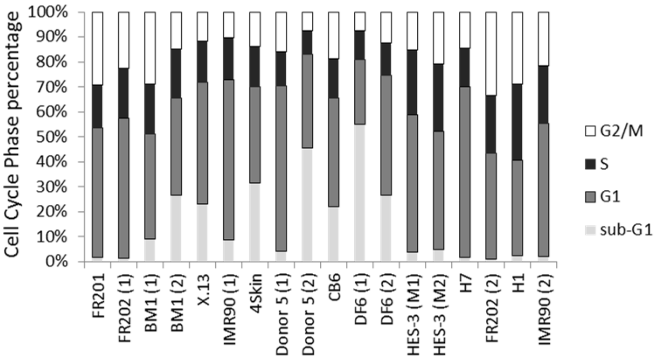

Supplemental Figure S1

Supplemental Figure S1: (A) Cell Cycle, Cell number, viability, pH and metabolite (Glucose, Glutamine and Lactate) measurements and microscope phase contrast images of 40-100% confluent HES3 cells in monolayer culture on day 0 with mTeSR^{TM}1 and after 1 day of treatment with 10µM CHIR in bSFS medium. (B) Maximal change of protein mass percentage after 24h treatment with 4-12 µM CHIR (C) Cell metabolism and growth (MTT absorbance) kinetics of EBs generated from passage 10 with 50%, 70% and 90% culture confluency induced with 6 µM CHIR for 24 h (n=3 ind. ex.). (D) Graphical illustration of cell cycle profiles of 11 hPSC lines.

Supplemental Figure S2

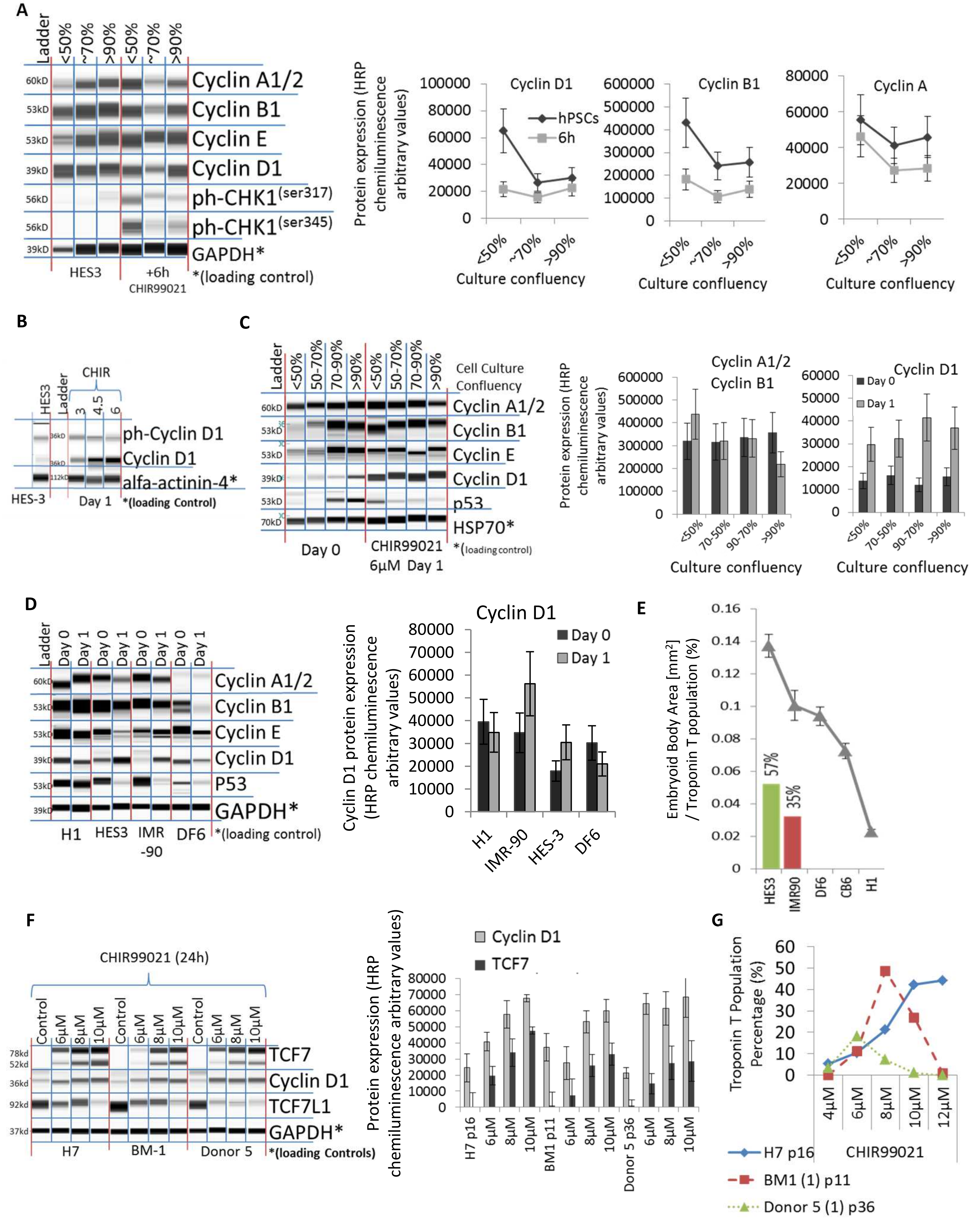

Supplemental Figure S2

Supplemental Figure S2: (A) Whole cell protein blot and quantitative analyses of hPSCs cultured at 50%, 70% and 90% culture confluency after treatment with 12 µM CHIR. (B) Whole cell protein blot of HES3 EB cell cultures after CHIR induction. Analyses showed dose dependency of Cyclin D1 protein expression. (C) Whole cell protein blot and quantitative analyses of HES3 cells cultured at <50%, 50-70%, 70-90%, >90% cell culture confluency and HES3 EBs after CHIR induction. (D) Whole cell protein blot and quantitative analyses of hPSCs and hPSC EBs after CHIR induction (6 µM H1, HES-3, IMR-90 and 3 µM DF6). (E) EB area size of 5 cell lines after 24 h treatment with CHIR (6 µM H1, HES-3, IMR-90 and 3 µM DF6) and their Troponin T population expression (%) expression on day 14. (F) Whole cell protein blot and quantitative analyses of H7, BM1 and Donor 5 cells after CHIR induction. Analyses showed dose dependency of TCF7 and Cyclin D1 protein expression levels. (G) Troponin T expression on day 14 of 3 H7, BM1 and Donor 5 cells after CHIR induction for 24h.

Supplemental Figure S3

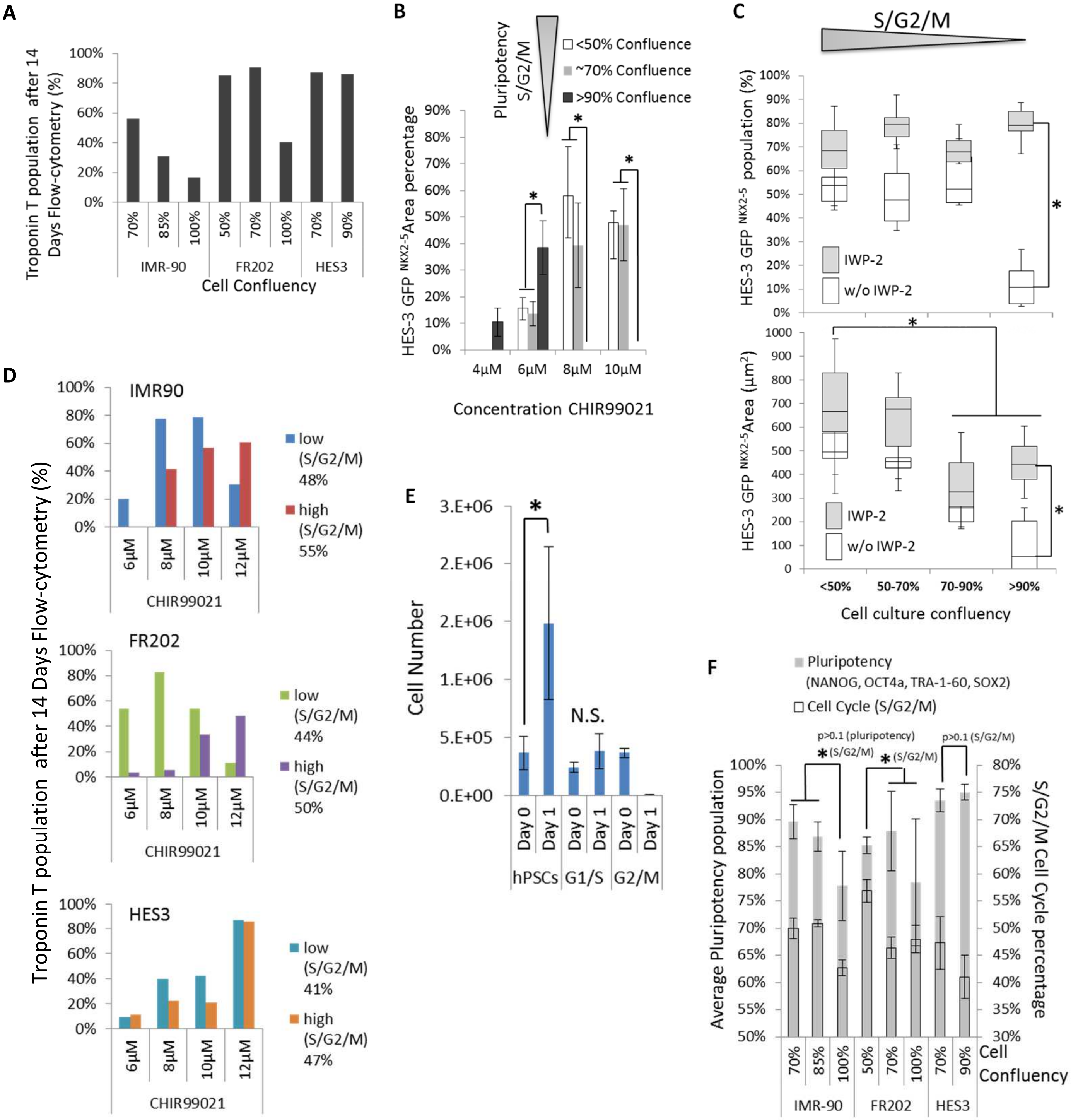

Supplemental Figure S3: (A) Troponin T cell population (%) of monolayer hPSC lines (IMR90, FR202 and HES3) on day 14 cultured at 50%-100% culture confluency and differentiated with 12 μM CHIR induction for 24 h (B) EB GFP $^{NKX2-5}$ expression on day 11 of HES3 cells cultured at <50%, 70%, 90% cell culture confluency of low to high S/G2/M cell cycle profiles and differentiated with 4-10 μM CHIR induction for 24h (n=4). (C) EB GFP $^{NKX2-5}$ flow cytometry population and total GFP $^{NKX2-5}$ positive EB area of HES3 cells cultured at <50%, 50-70%, 70-90%, >90% cell culture confluency and differentiated with 6 μM CHIR induction for 24 h and 5 μM IWP-2 induction on day 3 (n=8). (D) Troponin T population (%) on day 14 of monolayer hPSC lines (IMR90, FR202 and HES3) cultured at low 50-70% to high >90% cell culture confluency with a 6% difference in S/G2/M cell cycle profile and differentiated with CHIR induction for 24h. (E) Average cell number of pooled HES3, FR202 and IMR90 cell lines after cell cycle arrest at G1/S and G2 and 12 μM CHIR induction (F) Average pluripotency flow cytometry population (%) of NANOG, OCT4a, TRA-1-60 and SOX2 and the percentage of the S/G2/M cell cycle profile of IMR90, FR202 and HES3 cells cultured at variable cell densities (n=3).

Supplemental Figure S4

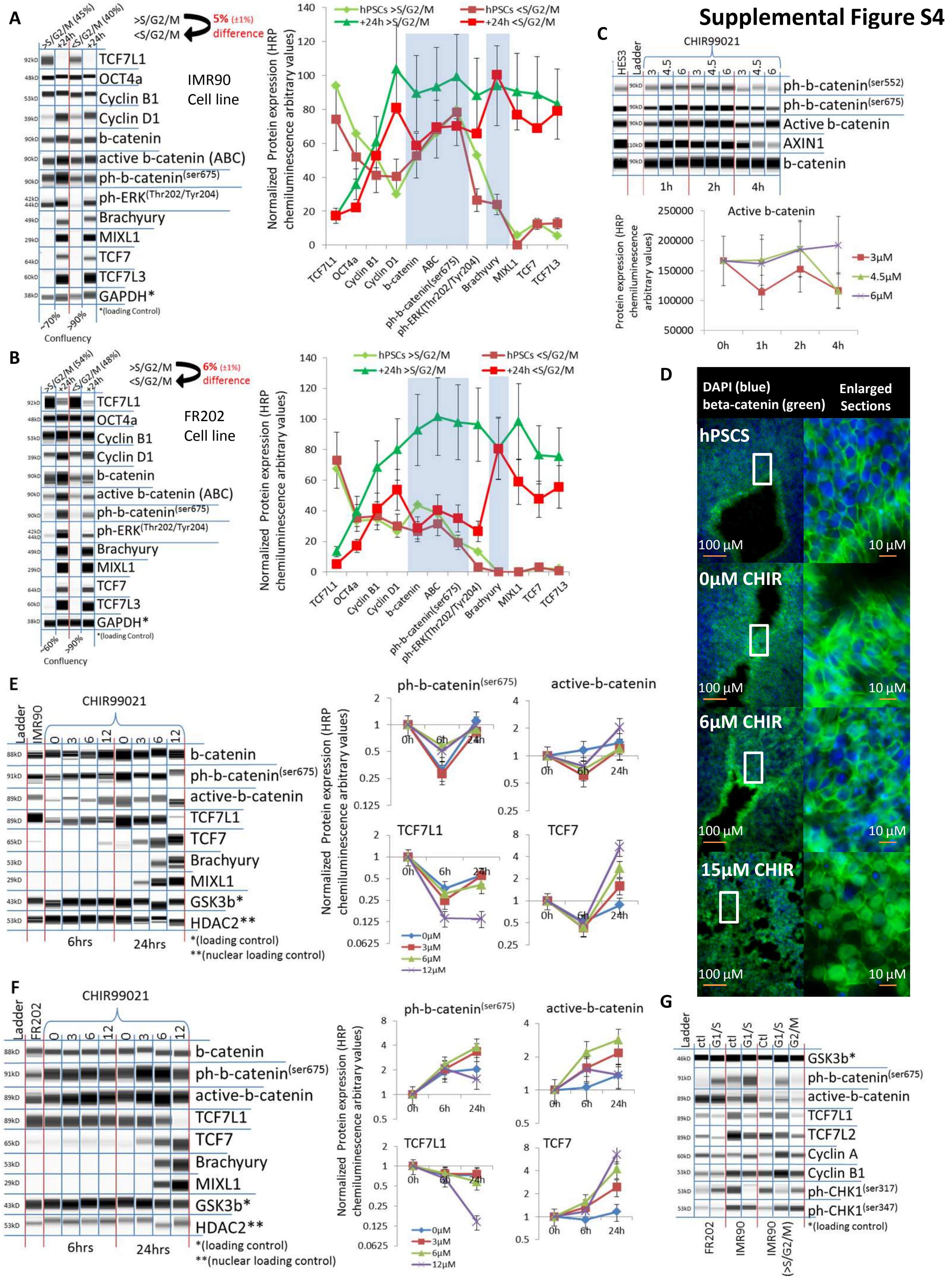

Supplemental Figure S4

Supplemental Figure S4: (A) Whole cell protein blot and quantitative analyses of IMR90 cells cultured at >90% culture confluency with a low S/G2/M (40%) cycle profile and ~70% culture confluency cell with a high S/G2/M (45%) cycle profile after 10 μM CHIR induction. High culture density with a low S/G2/M cell cycle poorly expressed catenin proteins. T-Brachyury was not affected by cell culture density and S/G2/M cell cycle profile differences. (B) Whole cell protein blot and quantitative analyses of FR202 cells cultured at >90% culture confluency with a low S/G2/M S/G2/M (48%) cycle profile , >90% culture confluency and ~60% culture confluency cell with a high S/G2/M (54%) cycle profile after 12 μM CHIR induction. High culture density with a low S/G2/M cell cycle poorly expressed catenin proteins. T-Brachyury was not affected by cell culture density and S/G2/M cell cycle profile differences. (C) Whole cell protein blot and quantitative analyses of HES3 and HES3 EBs after CHIR induction. (D) Fluorescence microscopy of immuno-cytochemistry stained FR202 cells against active beta-catenin (green) and DAPI (blue) after 24 h treatment with CHIR. DAPI co-staining with beta-catenin indicates nuclear translocation. (E) Nuclear fraction protein blot and quantitative analyses of IMR90 cells after CHIR induction. Analyses shows dose dependency of TCF proteins. (F) Nuclear fraction protein blot and quantitative analyses of FR202 cells after CHIR induction. Analyses shows dose dependency of TCF and catenin proteins. (G) Whole cell protein blot of Wnt signalling, and Cyclin proteins of the FR202 (S/G2/M=46%) and IMR90 (S/G2/M=43%) and (S/G2/M=51%) cell lines after cell cycle arrest.

Supplemental Figure S5

A

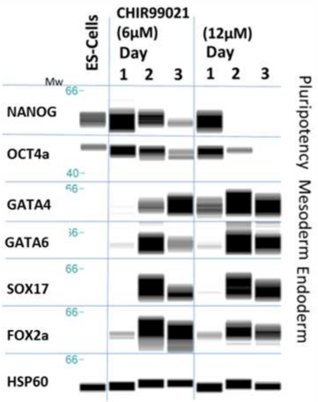

B

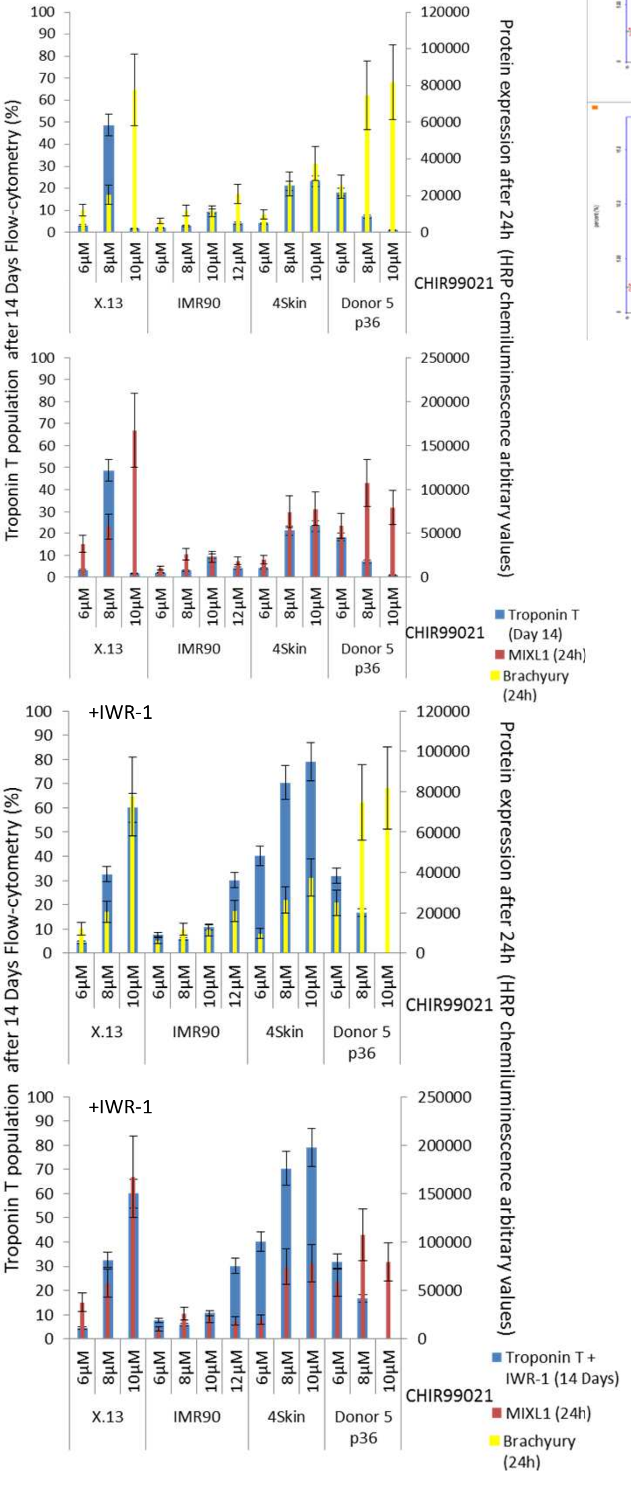

C

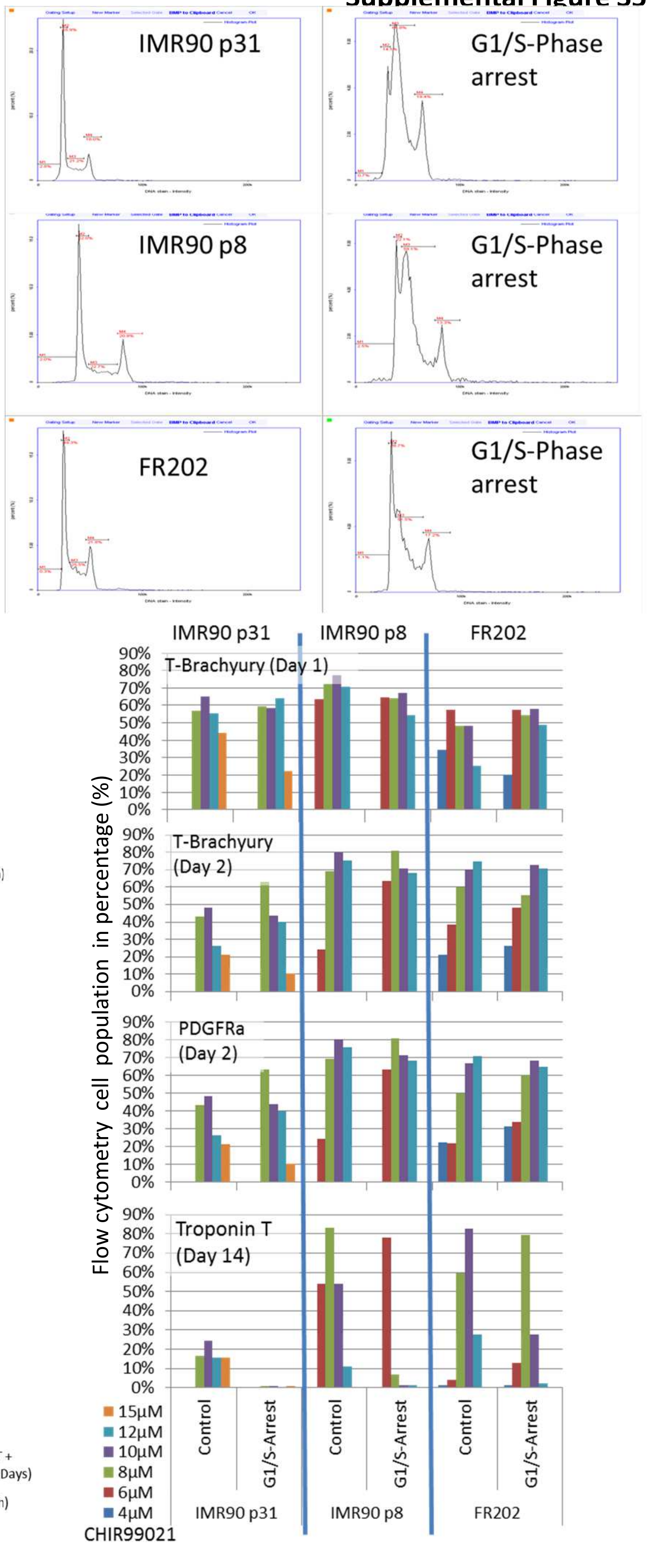

Supplemental Figure S5

Supplemental Figure S5: (A) Whole cell protein blot of pluripotency signalling proteins (NANOG, OCT4), and Endo-Mesoderm markers (GATA4/6, SOX17, FOX2a) expression of microcarrier attached IMR90 aggregates treated for 24h with CHIR. (B) HRP chemiluminescence expression analyses of T-Brachyury and MIXL1 proteins of X.13, IMR90, 4Skin and Donor 5 cells after CHIR induction, and flow cytometry population (%) of Troponin expressing cells on day 14. (C) Cell cycle profiles of IMR90 p31 and p8 and FR202 with and without G1/S arrest. Flow cytometry population (%) of T-Brachyury on day 1-2, PDGFRa on day 2 and Troponin T on day 14 of IMR90 p31, p8 and FR202 cells after G1/S cell cycle arrested and treatment with 4-15 μM CHIR for 24 h.

Supplemental Figure S6

A

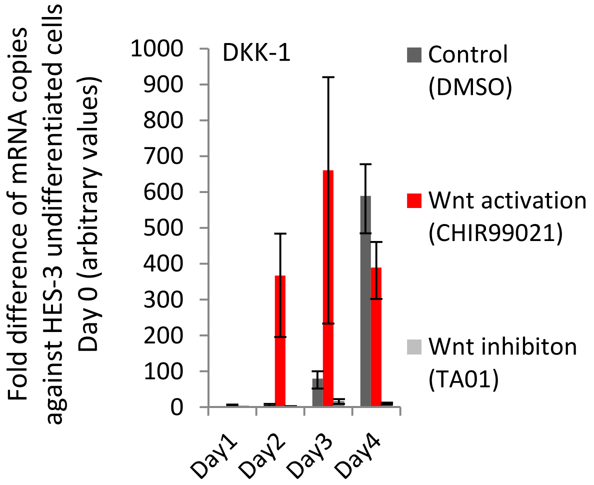

B

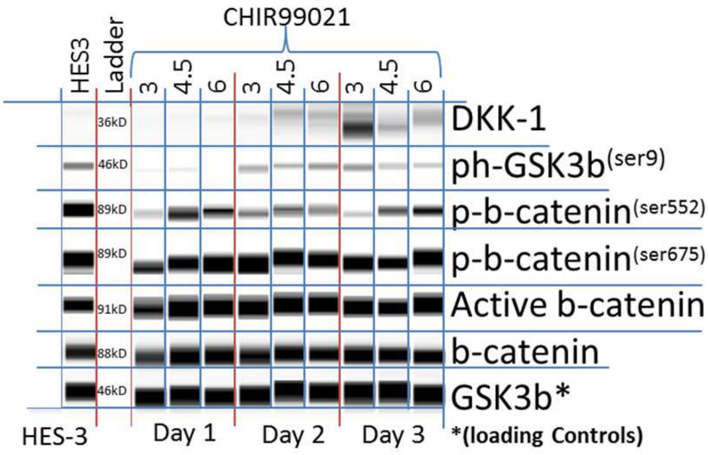

Supplemental Figure S6: (A) qPCR analyses of Wnt inhibitor DKK-1 with 5µM TA-01 (Wnt inhibition) and 3 µM CHIR (h). Measurements are compared to HES3 and displayed as fold increase/decrease over HES3 control, where 1 is equal to the HES3 control (n=3) (B) Whole cell protein blot of DKK-1/GSK3b/b-catenin signalling proteins of HES3 cells and EBs. b-catenin$^{(ser522,\ ser675)}$ levels declined after treatment with 3 µM CHIR for 24 h. All b-catenin levels were not significantly increased after CHIR induction. The increase of DKK-1 and ph-GSK on day 2/3 indicate Wnt inhibition.

Supplemental Figure S7

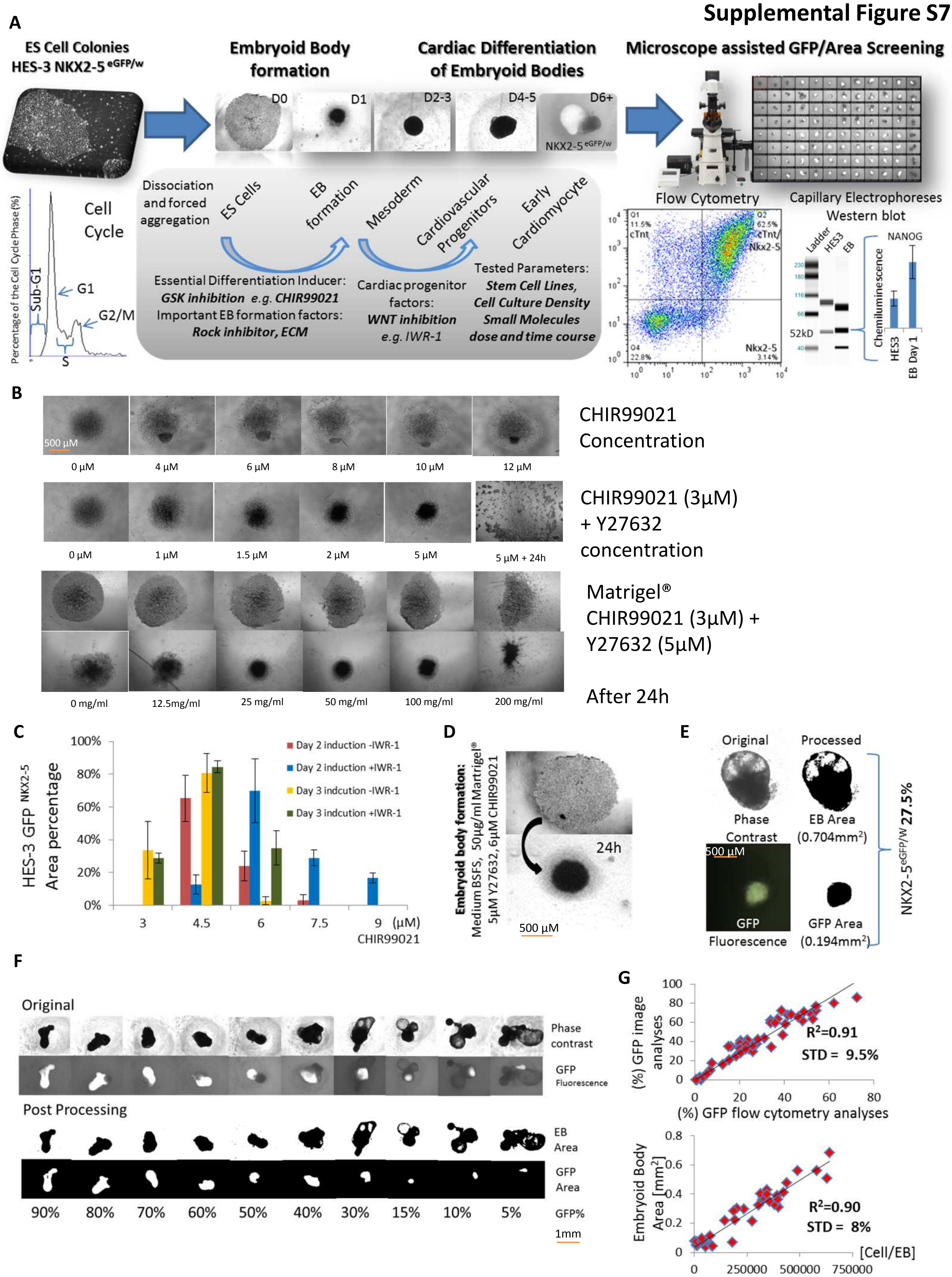

Supplemental Figure S7

Supplemental Figure S7: (A) Workflow: HES3 $^{NKX2\text{-}5eGFP}$ reporter cell line cultures were analysed for cell cycle, cell number, cell culture density and pluripotency. Thereafter 15000 cells harvested from these cultures were seeded in 96-wells to form EB by forced aggregation with Rock inhibitor and ECM supplementation and in presence of CHIR inducer which initiate differentiation via Wnt regulation towards mesoderm and early cardiomyocytes. EB analyses included size (measurement of area), differentiation (measurement of GFP area, white area), flow cytometry, cell count and cell protein analyses with capillary electrophoresis western blot. (B) Microscope phase contrast images of forced aggregated EBs after treatment with CHIR and Y27632 for 24 h (top 2 image rows). Microscope phase contrast images of forced aggregated HES3 cells and EBs after 24 h with Geltrex® (bottom 2 image rows). (C) Percentage of GFP $^{NKX2\text{-}5}$ area of HES3 EBs on day 10 days after treatment with 3-9 µM CHIR for 24h and 2.5µM IWR-1 induction on day 2/3. (D) Example of EB and GFP area analyses with ImageJ® for GFP area differentiation percentage. (E) Phase contrast image of 15000 forced aggregated HES3 cells and after EB formation with standard method parameters of 50mg/ml Matrigel®, 6 µM CHIR and 5 µM Y27632. (F) Original: microscope phase contrast (top) of EB and fluorescence images of GFP expression (white). Post processing: EB and GFP area analyses with Image J for GFP area percentage. (G) Correlation between EB differentiation efficiency measured by flow cytometry and GFP area percentage measured by imaging and calculating the GFP/EB area (Top) (n=51, >204 EBs). Correlation between EB size evaluation by measurement of area by imaging and cell count of dissociated EBs (Bottom) (n=44, >176 EBs).

TABLE S1

Cardiac differentiation optimasation:

BSFS* Media, 5µM Y27632, 50µg/ml Matrigel, 12500 hESCs/Well (120µl Volume)

Media Change — Days 0, 1, 2, 3-5, 6 (Day 1–2: CHIR99021 1.5µM; Day 3-5: IWR-1 2.5µM; Day 6: Media Change to BSFS*)

Days 0 (Media Change)	Analyses Microscopy Day 10: EB Area (mm^2)	STD	GFP Area (mm^2)	STD	GFP Area (%)	STD	Flow Cytometry Analyses Day 10: GFP Flow (%)	STD	Cell Count Cells/µl	STD	GFP Yield Cells/µl
CHIR99021 (1.5µM)	0	0	0	0	0%	0%					
	0	0	0	0	0%	0%					
	0	0	0	0	0%	0%					
	0	0	0	0	0%	0%					
	0.120	0.001	0	0	0%	0%					
	0.104	0.005	0	0	0%	0%					
	0	0	0	0	0%	0%					
	0	0	0	0	0%	0%					
CHIR99021 3µM	0.149	0.042	0	0	0%	0%					
	0.128	0.032	0	0	0%	0%					
	0	0	0	0	0%	0%					
	0	0	0	0	0%	0%					
	0.275	0.123	0.057	0.043	21%	16%	34%	17%	87	4	29
	0.161	0.017	0	0	0%	0%					
	0.179	0.066	0.046	0.011	27%	4%	29%	3%	98	37	28
	0.113	0.014	0	0	0%	0%					
	**0.392**	0.069	0.020	0.02	1%	3%	11%	13%	59	46	6
	0.281	0.182	**0.157**	0.0735	**60%**	10%	**70%**	9%	88	48	**62**
	0.194	0.042	0.114	0.0370	**58%**	11%	59%	8%	55	12	32
	0.310	0.085	0.119	0.0748	36%	14%	51%	15%	**126**	46	**65**
CHIR99021 4.5µM	0.289	0.012	0.031	0.001	9%	1%	9%	2%	166	12	15
	0.280	0.001	0.029	0.001	10%	0%	9%	3%	173	7	15
	0.114	0.007	0	0	0%	0%					
	0	0	0	0	0%	0%					
	0.250	0.088	0.163	0.034	67%	9%	**81%**	12%	**126**	24	**102**
	0.174	0.065	0.065	0.037	36%	7%	65%	14%	104	11	68
	0.269	0.046	**0.282**	0.006	**93%**	9%	**84%**	4%	75	11	64
	0.210	0.041	0.020	0.010	9%	4%	13%	6%	91	17	11
	0.291	0.100	0	0	0%	0%					
	**0.623**	0.081	0	0	0%	0%					
	0.191	0.006	0	0	0%	0%					
	0.256	0.027	0.105	0.032	41%	15%	26%	1%	83	7	22
CHIR99021 6µM	0.402	0.053	0.201	0.054	50%	10%	32%	5%	209	44	67
	0.466	0.040	0.246	0.041	53%	10%	33%	5%	202	39	68
	0.456	0.074	0.236	0.019	53%	11%	50%	5%	**232**	58	**116**
	0.203	0.080	0	0	0%	0%					
	0.295	0.019	0.013	0.013	1%	2%	3%	2%	117	9	3
	0.391	0.059	0.154	0.0747	38%	15%	24%	9%	172	14	41
	0.269	0.046	0.119	0.0286	44%	5%	35%	6%	104	16	36
	0.339	0.049	**0.265**	0.0075	**79%**	10%	**70%**	14%	122	18	85
	0.335	0.158	0	0	0%	0%					
	**0.981**	0.228	0	0	0%	0%					
	0.126	0.014	0	0	0%	0%					
	0.146	0.012	0	0	0%	0%					
CHIR99021 7.5µM	0.253	0.072	0	0	0%	0%					
	0.276	0.048	0	0	0%	0%					
	0.430	0.017	0.221	0.019	51%	6%	23%	4%	149	13	34
	0.534	0.086	0.137	0.070	25%	11%	27%	16%	**286**	58	**76**
	0.448	0.044	0	0	0%	0%					
	0.510	0.102	0.024	0.024	5%	10%	3%	4%	152	41	4
	0.207	0.011	0	0	0%	0%					
	0.405	0.022	**0.260**	0.002	**64%**	3%	**29%**	5%	59	13	17
	0.113	0.009	0	0	0%	0%					
	**0.913**	0.135	0	0	0%	0%					
	0	0	0	0	0%	0%					
	0.104	0.006	0	0	0%	0%					
CHIR99021 9µM	0.301	0.055	0	0	0%	0%					
	0.274	0.045	0	0	0%	0%					
	0.315	0.088	0.037	0.050	10%	12%	6%	3%	211	68	13
	0.575	0.072	**0.223**	0.038	39%	6%	**30%**	7%	**244**	50	**73**
	0.550	0.049	0	0	0%	0%					
	0.232	0.144	0	0	0%	0%					
	0.285	0.088	0	0	0%	0%					
	0.419	0.079	0.183	0.040	**44%**	9%	17%	3%	116	35	19
	0.111	0.003	0	0	0%	0%					
	**0.806**	0.206	0	0	0%	0%					
	0	0	0	0	0%	0%					
	0	0	0	0	0%	0%					

TABLE S2

hPSCS analyses and cardiac differentiation results with CHIR99021

Cell lines		FR201	FR202	BM1 (1)	BM1 (2)	X.13	IMR-90 (1)	4Skin	Donor 5 (1)	Donor 5 (2)	CB6	DF6 (1)	DF6 (2)	HES-3 (M1)	HES-3 (M2)	H7	FR202 (I)	H1 (I)	IMR-90 (2)
Passage Number		P10-4	P10-4	p11	p13	p11-8	p5-32-8-7	p12-13(F)-10	batch 1 p36	batch 2 p35	p18	batch 1 p28	batch 2 p29	p2-14-7	p2-14-7	p17	p10-5-7	p14-19-9	p5-32-8-8
**Day 0**																			
Pluripotency	Oct4a	83%	80%	90%	90%	76%	77%	88%	90%	50%	61%	48%	38%	98%	98%	87%	93%	97%	88%
	Nanog	83%	83%	90%	88%	62%	76%	89%	81%	36%	55%	48%	30%	97%	98%	77%	92%	96%	89%
Cell Confluency		75%	85%	95%	95%	98%	86%	83%	88%	n.a.	n.a.	76%	80%	96%	62%	85%	70%	70%	70%
Metabolism	sub-G1	2%	2%	9%	26%	23%	9%	31%	4%	51%	22%	54%	27%	4%	5%	2%	1%	2%	2%
	G1	52%	56%	42%	38%	48%	64%	38%	67%	42%	43%	25%	47%	55%	47%	66%	41%	38%	52%
	S	17%	20%	20%	19%	16%	17%	16%	14%	11%	16%	12%	13%	26%	27%	15%	23%	30%	23%
	G2/M	29%	22%	29%	15%	12%	10%	14%	16%	8%	19%	7%	12%	15%	21%	14%	33%	29%	21%
Cell Cycle (S+G2/M)		**47%**	**42%**	**49%**	**34%**	**28%**	**27%**	**30%**	**30%**	**19%**	**34%**	**19%**	**25%**	**41%**	**47%**	**29%**	**55%**	**59%**	**44%**
DC Protein Assay Protein Mass (ug/ml)		6.844203	6.612319	2.914855	7.014493	17.02174	12.9837	9.338768	8.387681	8.282609	7.373188	8.815217	9.865942	13.97826	4.17029	13.12681	2.83	4.03	2.01
Protein expression (Chemilunisecence)																			
	TCF7L1	144665.9	181283	326034	160757.4	128404	228057.6	165814.1	179474	n.a.	180852	199080	n.a.	222527	341141	179379.3	202406.3	n.a.	429887
	TCF7L2	12049.75	9486	37742	33508.27	28838.28	40234.62	18341.51	34508	n.a.	23451	31901.03	n.a.	44533.44	87471	34039.35	25050.81	n.a.	69996.1
	b-catenin	124188.7	n.a.	n.a.	107816.7	90726.97	131823.5	176241.9	n.a.	n.a.	139845.4	133235.6	n.a.	121072.9	n.a.	130641.2	120201	n.a.	119211
**Day 1**	CHIR99021																		
Protein DC Assay (µg/ml)	0µM	n.a.	n.a.	n.a.	n.a.	5.966305	n.a.	n.a.	n.a.	n.a.	n.a.	n.a.	n.a.	10.72105	n.a.	n.a.	n.a.	n.a.	n.a.
	4µM	n.a.	n.a.	n.a.	n.a.	7.515218	8.893478	n.a.	6.376631	<0.2	n.a.	n.a.	1.136232	19.77264	n.a.	7.699275	n.a.	n.a.	n.a.
	6µM	1.576993	2.009964	6.302174	4.113768	6.567391	8.68913	5.726087	5.563044	<0.2	7.588406	3.303261	2.977536	20.19203	n.a.	8.099638	4.748458	5.687044	4.83
	8µM	12.47826	8.605435	6.734783	5.602989	7.717391	9.089131	8.408696	5.929891	<0.2	5.267391	2.080435	1.780435	n.a.	n.a.	8.307971	4.730791	5.982427	4.42
	10µM	n.a.	n.a.	5.254348	3.06413	6.258696	8.432609	5.584239	4.213043	n.a.	0.76558	1.926087	0.513043	n.a.	n.a.	10.75815	3.918116	7.867639	4.04
	12µM	n.a.	n.a.	1.280072	4.0125	n.a.	8.43913	5.205978	n.a.	n.a.	n.a.	0.563768	n.a.	14.47924	n.a.	7.326087	3.228454	n.a.	4.42
Peak Protein Mass Change (%) 24h		**82%**	**30%**	**131%**	**-20%**	**-55%**	**-30%**	**-10%**	**-24%**	**-100%**	**3%**	**-63%**	**-70%**	**44%**		**-18%**	**68%**	**95%**	**140%**
**Day 14**																			
Peak Nkx2-5 expression (%)		13.9	4.95	44.7	48.7	41.8	31	29.2	14.4	cell death	6.41	13.4	2.81	95.5	92.8	59.3	47	73	83
CHIR99021 + IWR-1																			
Peak Nkx2-5 expression (%)		21.4	8.22	74.3	75.8	57.9	32.7	72.5	25.1	cell death	2.15	42.9	cell death	95	n.a.	69.3	69	n.a.	n.a.
Troponin T (%)	4µM	n.a.	n.a.	n.a.	n.a.	1.51	0.661	n.a.	3.46	n.a.	2.1	n.a.	n.a.	n.a.	n.a.	5.23	n.a.	n.a.	n.a.
	6µM	2.68	1.79	11.1	7.29	3.19	2.08	4.02	18.2	n.a.	0.757	14.1	3.52	9.37	11.1	10.4	0.6	2.6	54.1
	8µM	4.08	5.26	48.7	46.2	48.7	2.9	21.2	7.2	n.a.	1.55	10.7	n.a.	39.4	22.5	21.2	41.5	60.3	83.1
	10µM	29.3	17.7	26.9	5.88	1.53	9.54	23.2	1.02	n.a.	7.46	1.23	n.a.	42.5	21.1	42.2	56.6	73.7	54.2
	12µM	*	*	0.81	0.955	n.a.	2.06	10.3	n.a.	n.a.	n.a.	n.a.	n.a.	87.1	86.1	44.2	60.5	n.a.	11.2
Peak Troponin T expression (%)		**29.3**		**48.7**	**46.2**	**48.7**	**9.54**	**23.2**	**18.2**	**0**	**7.46**	**14.1**	**3.52**	**87.1**	**86.1**	**44.2**	**60.5**	**73.7**	**83.1**
CHIR99021 + IWR-1																			
Peak Troponin T expression (%)		39.3	27.5	75.6	71.3	60	17.8	79.1	31.9	cell death	6.36	41.1	cell death	88	n.a.	51	91	n.a.	n.a.

*outlier! Missing date from 12µM experiment; data not included in day 14 calculations

TABLE S2

hPSCS analyses and cardiac differentiation results with CHIR99021

Charts from Table S2

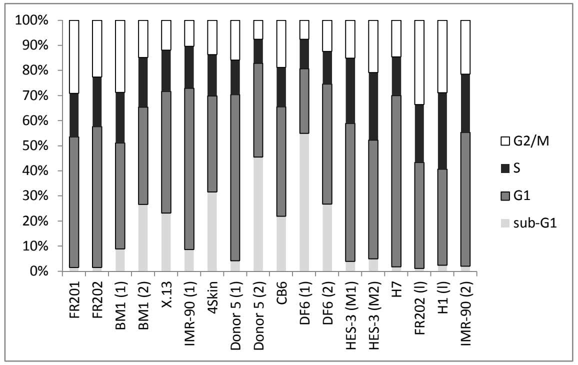

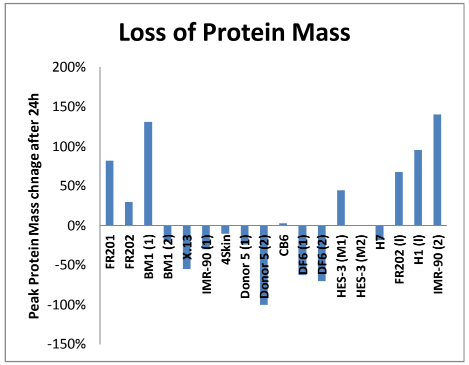

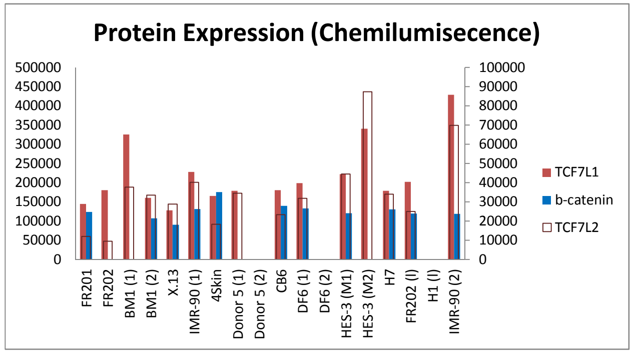

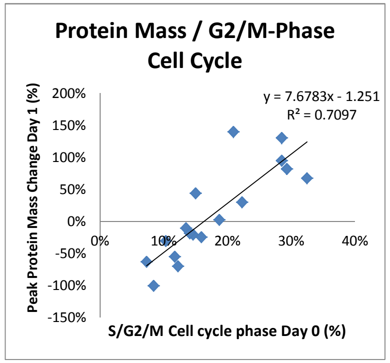

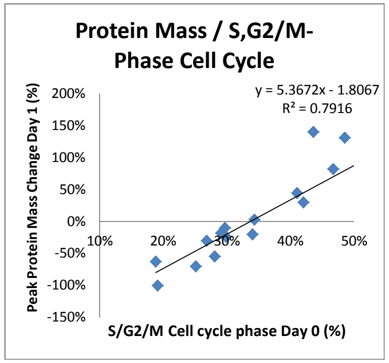

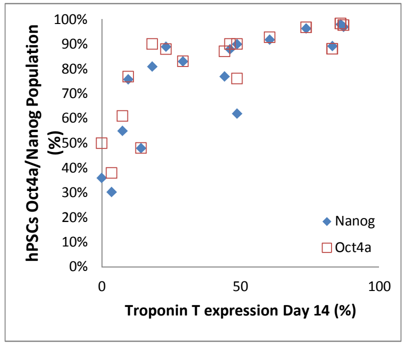

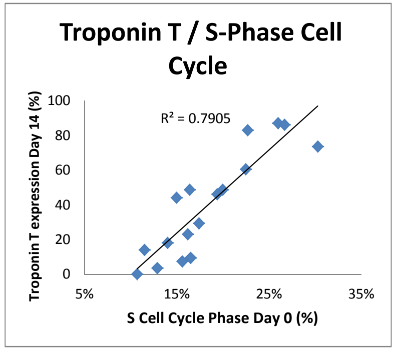

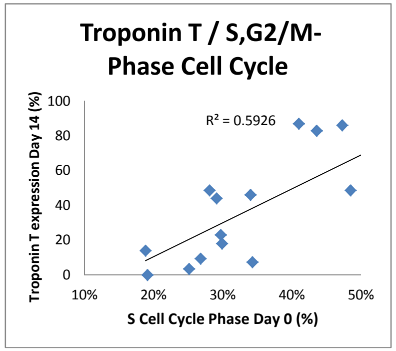

TABLE S3

All Data for CST lyses with a Protein weight of 0.5mg/ml, 12-130KD Protein Simple Kit

Antibody	Company	#	Clone	Iso-tope	2nd Ab	Ex-pected kD	Protein Simple kD	Back-ground	WB IPS/ES	WB Differen-tiation	Cardio-myocytes Flow cytometry	IF
**α-actinin-4**	Santa Cruz	390180	(B-11)	IgG2a	m	105	91-97		1:50	1:50		
**b-actin**	Cell Signaling	4967	pAb	-	r	45	45		1:500	1:500		
**AKT**	Cell Signaling	9272	pAb	-	r	60	62		1:50	1:50		
**Phospho-AKT (ser473)**	Cell Signaling	4060	(D9E)	IgG	r	60	64		1:50	1:50		
**Axin 1**	Cell Signaling	2087	(C76H11)	IgG	r	110	112-117		1:75	1:75		
**b-catenin (IF)**	Cell Signaling	2677	(L54E2)	IgG1	m	-	-	-	-	-		1:200
**b-catenin**	Cell Signaling	2698	(L87A12)	IgG1	m	92	79-82		1:500	1:500		
**b-catenin**	Cell Signaling	8480	(D10A8)	IgG1	r	92	80		1:1500	1:1500		
**Active-b-catenin**	Cell Signaling	8814	(D13A1)	IgG	r	92	82-87		1:1500	1:1500		1:200
**Phospho-b-catenin (ser675)**	Cell Signaling	5176	(D2F1)	IgG	r	92	82-85		1:250	1:250		
**Phospho-b-catenin (ser552)**	Cell Signaling	9566	pAb	-	r	92	81-90		1:75	1:75		
**Ph-b-catenin (ser33/37/thr41)**	Cell Signaling	9561	pAb	-	r	92	90-96		1:20	1:20		
**Phospho-b-catenin (ser45)**	Cell Signaling	9564	pAb	-	r	92	85-89		1:50	1:50		
**b-tubulin**	Cell Signaling	2146	pAb	-	r	55	58		1:400	1:400		
**bFGF**	Cell Signaling	3196	(19A9)	IgG	r	19	26-29		1:25	-		
**Phospho-cdc2 (Tyr15)**	Cell Signaling	4539	(10A11)	IgG	r	34	40		1:25	-		
**Phospho-Chk1 (Ser345)**	Cell Signaling	2348	(133D3)	IgG	r	56	68		1:25	-		
**Cyclin A1/2**	Santa Cruz	751	(H-432)	IgG	r	54	50/59		1:100	-		
**Cyclin B1**	Cell Signaling	4135	(v152)	IgG1	m	55	64		1:100	-		
**Cyclin D1**	Cell Signaling	2978	(92G2)	IgG	r	36	40		1:25	1:25		
**Phospho-Cyclin D1 (Thr286)**	Cell Signaling	3300	(D29B3)	IgG	r	36	40		1:25	1:25		
**Cyclin E**	Santa Cruz	25303	(E-4)	IgG1	m	53	59		1:100	-		
**Dishevelled 2**	Cell Signaling	3224	(30D2)	IgG	r	90-95	105		1:100	1:100		
**DKK-1**	LSBio	ls-b8698	EPR4759		r	36	38-41		1:250	1:250		
**FoxA2/HNF3b**	Cell Signaling	8186	(D56D6)	IgG	r	50	58		1:25	1:100		
**FoxO1**	Cell Signaling	2880	C29H4	IgG	r	78-82	85		1:100	1:100		
**FoxO3a**	Cell Signaling	12829	(D19A7)	IgG	r	82-97	90		1:100	1:100		
**GAPDH**	Santa Cruz	365062	(G-9)	IgG1	m	37	39-41		1:40000	1:40000		
**GAPDH**	Cell Signaling	2118	(14C10)	IgG	r	37	39-41		1:4000	1:4000		
**GATA4**	Cell Signaling	14353	pAB	-	r	55	55-59		1:25	1:25		
**GATA6**	Cell Signaling	5851	(D61E4)	IgG	r	55	58-62		1:25	1:25		
**GSK-3b**	Cell Signaling	12456	(D5C5Z)	IgG	r	46	55		1:1000	1:1000		
**HDAC2**	Cell Signaling	5113	(3F3)	IgG1	m	60	60		1:200	1:200		
**Phospho-GSK-3b (ser9)**	Cell Signaling	9323	(5B3)	IgG	r	46	55		1:50	1:50		
**Phospho-GSK-3b (thr380)**	Cell Signaling	3548	pAb	-	r	46	55		1:25	-		
**HSP60**	Cell Signaling	12165	(D6F1)	IgG	r	60	55-59		1:500	1:500		
**HSP70**	Santa Cruz	32239	(3A3)	IgG	m	70	77		1:1500	1:1500		
**LaminA/C**	Cell Signaling	4777	(4C11)	IgG2a	m	74,63	75		1:25	-		
**LEF1 (TCF7L3)**	Cell Signaling	2230	(C12A5)	IgG	r	25, 58	60		1:25	1:50		
**Phospho-LRP6 (ser1490)**	Cell Signaling	2568	pAb	-	r	180/210	200-210		1:25	1:25		
**LRP6**	Cell Signaling	2560	(C5C7)	IgG	r	180/210	200-210		1:200	1:200		
**MIXL1**	Santa Cruz	390976	(A-8)	IgG	m	25	29		1:25	1:25		
**MEF2c**	Cell Signaling	5030	(D80C1)	IgG	r	50-60	58-62		1:50	1:50		
**MESDC2**	Cell Signaling	2763	pAb	-	r	25/26	36		1:25	1:25		
**NANOG**	Cell Signaling	4903	(D73G4)	IgG	r	42	56		1:50	1:50		
**NKX2.5**	Cell Signaling	8792	(E1Y8H)	IgG	r	30-42	47-49		1:50	1:50	1:300	
**p53**	Santa Cruz	126	(DO-1)	IgG2a	m	53	57		1:20	-		
**Phospho-p53 (Ser15)**	Cell Signaling	9286	(16G8)	IgG1	m	53	57		1:10	-		
**PDGFRa**	Cell Signaling	5241	(D13C6)	IgG	r	-	-	-	-	-	1:200	
**Oct4a**	Cell Signaling	2840	(C30A3)	IgG	r	45	47-49		1:50	1:50		
**SMAD1**	Cell Signaling	6944	(D59D7)	IgG	r	62	64		1:100	1:100		
**Phospho-SMAD1 (ser206)**	Cell Signaling	5753	(D40B7)	IgG	r	62	64		1:25	1:25		
**Slug**	Cell Signaling	9585	(C19G7)	IgG	r	30	44		1:50	1:50		
**Snail**	Cell Signaling	3879	(C15D3)	IgG	r	29	42		1:50	1:50		
**SOX2**	Cell Signaling	3579	D6D9	IgG	r	-	-	-	-	-	1:400	
**SOX17**	Cell Signaling	13863	pAb	-	r	55	54/65		1:25	1:75		
**T-Bra**	Santa Cruz	374321	(A-4)	IgG2b	m	49	52		1:100	1:100		
**T-Bra**	R&D	IC2085G	pAb	IgG	g	49	52	-	1:10	1:10	1:50	
**T-Bra**	Cell Signaling	81694	(D2Z3J)	IgG	r	49	52		1:400	1:400	1:100	
**TCF1 (TCF7)**	Cell Signaling	2203	(C63D9)	IgG	r	48/50	54/66/90		1:25	1:50		1:200
**TCF3 (TCF7L1)**	Cell Signaling	2883	(D15G11)	IgG	r	78	95		1:100	1:100		
**TCF4 (TCF7L2)**	Cell Signaling	2569	(C48H11)	IgG	r	58, 79	63/91		1:50	1:50		
**TRA-1-60**	Cell Signaling	4746	-	IgM	m	-	-	-	-	-	1:300	
**Troponin T**	ThermoScientific	MA5-12960	(13-11)	IgG	m	39	47-48		1:50	1:50	1:400	
**Phospho-Wee1 (Ser642)**	Cell Signaling	4910	(D47G5)	IgG	r	95	110		1:25	-		
**Wnt3a**	Cell Signaling	2721	(C64F2)	IgG	r	42	44		1:25	1:25		
**Mitotic Spindle Apparatus**	R&D	test sample		IgG	m	-	-	-	-	-	-	1:50
