# Supplementary material for: Unraveling the Inconsistencies of Cardiac Differentiation Efficiency Induced by the GSK3β Inhibitor CHIR99021 in Human Pluripotent Stem Cells
Source: Stem Cell Reports. 2018 Apr 26;10(6):1851–66. doi: 10.1016/j.stemcr.2018.03.023 (PMC5989659; doi:10.1016/j.stemcr.2018.03.023)
Supplement: Document S2. Article plus Supplemental Information [file mmc2.pdf]

Stem Cell Reports
Article
OPEN ACCESS

Unraveling the Inconsistencies of Cardiac Differentiation Efficiency Induced by the GSK3β Inhibitor CHIR99021 in Human Pluripotent Stem Cells

Filip Laco,[1,*] Tsung Liang Woo,[1] Qixing Zhong,[2] Radoslaw Szmyd,[3] Sherwin Ting,[1] Fahima Jaleel Khan,[1] Christina L.L. Chai,[2] Shaul Reuveny,[1] Allen Chen,[1] and Steve Oh[1,*]
[1]Bioprocessing Technology Institute, 20 Biopolis Way, Centros #06-01, Singapore 138668, Singapore
[2]Department of Pharmacy, Faculty of Science, National University of Singapore, 18 Science Drive 4, Singapore 117543, Singapore
[3]Institute of Molecular and Cell Biology, 61 Biopolis Drive, Proteos #03-01, Singapore 138673, Singapore
*Correspondence: filiplaco@gmail.com (F.L.), steve_oh@bti.a-star.edu.sg (S.O.)
https://doi.org/10.1016/j.stemcr.2018.03.023

SUMMARY

Cardiac differentiation efficiency is hampered by inconsistencies and low reproducibility. We analyzed the differentiation process of multiple human pluripotent stem cell (hPSC) lines in response to dynamic GSK3β inhibition under varying cell culture conditions. hPSCs showed strong differences in cell-cycle profiles with varying culture confluency. hPSCs with a higher percentage of cells in the G1 phase of the cell cycle exhibited cell death and required lower doses of GSK3β inhibitors to induce cardiac differentiation. GSK3β inhibition initiated cell-cycle progression via cyclin D1 and modulated both Wnt signaling and the transcription factor (TCF) levels, resulting in accelerated or delayed mesoderm differentiation. The TCF levels were key regulators during hPSC differentiation with CHIR99021. Our results explain how differences in hPSC lines and culture conditions impact cell death and cardiac differentiation. By analyzing the cell cycle, we were able to select for highly cardiogenic hPSC lines and increase the experimental reproducibility by predicting differentiation outcomes.

INTRODUCTION

Glycogen synthase kinase-3β (GSK3β) has multiple cellular substrates, and they play strategic roles in various essential physiological processes, such as development, the cell cycle, and apoptosis. The main focus of GSK3β in stem cells is associated with its role as a signal transduction factor of the canonical Wnt/β-catenin pathway through the modulation of the GSK3β/β-catenin protein complex via Wnt ligands. GSK3β phosphorylates β-catenin, among other proteins (e.g., cyclin D1), leading to their degradation. The absence of Wnt ligands or the inhibition of GSK3β by growth factors (e.g., fibroblast growth factor 2) and small molecules (e.g., CHIR99021) suppresses substrate phosphorylation by inactivating GSK3β (McCubrey et al., 2014). The canonical Wnt/β-catenin signaling pathway has been suggested to regulate the self-renewal of human pluripotent stem cells (hPSCs) (Sato et al., 2004). Inactivated GSK3β allows the accumulation of β-catenin in the cellular cytosol, which transfers to the nucleus. Nuclear β-catenin forms a complex with transcription factor (TCF) proteins to activate the Wnt pathway gene targets (McCubrey et al., 2014). These Wnt gene targets affect the expression of pluripotency and developmental factors associated with the primitive streak and the germ layers (Hodar et al., 2010). Short-term Wnt induction maintains pluripotency, whereas long-term induction via GSK3β inhibition induces stem cell differentiation to endo- and mesoderm derivatives (Huang et al., 2015) and can further solely regulate the developmental division of the mesoderm into the paraxial and lateral mesoderm, which gives rise to the cardiac lineage (Tan et al., 2013). Efficient cardiac differentiation has been demonstrated with GSK3β inhibition via the small-molecule inhibitor CHIR99021 (CHIR) (Lian et al., 2012). However, the reproducibility of the protocol requires cell line- and cell culture-dependent optimization and can easily lead to heterogeneous differentiation results (Sepac et al., 2012). Moreover, it is not clear how a single transient induction with a GSK3β inhibitor is able to direct highly efficient lineage specification toward cardiomyocytes. Therefore, we studied the effect of CHIR induction in hPSC lines to understand its dynamics and facilitate mesoderm formation resulting in cardiac differentiation.

CHIR is a kinase inhibitor of GSK3α and GSK3β, with off-target effects on kinases within the CDK2-cyclin A2/E cell-cycle complex (An et al., 2014). Moreover, GSKα/β regulates the cell cycle via the mediation of cyclin D1/E (McCubrey et al., 2014) and the chromatin alignment of mitotic cells (Tighe et al., 2007; Yoshino and Ishioka, 2015). GSK inhibitors, such as AR-A014418, CHIR99021, CHIR98014, BIO, and SB-216763, have been reported to induce dose-dependent cell apoptosis in cancer and mouse embryonic stem cells (Naujok et al., 2014; Yoshino and Ishioka, 2015). hPSC differentiation with GSK3β inhibitors often underreports aspects of cell death, which are an essential part of developmental processes and applied bioprocess technologies. Therefore, in this study, we

This is an open access article under the CC BY license (http://creativecommons.org/licenses/by/4.0/).

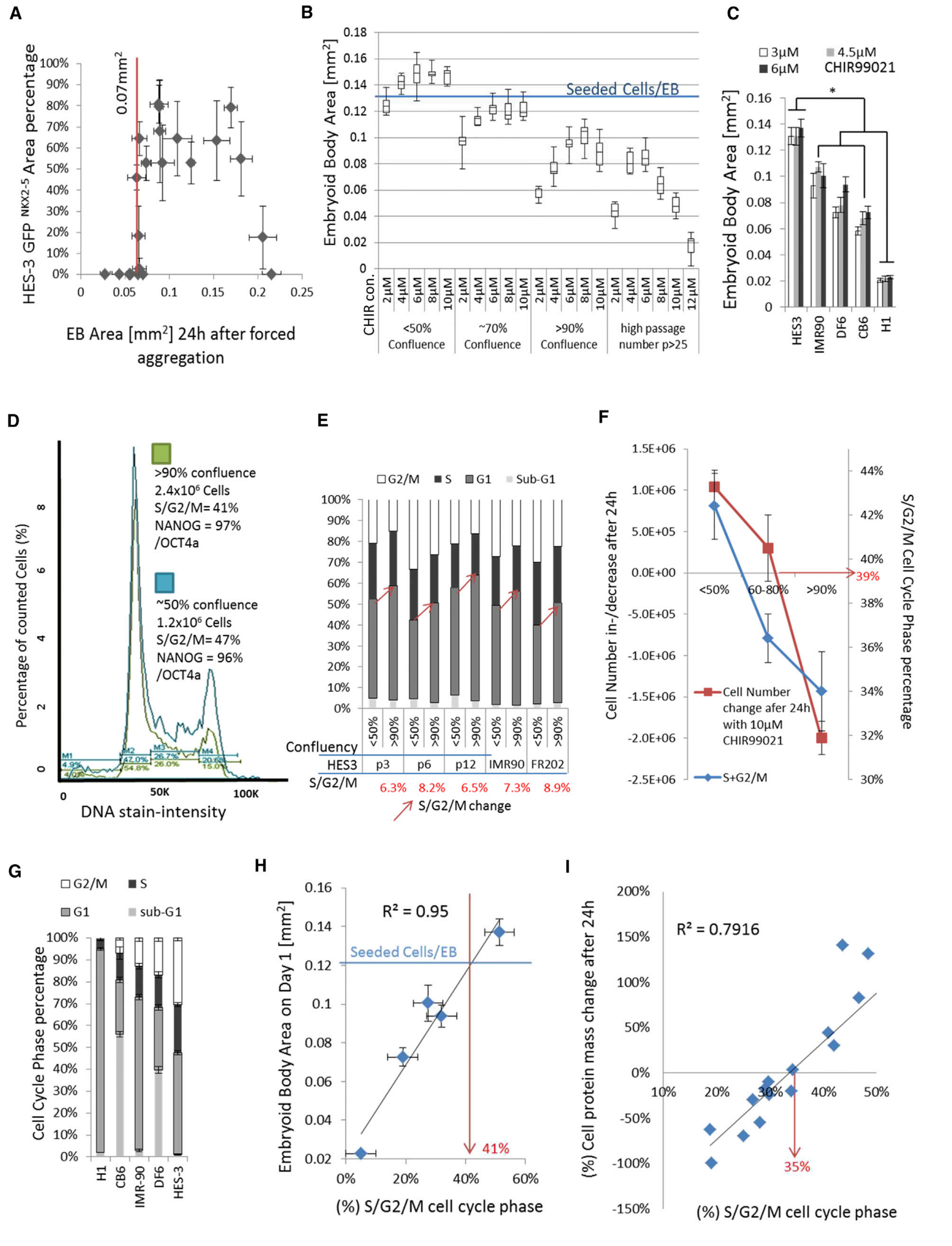

(legend on next page)

examined the effect of CHIR not only on hPSC line differentiation but also on cytotoxicity, cell growth, and the cell cycle.

We demonstrated that CHIR affected the cell cycle and differentiation simultaneously during the initial phase of differentiation. Changes in cell culture (e.g., cell culture density) affect the cell cycle and the dose dependency of CHIR to induce cardiac differentiation. The denser the cell cultures and the lower the S and G2 cell-cycle phases of hPSCs, the stronger was the cytotoxic effect of CHIR induction and the lower were the required doses of this inhibitor to induce cardiac differentiation, which led to decreased cardiac differentiation efficiency. Moreover, CHIR-induced mesoderm and cardiac differentiation by TCF level modulation and cell-cycle cyclin expression. Increased CHIR concentrations accelerated mesoderm development but required well-timed Wnt inhibition via TCF regulation to direct differentiation toward cardiomyocytes. We conclude that the differentiation process with GSK3β inhibition of hPSC lines should account for the initial compound cytotoxicity, the cell density, the cell-cycle state, the dose of CHIR, and the timing of TCF-regulated Wnt inhibition.

RESULTS

GSK Inhibitor CHIR Regulates EB Formation and Cytotoxicity in hPSCs Depending on the Cell Cycle and the Cell Culture Density

In this section, the effect of culture confluency levels, namely, low (<50%), mid (70%), and high (>90%), on embryoid body (EB) formation and monolayer growth in the presence of CHIR was evaluated. We theorize that cell culture induces changes in the cell cycle, changing the cellular response to CHIR. We measured cell death and cell growth in terms of EB size, metabolism, and cell numbers, and related our findings to the cell-cycle phases of hPSCs.

EB differentiation with the optimized EB standard protocol (Supplemental Experimental Procedures) induced a 50%–90% GFP/NKX2-5-positive EB area (Figure 1A). Interestingly, we observed in repeated HES3 differentiation experiments (n = 22, >160 EBs) using initial cell culture conditions, such as 60%–80% cell confluency, <10 cell culture passages, >85% NANOG and OCT4a cell population expression by flow cytometry, and a constant number of seeding cells (15,000 cells/EB), that a wide range of initial EB sizes were formed (0.025–0.225 mm^2 or 2,500–22,500 cells/EB) on day 1. In addition, 22% of the experiments failed to induce GFP expression. Only HES3 EB sizes between 0.07 mm^2 (>7,000 cells/EB) and 0.18 mm^2 (<18,000 cells/EB) were able to express NKX2-5 (Figure 1A). Thus, we analyzed differences in cell culture conditions. The EB size decreased with increased culture density (Figure 1B). The EB size decreased when the CHIR concentration was <6 or >10 μM and in highly confluent cultures that were passaged repeatedly (Figure 1B). Cell loss was also induced in monolayer cultures with a 10 μM CHIR concentration with increasing cell density (Figure S1A). Individual cell lines showed different EB formation sizes after CHIR induction (Figure 1C). In addition, in monolayer cultures, we observed a similar cell line-dependent total protein loss or gain when the cells were cultured with 4–12 μM CHIR (Figure S1B, Table S2). The data indicate a cytotoxic effect of CHIR depending on the initial cell confluency and the cell line. We analyzed the pluripotency, mitotic activity, metabolites, and cell cycle of hPSC cells treated with CHIR to characterize differences between culture conditions and cell lines. Cell density did not affect the pluripotency population percentage of NANOG and OCT4a in HES3 cells (Figure 1D). The cell metabolism (MTT) of high-density cultures was reduced and declined upon differentiation induction (Figure S1C). The consumption of glucose and glutamine and the lactate production per cell were higher in less (<70%) confluent cell cultures, and a more acidified medium (pH 6.88) was

Figure 1. Analysis of the Interaction between Culture Conditions and Cell Cycle in Regard to CHIR Cytotoxicity

(A) Correlation between initial EB area size on day 1 and percentage of the GFP area obtained after 10 days of differentiation (induction for 24 hr with 6 μM CHIR) (n = 4).

(B) EB area size of 1-day-old forced aggregated EBs (12,500 cells/EB) generated from passage 10 (50%, 70%, and 90% culture confluency) and passage 25 (80% culture confluency) cell cultures (n = 16).

(C) EB area size of 5 cell lines after 24 hr CHIR treatment (n = 10, $^*p \leq 0.05$).

(D) Cell cycle, pluripotency, and cell count measurements of HES3 cells cultured at 50% and 90% culture density.

(E) Cell-cycle profiles of IMR90, FR202, and HES3 passages 3, 6, and 12 cultured at 50% and 90% culture densities; average change of S/G2/M cell-cycle profile in hPSCs between 50% and 90% cell culture confluency was 7.4% ± 1.1% (n = 5).

(F) Cell number change after treatment with CHIR and S/G2/M cell-cycle percentage differences of HES3 cells cultured at high (>90%), mid (60%–80%), and low (<50%) culture densities (n = 3).

(G–I) Cell-cycle profiles of 5 hPSC lines (G) and their correlation between the percentage of S/G2/M cell-cycle phase (n = 3) and the measured EB area on day 1 after 24 hr CHIR induction (n = 8) (H). Correlation between percentage of S/G2/M cell-cycle phase of 17 hPSC lines and cell culture passages in monolayer cell culture and their change in cell protein mass (%) after 24 hr CHIR induction (I).

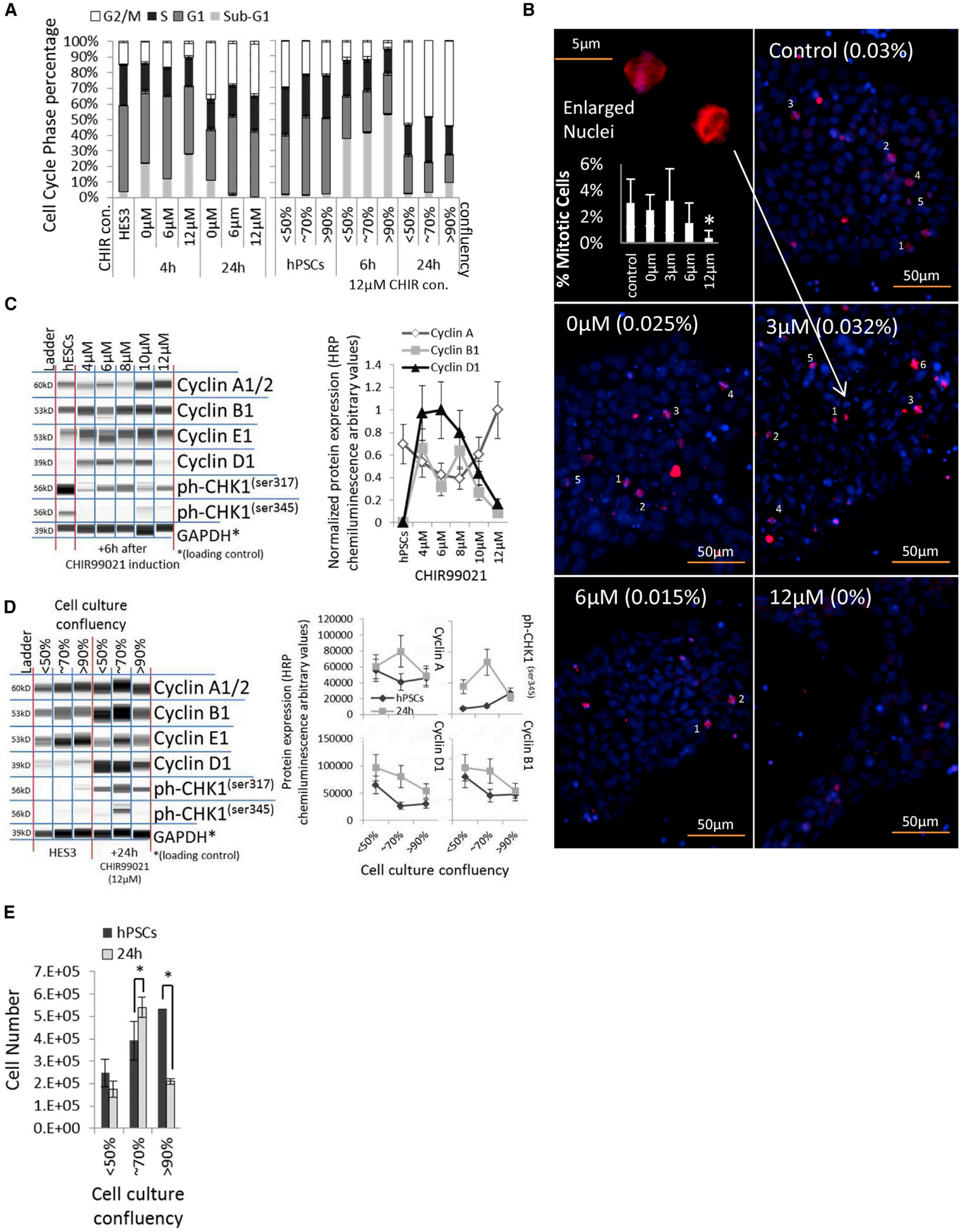

(legend on next page)

observed in highly confluent cultures (>80%) (Figure S1A). Cell-cycle analyses of S/G2/M showed a difference of 7% (±1%) between high (>90%) and low (<50%) cell culture density across passages and cell lines (Figures 1D and 1E). Furthermore, we showed that an increase in cell density led to a decrease in the S/G/2M cell-cycle phases and a gradual loss of cell number after 24 hr with CHIR induction (Figures 1F and S1A). The cell cycle was shown to vary among cell lines, clones, and passage numbers (Figures 1E, 1G, and S1D; Table S2). We correlated the S/G2/M cell-cycle phases of five cell lines with the resulting EB formation area after 24 hr. In addition, we repeated that experiment in monolayer cultures, measuring protein mass changes in correlation with the S/G2/M or G2/M cell-cycle percentage (Figures 1G and 1H; Table S2). The data showed clear correlations between the cell cycle and both EB area and cell protein mass changes.

Our results demonstrate that CHIR was essential for EB formation but also induced cell death and growth in a dose-dependent manner in both EB and monolayer cultures. hPSC lines, clones, and passages showed significant differences in their S/G2/M cell-cycle profiles. Highly confluent cultures showed reduced metabolic activity and had a 7% lower S/G2/M cell-cycle phase when compared with 70%–50% confluent cell cultures. The difference in metabolism translated to cell growth or cell death after CHIR induction, resulting in over 50% cell mass difference between high and low cell culture confluence. CHIR was significantly cytotoxic in culture conditions and cell lines with a low (<35%–41%) S/G2/M cell-cycle phase. Conclusively, a high S/G2/M cell-cycle phase of >42% increased the cell mass by approximately 8% per S/G2/M cell-cycle profile percentage in hPSCs.

CHIR Induces Initial Cell Death and Subsequent Cell Proliferation via Cell-Cycle Progression

In this section, we hypothesize that two separate effects of cell death and cell growth are induced upon CHIR induction in cells of a certain cell-cycle phase. Therefore, the fraction of apoptotic cells in a cell-cycle phase was identified, and the recovery of hPSCs post-CHIR treatment in terms of cell numbers, the cell cycle, and γ-tubulin and cyclin expression were examined.

Cell death of hPSCs occurred within 4–6 hr after differentiation initiation. The percentage of aberrant cells (sub-G1 percentage) increased, whereas the G1 and G2/M cell-cycle percentages decreased significantly in >90% confluent cell cultures and in cell cultures treated with both 0 and 12 μM CHIR (Figure 2A). The higher the CHIR concentration, the fewer proliferating, γ-tubulin-stained cells were quantified (Figure 2B). Moreover, the cell-cycle progression markers cyclin A and B1 were reduced in most cell culture conditions after 12 μM CHIR induction, indicating a loss of proliferating cells in the G2 cycle phase (Figure S2A). However, 24 hr after CHIR induction, the S/G2/M cell-cycle profiles were significantly increased in all conditions (Figure 2A), which indicates the hyperproliferation of the remaining non-apoptotic cell population. Cell-cycle progression is indicated by an increased S/G2/M cell-cycle profile in hPSCs and can be activated by cyclin D1 overexpression (Lim and Kaldis, 2013) as a result of GSK3β inhibition (McCubrey et al., 2014). Cyclin D1 expression levels responded to CHIR in a dose-dependent manner in HES3 EBs (Figure S2B). Induction with 4–8 μM CHIR induced cyclin D1 and B1 expression after 6 hr (Figure 2C). Cyclin D1, A1, and B1 remained expressed after 24 hr in cell culture, with <70% confluency in monolayer and EB cultures (Figures 2D and S2C), resulting in significant cell proliferation in comparison with high-density cell cultures (Figure 2E). We compared cyclin expression in four cell lines and found that cyclin D1 induction was induced in cell lines that showed an increased EB area after 24 hr of induction with CHIR (Figures S2D and S2E). Moreover, these cell lines expressed Troponin T after 14 days (Figure 2E). In addition, we confirmed cyclin D1 expression levels responded to CHIR in a dose-dependent manner in monolayer experiments with three cell lines (Figure S2F). Cyclin D1 was saturated at the peak Troponin T expression of each individual cell line, indicating a correlation between cyclin D1 expression and cardiac development (Figures S2F and S2G).

Interestingly, cell-cycle checkpoints and markers for DNA damage repair, such as CHK2, yH2A.X, p16, p21, GADDA45 (data not shown), and CHK1$^{ser345/317}$ (Figures S2A and 2C), were not expressed or weakly expressed in hPSCs.

Figure 2. Analysis of hPSC Cell Cycle and Cyclins during CHIR Induction
(A) Cell-cycle profile of HES3 cultures after induction with 0–12 μM CHIR, and cell-cycle profile of hPSCs cultured at 50%, 70%, and 90% culture confluency after 12 μM CHIR induction (n = 3).
(B) Nuclei; DAPI (blue) and spindle apparatus of mitotic cells; γ-tubulin (red) staining of monolayer HES3 cultures treated for 8 hr with 0–12 μM CHIR. Numbers in brackets give the percentage of positively stained cells (n = 3 with 10 regions of interest each, *p ≤ 0.05).
(C) Whole-cell protein blot expressions and quantitative analyses of HES3 cells after 4–12 μM CHIR induction.
(D) Whole-cell protein blot expressions and quantitative analyses of HES3 cultures at 50%, 70%, and 90% culture confluency after 12 μM CHIR induction.
(E) Cell number of hPSC cultures at 50%, 70%, and 90% culture confluency after 12 μM CHIR treatment (n = 3, *p ≤ 0.05).

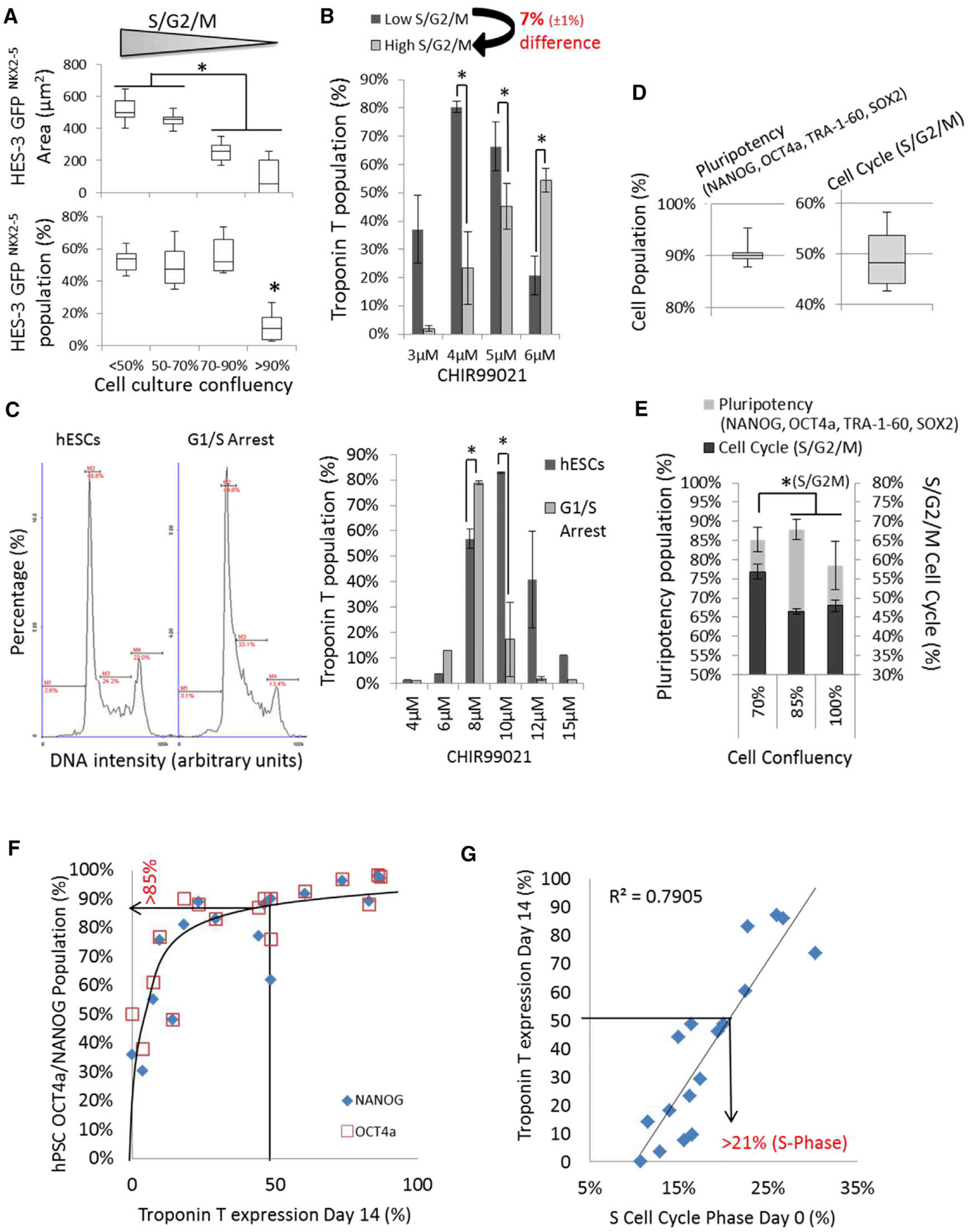

Figure 3. Analysis of hPSC Cell Cycles and Pluripotency in Relationship to Cardiac Differentiation with CHIR

(A and B) Day 11 single-EB GFP/NKX2-5 expression area and percentage of differentiated (induction for 24 hr with 6 μM CHIR) HES3 cells cultured at <50%, 50%–70%, 70%–90%, and >90% culture confluency of low to high S/G2/M cell-cycle profiles (n = 8) (A). Day 11 Troponin T flow cytometry of EBs from differentiated IMR90 cell cultures with a 7% difference in S/G2/M cell-cycle profile (n = 4, $*p \leq 0.05$) (B).

(C) Cell-cycle profiles of HES3 cells and G1/S cell-cycle-arrested cells. Troponin T expression of differentiated HES3 with CHIR on day 14 (n = 3, $*p \leq 0.05$).

(D) Average pluripotency flow cytometer population of pluripotency markers and the percentage of positive population of the S/G2/M cell-cycle profile in hPSCs (n = 12).

(legend continued on next page)

Our results demonstrate that cell death is transient upon cardiac differentiation with CHIR. Proliferative G2 cells diminished with increased cell confluency and CHIR doses. hPSCs did not express markers for DNA damage, indicating a loss of cell-cycle-driven DNA repair mechanisms (Desmarais et al., 2012, 2016). CHIR induced cyclin D1 expression. Hence, cell-cycle progression was induced, increasing cells in the S/G2/M cell-cycle phases and the expression of cyclin A and B1 after 24 hr. Cell growth was significantly increased with a culture confluency of <70%. However, differences in CHIR concentration and cell line dependency were noted.

Cardiac Differentiation Efficiency of hPSCs with CHIR Is Regulated through the Cell Cycle, a Critical Factor for Differentiation Reproducibility and Cell Line Selection

We theorize in this section that the cell-cycle differences of hPSCs in response to CHIR affect cell fate decisions. Therefore, the cell-cycle and pluripotency changes in different culture conditions were examined and correlated with cardiac differentiation expression results.

The NKX2-5-positive EB area decreased with increasing cell culture density (decreasing S/G2/M cell-cycle percentage) (Figure 3A), and the cardiac differentiation efficiency dropped significantly from 50% to 15% GFP/NKX2-5 expression at >90% culture confluency (Figure 3A). Similar results were observed in monolayer cultures with the IMR90 and FR202 cell lines, whereas HES3 cells were not affected at a 12 μM CHIR concentration (Figure S3A). However, a lower CHIR concentration was able to rescue the cardiac efficiency of high versus low (culture density) S/G2/M cell-cycle profile differences of 7% in IMR90 and HES3 EBs (Figures 3B and S3B). Alternatively, a timely induction with a Wnt inhibitor was able to partially rescue cardiac differentiation (Figure S3C). The results were confirmed by monolayer experiments with three additional cell lines (Figure S3D). Moreover, cell-cycle arrest of hPSCs at the G2 and G1/S transition-induced NKX2-5 expression at a lower CHIR concentration than observed under non-arrested culture conditions (Figure 3C). G1/S-arrested cells showed reduced cell growth and G2 arrest that led to cell death (Figure S3E), indicating an apoptotic sensitivity toward proliferating cells, as demonstrated previously. Further, we analyzed the pluripotency expression in hPSC cultures. hPSCs with equally high pluripotency marker expression of 90% (NANOG, OCT4a, TRA-1-60, and SOX2) showed large variations across their S/G2/M cell-cycle profiles (Figure 3D). An increase in cell culture confluency from 70% to 85% first led to a decrease of 8% in the S/G2/M cell-cycle profile, and a further increase of cell culture confluency from 85% to 90% decreased the expression of pluripotency markers (Figure 3E). In three tested hPSC lines cultured at >90% confluency, reduced S/G2/M cell-cycle profiles (three out of three cell lines) and pluripotency (one out of three cell lines) were observed when compared with <70% culture confluence (Figure S3F). This finding indicates that the loss of pluripotency might have contributed to the changes in cardiac differentiation. Hence, we compared NANOG and OCT4a expression with Troponin T expression after differentiation (Figure 3F; Table S2). The data showed a positive asymptotical correlation of NANOG and OCT4a expression with Troponin T expression. Although NANOG and OCT4a cell populations above 85% are likely to induce the cardiac differentiation of >50% of the Troponin T population, these markers were not sufficient to predict the cardiac differentiation efficiency across all cell lines. The cell cycle proved to be more sensitive than NANOG and OCT4a expression for indicating changes in cell culture and the resulting Troponin T expression (Figures 3D and 3E). Hence, we correlated the different cell-cycle phases with Troponin T expression. The S/G2/M and S-phase cell-cycle profiles showed a linear correlation with the cardiac differentiation results (R^2 = 0.6/0.8) across several cell lines (Figure 3G; Table S2). The data show that an S phase cell-cycle percentage of above 21% in multiple hPSC lines is indicative of the induction of >50% Troponin T expression with an optimized CHIR concentration.

In summary, a combined high S/G2/M cell-cycle profile percentage (S phase >21%, G2/M >20%) and a pluripotency of >85% (NANOG and OCT4a) were indicative markers for high-efficiency cell lines, which were likely to achieve a cardiac differentiation of over 50%–90% with a single CHIR induction. Moreover, reductions of the S/G2/M cell cycle by 5%–7% based on the initial cell culture confluency led to a loss of cardiac differentiation efficiency, which was restored by reducing the CHIR concentration by 2 μM in monolayers and 1.5 μM in EB culture experiments. We conclude that a high and stable S/G2/M cell-cycle profile in hPSCs is an important indicator of cell line selection,

(E) Average pluripotency flow cytometer population of pluripotency markers and the percentage of positive population of the S/G2/M cell-cycle profile in hPSCs (n = 3, *$p \leq 0.05$).
(F) Asymptotic correlation of NANOG and OCT4a flow cytometry population (%) of 17 monolayer hPSC cell cultures on day 0 against peak Troponin T cardiomyocyte population on day 14 after 4–12 μM CHIR induction.
(G) Correlation of peak Troponin T cardiomyocyte population on day 14 after 4–12 μM CHIR induction of 17 independent monolayer hPSC cultures against S cell-cycle phase (%) on day 0.

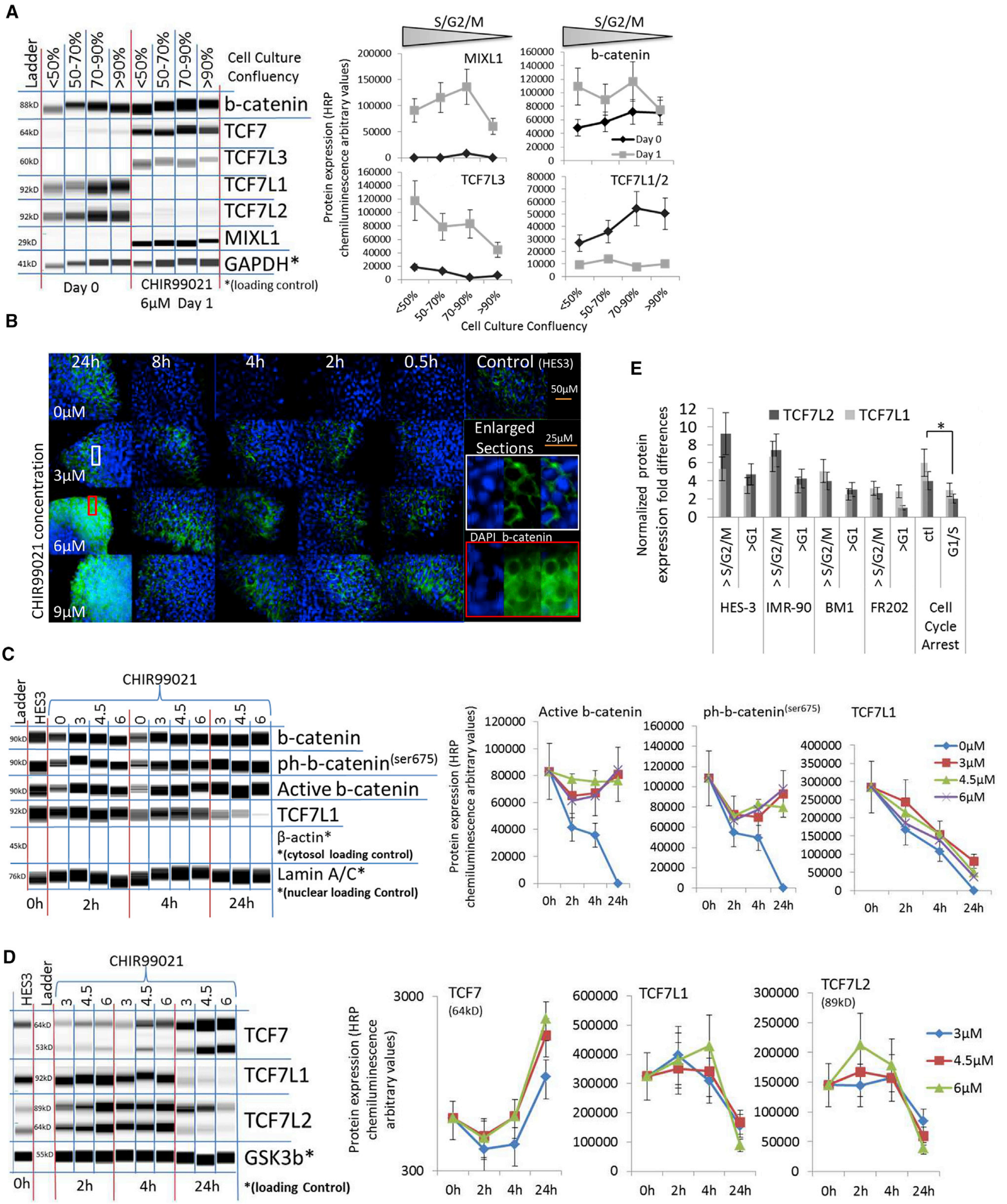

Figure 4. β-Catenin and TCF Modulations after CHIR Induction

(A) Whole-cell protein blot expressions and quantitative analyses of HES3 cells cultured at <50%, 50%–70%, 70%–90%, and >90% cell culture confluency after CHIR induction.

(legend continued on next page)

in addition to pluripotency markers, and an essential factor for cardiac differentiation reproducibility with CHIR.

GSK Inhibition with CHIR Is Controlled by the Wnt Signaling Pathway via TCF Levels

In this section, the protein expression levels of the Wnt signaling pathway were examined after CHIR induction. We measured quantitatively the time- and dose-dependent activation of Wnt protein expression, such as TCFs and β-catenin, in EB and monolayer cultures of different densities and identified the cellular locations of the expressed proteins. We hypothesize that cell culture and cell-cycle differences have an effect on β-catenin/Wnt and TCF regulation upon CHIR99021 induction.

The S and G2 cell-cycle phases are known to induce β-catenin/Wnt pathway signaling via TCF modulations (Ding et al., 2014), which are a potent driver of cardiac differentiation. We measured TCF levels and expression of the primitive streak development marker MIXL1 in EB and monolayer cultures of different confluency and various cell lines with high to low S/G2/M cell-cycle profiles (Figure 4A, S4A, and S4B). TCF7L1/2 was expressed in hPSCs and downregulated in all cell lines and culture conditions after CHIR induction (Figures 4A, S4A, and S4B). Cell lines and culture conditions with a comparatively higher S/G2/M cell-cycle profile showed increased MIXL1 and T-Brachyury and Wnt activator TCF7/TCF7L3 expression when compared with high cell seeding conditions and cell lines with low S/G2/M cell-cycle profiles (Figures 4A, S4A, and S4B). Interestingly, β-catenin was not upregulated or weakly upregulated in dense culture conditions with a lower S/G2/M cell-cycle percentage (Figures 4A, S4A, and S4B). The current hypothesis in Wnt-induced cardiac differentiation focuses on a Wnt/β-catenin-driven mechanism of nuclear β-catenin transfer and accumulation (McCubrey et al., 2014; Ye et al., 2012). Active β-catenin was located in the cellular membrane and did not increase during the first 4–8 hr of CHIR treatment (Figures 4B and S4C). β-Catenin accumulated in the cytosol after 24 hr when 6–9 μM CHIR was applied (Figure 4B). Interestingly, the nuclear protein expression levels of the β-catenins showed no significant changes with 3–6 μM CHIR treatment in EBs (Figures 4B and 4C). However, a CHIR concentration of >6 μM induced β-catenin and transcriptional β-catenin expression in monolayer cultures (Figures S4A and S4B). Nuclear β-catenin translocation was observed with the FR202 cell line at 15 μM CHIR (Figure S4D). Nuclear protein expression of β-catenin was limited in IMR90 cells but increased significantly in the FR202 cell line (Figures S4E and S4F). Moreover, we observed that cell-cycle profile differences of 5%–6% inhibited β-catenin expression in IMR90 cells and limited β-catenin expression in the FR202 cell line; nevertheless, TCF and primitive streak markers were expressed (Figures S4A and S4B). Moreover, we observed a significant decline of nuclear and cytosolic TCF7L1 levels 4 or 6 hr after CHIR induction in EB and monolayer cultures with all tested CHIR concentrations and the successive induction of primitive streaks and the TCF7 and TCF7L3 markers 24 hr later (Figures 4A, 4C, S4A, S4B, S4E, and S4F).

The transcription of TCF7 and TCF7L3 is induced by β-catenin nuclear translocation, whereas TCF7L1/2 is a negative regulator of the nuclear Wnt signaling pathway (Yi et al., 2011). Thus, we measured TCF7, TCF7L1, and TCF7L2 protein expression levels during CHIR induction. The expression of TCF7L1 and TCF7L2 decreased, while TCF7 increased in a dose-dependent manner in EB and monolayer cultures with the HES3, IMR90, and FR202 cell lines (Figures 4C, 4D, S4E, and S4F). Moreover, we measured the TCF7L1/2 protein expression of >90% confluent (>G1 cell-cycle phase) hPSCs and 70% confluent hPSCs with a relatively increased S/G2/M phase (Figure 4E). TCF7L1/2 expression was decreased in dense cell culture conditions (>G1 cell-cycle phase) in most cell lines. Dense culture conditions required lower (1.5–2 μM) CHIR concentrations to induce cardiac differentiation efficiently, as shown previously (Figures 3B and S3D). The data indicate that cell culture conditions affected the cell cycle of hPSCs, which modulated the levels of Wnt, inhibiting TCF7L1/2 proteins; thus, the CHIR concentration for cardiac differentiation had to be adjusted. Furthermore, we confirmed that G1/S cell-cycle arrest reduced TCF7L1/2 expression in IMR90 and FR202 cells and increased transcriptional β-catenin expression (Figure 4E and S4G). Moreover, cyclin A and cell-cycle checkpoints were expressed after cell-cycle arrest. The data showed that TCF7L1/2 was modulated by cell-cycle manipulations, but additional factors, such as β-catenins, cyclins, and cell death, are part of the complex

(B) Fluorescence microscopy of immuno-cytochemistry-stained HES3 cells against active β-catenin (green) and DAPI (blue) after CHIR induction.

(C) Nuclear fraction protein blot expressions and quantitative analyses of HES3 EBs after CHIR induction. Note sample CHIR 0 μM degraded after 24 hr.

(D) Whole-cell protein blot expressions and quantitative analyses of HES3 EBs after CHIR induction.

(E) Normalized TCF7L1/2 protein expression folds of hPSCs cultured at 90% cell confluency with increased G1 cell-cycle phase compared with 70% cell confluency, with an increased S/G2/M and average TCF7L1/2 protein expressions of hPSCs after G1/S cell-cycle arrest (n = 3, $^*p \leq 0.05$).

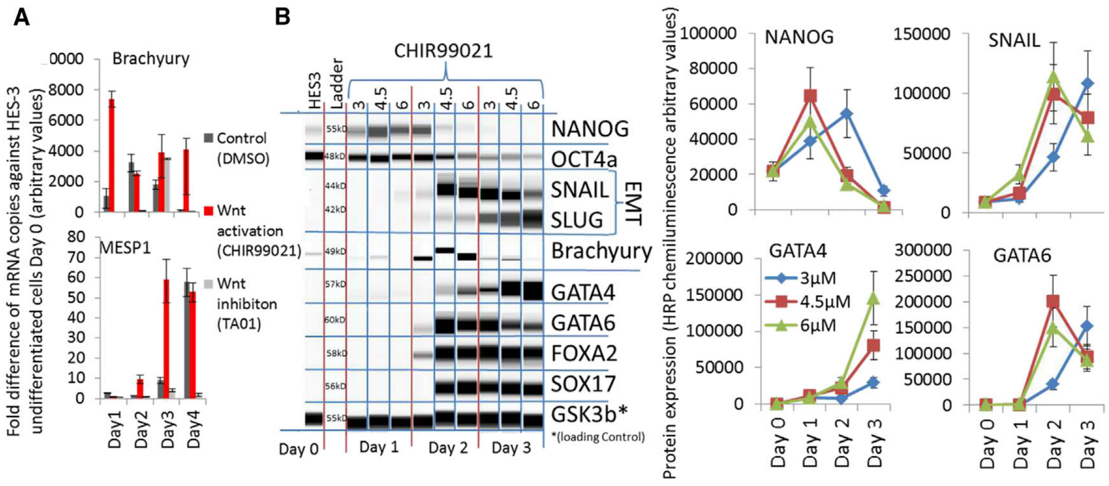

Figure 5. Acceleration of Mesodermal Development after CHIR Induction

(A) qPCR analyses of fold increase/decrease over HES3 control after treatment of HES3 EBs with 3 μM CHIR, DMSO control, and 5 μM Wnt inhibitor TA-01 for 24 hr (n = 3).

(B) Whole-cell protein blot expressions and quantitative analyses of HES3 cells and differentiating EBs after CHIR induction.

mechanism of Wnt regulation during CHIR-induced differentiation.

In summary, modulations of β-catenin levels after CHIR induction were cell line- and cell culture dependent. β-Catenin translocated from the membrane to the cytosol and increased β-catenin levels in hPSCs when cultured at 50%–70% culture confluency with over >6 μM CHIR. Interestingly, hPSCs with a culture confluency of 90% and a reduced S/G2/M cell-cycle profile showed a small increase in β-catenin levels. Nevertheless, all tested concentrations and culture conditions induced primitive streaks, the early mesoderm and TCF7 and TCF7L3 marker expression. More importantly, the Wnt regulators TCF7L1 and TCF7L2 decreased in the nuclear fraction prior to TCF7 and TCF7L3 expression. Overall, TCF levels responded within 4–24 hr to CHIR induction in a manner dependent on dose and culture conditions, indicating that TCFs play a key role as a Wnt regulatory mechanism.

GSK Inhibition with CHIR Enhances Mesoderm Differentiation Independent of Cell Culture and Cell-Cycle Manipulation

In this section, primitive streak and early mesoderm development was examined in terms of transcription factor and protein expression. We measured the development of EB and monolayer cultures after CHIR induction at various doses and with cell-cycle arrest and compared the results with the cardiac differentiation efficiency. We hypothesize that both the CHIR doses and the cell cycle affect mesoderm development and therefore cardiac differentiation.

The expression of T-Brachyury was induced on day 1 after CHIR treatment, followed by MESP1 expression on days 2–4 in hPSC EBs (Figure 5A). These mesodermal inductions were also observed in the non-treated (DMSO) and Wnt-inhibited EBs (TA01) cultures, with a delay of 24–48 hr (Figure 5A). Further, we measured the dose dependency of CHIR in endo-mesoderm development. CHIR concentrations (4.5–6 μM) induced endothelial-mesenchymal transition (Slug, Snail), endo-mesoderm development (GATA4, -6, FOXA2, and SOX17) and pluripotency decline (NANOG and OCT4a) on day 2, whereas a lower CHIR concentration of 3 μM resulted in a 24-hr delay in the protein expression and persistent expression of pluripotent markers (Figure 5B). Similar results were obtained using a microcarrier-based bioprocess culture system with the IMR90 cell line (Figure S5A). CHIR dose-dependent primitive streak induction was also confirmed with four additional lines (X.13, IMR90, 4Skin, and Donor 5) in monolayer experiments (Figure S5B). The induction of T-Brachyury and MIXL1 was strongly cell line dependent (Figure S5B). Interestingly, cell confluency differences with a 5%–6% S/G2/M cell-cycle profile difference did not affect T-Brachyury expression, as shown previously (Figures S4A and S4B). Therefore, we tested the effect of cell-cycle arrest on mesoderm development. The flow cytometry profiles of T-Brachyury and PDGFRa were largely unaffected by G1/S phase-arrested hPSC cultures, but Troponin T expression showed strong differences between the tested conditions (Figure S5C). The Troponin T expression did not correlate with T-Brachyury, MIXL1, or PDGFRa expression (Figures S5B and S5C).

In summary, CHIR-induced primitive streak and mesoderm development (MIXL1, MESP1, T-Brachyury, GATA4, and PDGFRa) in hPSCs maintained in EBs, monolayers,

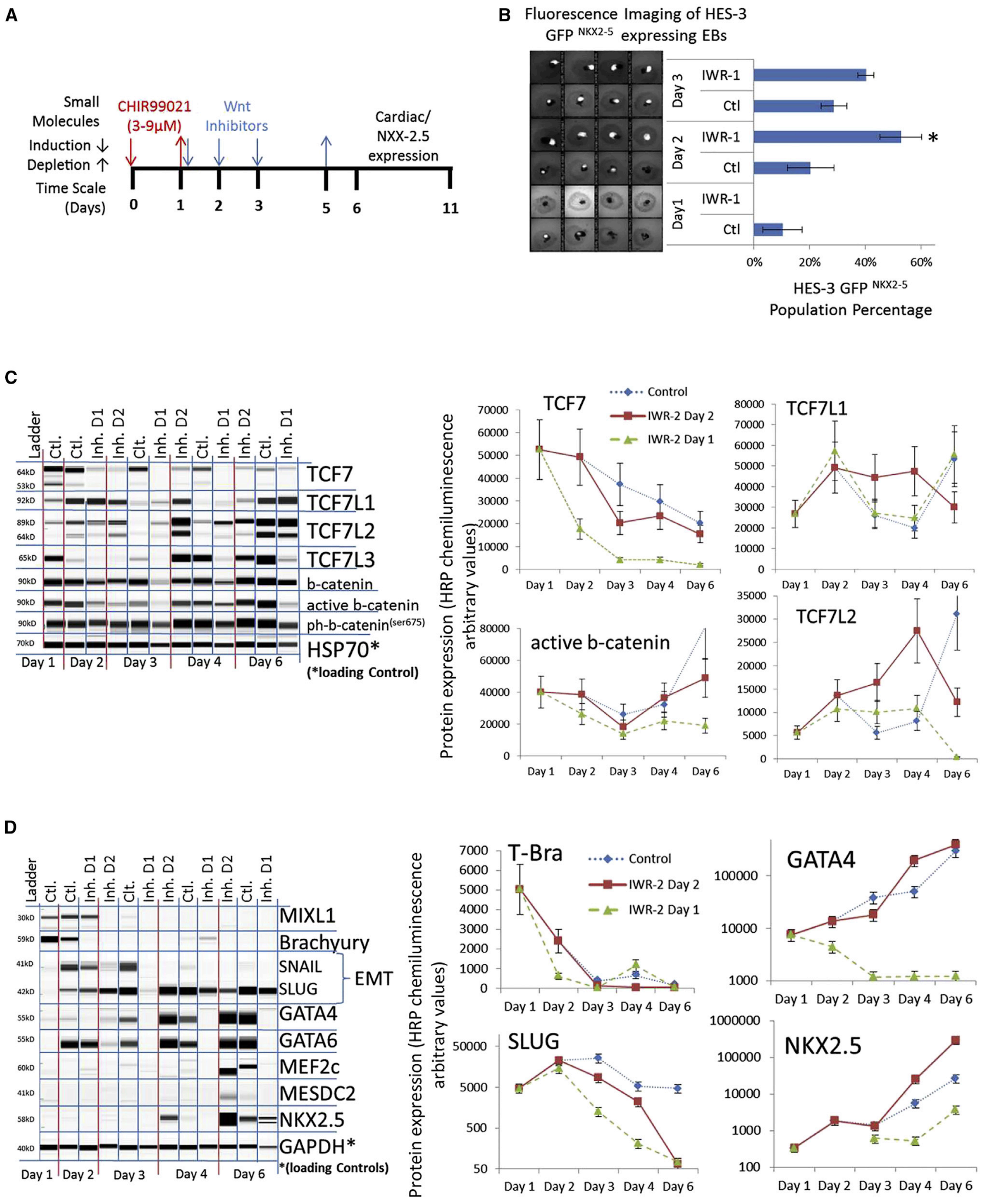

(legend on next page)

and microcarrier-based aggregate cultures. The onset and progression of mesoderm development was CHIR concentration and cell line dependent. Mesoderm development was less affected by hPSC culture confluency/density and cell-cycle changes, but cardiac differentiation was strongly affected by confluency/density and cell-cycle changes.

TCF7L1 and TCF7L2 Expression Is Prolonged by Small-Molecule Wnt Inhibitors to Induce the Expression of Mesoderm Markers and the Cardiac Progenitor NKX2-5

In this section, the depletion of CHIR and the time and dose effects of Wnt inhibitors post-CHIR induction were examined. Wnt pathway proteins, TCF expression, and mesoderm and early cardiac development markers were measured. Reversing the effect of CHIR is thought to limit Wnt signaling and induce differentiation.

Cardiac differentiation can be enhanced by external Wnt inhibition (IWR-1) when applied 1–3 days after CHIR induction in EBs (Figures 6A and 6B; Table S1) and monolayer cultures (Table S2). We measured a strong increase of the mRNA expression levels of the Wnt-negative regulator DKK-1 after CHIR induction (Figure S6A), but protein expression levels of DKK-1 and ph-GSK3$\beta^{(ser9)}$ were low (Figure S6B). Therefore, we analyzed the protein expression levels of TCFs in three EB cultures with IWR-1 (2.5 μM) induction on day 1 (0% GFP/NKX2-5) or day 2 (50% GFP/NKX2-5) or without IWR-1 induction (25% GFP/NKX2-5) as the control condition (Figure 6B). The TCF7L1 and TCF7L2 levels increased after CHIR withdrawal on day 2 but returned to their previous levels on day 3 (Figure 6C). IWR-1 prolonged TCF7L1 and TCF7L2 expression when applied on day 2 (Figure 6C). When applied on day 1, β-catenin, TCF7, primitive strike, mesoderm, and EMT markers were downregulated early by IWR-1, indicating that TCF7 and β-catenin levels might have affected the expression of these direct Wnt gene targets, which disturbed further mesoderm marker expression (Figure 6D). IWR-1 induction on day 2 reduced GATA-4, GATA-6, and EMT on day 3 but induced significantly higher expression of GATA-4 on day 4 and an early onset of the cardiac progenitor marker NKX2-5 (Figure 6D). Other muscle mesoderm markers (MEF2c and MESDC2) were expressed on day 6 (Figure 6D).

In summary, this experiment shows that CHIR depletion leads to a transient increase of Wnt, inhibiting TCF7L1 and TCF7L2 expression levels. Moreover, IWR-1 prolonged the expression of TCF7L1 and TCF7L2, which supported cellular TCF regulation and increased specialization toward the mesoderm and early cardiac lineages.

DISCUSSION

Cytotoxicity of the GSK Inhibitor CHIR in hPSC Cultures Alters Cardiac Differentiation

The importance of small-molecule GSK3β inhibitors is evident in hPSC differentiation and may become an industrial standard for the development of cell-based therapy. However, GSK3β inhibition with CHIR in hPSC lines ($n > 12$) is cytotoxic to cell cultures, especially to those with a low S/G2/M cell-cycle phase. hPSCs are characterized by a high G2 phase (Kapinas et al., 2013). An increase of the G1 phase and a loss of the G2/M cell-cycle phase is due to colony culture conditions and ECM dependency, which restrict cell expansion at the center of the colonies (Jacobs et al., 2016). An accumulation of high sub-G1 and G1 cell-cycle phases in hPSCs is likely due to medium acidification of densely cultured cells (Jacobs et al., 2016), which is known to affect cell growth, metabolism (Chen et al., 2010), and pluripotency (Gupta et al., 2017), and to induce genotoxic stress in hPSCs (Bárta et al., 2010). Cell death occurred upon CHIR induction, especially with high-cell-density cultures. CHIR-induced cyclin D1 expression in hPSCs. Cyclin D1 is known to activate essential genes for S phase entry and DNA synthesis (Lim and Kaldis, 2013), and overexpression could lead to rapid cell growth and replication stress under conditions of restricted mitogenic signaling, bypassing key cell-cycle checkpoints (Qie and Diehl, 2016). Therefore, it can be assumed that CHIR99021-induced expression of cyclin D1 forced the hPSCs into cell-cycle progression, leading to a high rate of premitotic apoptosis due to an accumulation of genetic instability after exposure to genotoxic stress in acidified medium from high-density cell cultures. Although hPSCs have a robust DNA damage repair mechanism prior to G2 entry (Vitale et al., 2017), they have been shown to fail single-strand DNA damage repair due to replication stress and

Figure 6. TCF7L1/2 Expression Regulates Wnt Inhibition and Early Cardiac Differentiation

(A and B) Induction timing for CHIR and Wnt inhibitors during the differentiation process (A). Differentiated HES3 EBs on day 11 expressing GF/NKX2-5 area in white and GFP/NKX2-5 population percentage (B). HES3 EBs were cultured with 6 μM CHIR for 24 hr and with/without 2.5 μM IWR-1 induction on day 1–3 ($n = 4$, $^*p \leq 0.05$).

(C) Whole-cell protein blot expressions and quantitative analyses of HES3 EBs after 6 μM CHIR induction (ctl.) and 2.5 μM IWR-1 inhibition at day 1 (inh. D1) and day 2 (inh. D2).

(D) Protein blot expressions and quantitative analyses of whole-cell HES3 EBs after 6 μM CHIR induction (ctl.) and 2.5 μM IWR-1 inhibition at day 1 (inh. D1) and day 2 (inh. D2).

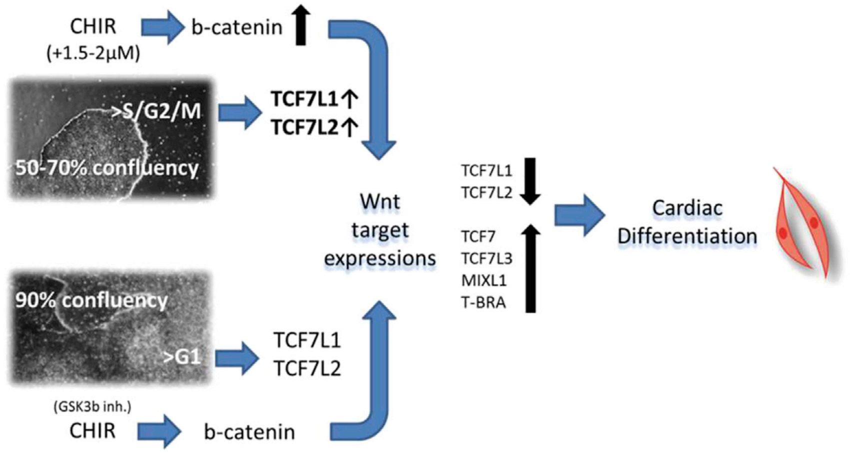

Figure 7. CHIR Modulation in Varying Cell Culture Densities

A lower cell culture confluency (50%–70%) of hPSCs showed an increased S/G2M cell-cycle phase which elevated the expression levels of Wnt inhibitors TCF7L1/2 when compared with higher cell culture densities (90%). CHIR induction required an additional 1.5–2 μM to induce higher β-catenin levels via GSK3b inhibition in order to suppress TCF7L1/2, and activate sufficient levels of the Wnt gene target expression (TCF7, TCF7L3, MIXL1, and T-BRA). A successive time-sensitive Wnt modulation lead to a comparable cardiac differentiation in both cell culture formats.

to induce apoptosis during the intra-S cell-cycle phase (Desmarais et al., 2012, 2016) or lose pluripotency (Vitale et al., 2017). Moreover, the S cell-cycle phase correlated with cardiac differentiation efficiency. In this regard, culture conditions (e.g., cell density, cell passaging, and media) define an initial genomic hPSC state that translates to the differential response to CHIR (Gonzalez et al., 2016). Therefore, it can be assumed that these intrinsic and extrinsic factors induce replicative stress, leading to genomic instabilities. Upon CHIR induction, an immediate cytotoxic effect is present due to cell-cycle progression. Moreover, cardiogenic efficiency with CHIR depends strongly on the genomic stability of hPSCs and could be an indicator of cardiac differentiation efficiency and other CHIR-induced differentiation methods.

TCFs as Wnt Regulators in Mesoderm and Cardiac Differentiation

Wnt induction is directly involved in primitive streak patterning and mesoderm differentiation (e.g., MIXL1, T-Brachyury, ISL1, and BMP4) (Hodar et al., 2010). The accumulation of nuclear β-catenin is thought to induce Wnt gene transcription. Induction with >7 μM CHIR has been shown to induce cytosolic and nuclear β-catenin accumulation (Kempf et al., 2016; Mendjan et al., 2014). However, we show that β-catenin levels are not significantly changed with <6 μM CHIR in EB differentiation protocols. Interestingly, primitive streak and mesoderm marker expression did not depend on the cytosolic or nuclear enrichment of β-catenin. Moreover, TCF levels responded to CHIR in a concentration-dependent manner via GSK3β inhibition. TCFs consist of four paralogs in humans. TCF7L3 expression promotes WNT target activation, whereas TCF7L1 expression represses WNT targets. The expression of TCF7 and TCF7L2 can be associated with both actions depending on the cellular system (Cadigan, 2012). TCF7L1 and TCF7L2 were highly expressed in hPSCs and decreased upon CHIR stimulation, whereas TCF7 and TCF7L3 were weakly expressed but increased strongly after GSK3β inhibition with CHIR. This finding indicates that TCF7L1 and TCF7L2 act as Wnt repressors and that TCF7 and TCF7L3 are Wnt activators in hPSCs. A potential mechanism was observed with a mouse embryonic model in which TCF7L1 was inactivated by β-catenin with CHIR and WNT3a to induce gene transcription (Shy et al., 2013). Interestingly, our data indicate that TCF regulation is induced by the inhibition of GSK3β via CHIR prior to β-catenin nuclear induction. However, it remains unclear how Wnt-regulated transcription targets of primitive streak and mesoderm development are induced. A possible explanation could be that β-catenin was metabolized prior to detection by interacting with TCF7L1/2, as described by Shy et al. (2013) and Hikasa and Sokol (2011). This possibility would explain why cell lines with low TCF7L1/2 expression, such as FR202, exhibit β-catenin translocation, whereas HES3 and IMR90 lacked evidence. Further, Wnt transcription could be induced by pre-existing nuclear expression levels of β-catenin upon depletion of the nuclear Wnt transcription inhibitors TCF7L1 and TCF7L2. This process would lead to Wnt gene target transcription, including TCF7 and TCF7L3 expression. A delayed nuclear induction of β-catenin in combination with the TCF7 and TCF7L3 proteins would amplify Wnt gene transcription and induce a chain of developmental signals toward cardiac differentiation. Although we demonstrate the importance of TCFs here, the role of β-catenin remains paramount, as nuclear levels of β-catenin are essential for the modulation of Wnt transcription and cardiac differentiation.

Dynamic Mechanism of Mesoderm-Cardiac Differentiation

The CHIR time course and dose can delay or accelerate mesoderm development and lead to a variety of lineages.

CHIR activated Wnt by decreasing TCF7L1 and TCF7L2 and inducing TCF7 and TCF7L3. A low CHIR concentration has been shown to lead to endoderm development, which can induce hepatocyte differentiation (Siller et al., 2015). Prolonged or 2- to 3-fold higher CHIR concentrations induced high expression of primitive streak and mesoderm markers, followed by endothelial-mesenchymal transition and cardiovascular progenitor development. A critical step was CHIR withdrawal, which leads to Wnt inhibition and was marked by a transient TCF7L1 and TCF7L2 induction. Wnt inhibition was shown to be essential for suppressing the cardiac deregulator MSX1 (Rao et al., 2015), which induced the cardiovascular markers GATA4 and NKX-2.5. Wnt inhibition induced by CHIR depletion and TCF7L1 and TCF7L2 upregulation was insufficient to induce cardiomyocytes when the initial CHIR doses were too high or temporally overextended. Wnt inhibitors, such as IWP-2, IWR-1, and TA01/02, have been found to support cardiac differentiation during this particular time window (Laco et al., 2014). The transient expression of the Wnt repressors TCF7L1 and TCF7L2 was prolonged for the duration of IWR-1 induction, leading to controlled cardiac differentiation. However, the initial variations in culture conditions (cell confluency and cell cycle) limited the reproducibility. The S and G2 cell-cycle phases are known to induce β-catenin/Wnt pathway signaling via TCF modulations (Ding et al., 2014). A mid to low cell culture confluency (50%–70%) resulted in increased S/G2/M percentages and TCF7L1/2 expression and required lower CHIR doses to induce cardiac differentiation (Figure 7). CHIR induces β-catenin, which interacts with and depletes TCF7L1/2 (Shy et al., 2013) to induce timely Wnt and mesoderm induction, leading to cardiac differentiation (Figure 7). Variations in TCF7L1/2 levels and/or the cell cycle will therefore lead to alternative differentiation results when CHIR concentrations are kept constant. Moreover, pluripotency marker expression levels were often unaffected by cell-cycle changes. Thus, cell-cycle validation is crucial for stem cell quality and differentiation efficiency.

In conclusion, GSK inhibition via CHIR manipulates the cell cycle and TCF/Wnt regulation simultaneously. Our findings not only demonstrate how GSK inhibition interconnects the cell-cycle and cell-fate decisions to direct mesoderm and cardiomyocyte development in hPSCs, but also help to elucidate how the proliferation and cell death of hPSCs are induced during differentiation. Moreover, we elucidate the importance of the cell cycle in CHIR-driven differentiation methods. Thus, we propose that the cell-cycle profile should be part of the regular quality control criteria for hPSCs in order to improve experimental consistency and cell line selection for high differentiation efficiency to cardiac and other mesoderm-derived cell types.

EXPERIMENTAL PROCEDURES

General Mesoderm-Cardiac Differentiation Protocol

The protocol was adapted from Lian et al. (2012). In brief, when hPSCs maintained on Geltrex-coated plates achieved the desired confluence, the cells were treated with the GSK3β inhibitor CHIR (Selleckchem) in differentiation medium for 24 hr. The differentiation medium was either phenol red-free bSFS medium for single EB and Aggrewell differentiation or RPMI/B27-insulin (Life Technologies) for monolayer and microcarrier differentiation. The differentiation medium was changed to remove/reduce CHIR concentrations during the first 3 days. An optional treatment with Wnt inhibitors, such as IWR-1 (Tocris), IWP-2 (Stemgent), or TA01 (Tocris), followed on day 2/3. Wnt inhibitors were removed during the medium change on day 5/6. The cells were maintained in differentiation medium with daily medium changes. The cells were incubated at 37°C in a humidified atmosphere with 5% CO_2. All small molecules were solubilized in DMSO (Sigma). The optimization of the protocol can be found in Table S1 and the Supplemental Experimental Procedures.

SUPPLEMENTAL INFORMATION

Supplemental Information includes Supplemental Experimental Procedures, seven figures, and three tables and can be found with this article online at https://doi.org/10.1016/j.stemcr.2018.03.023.

AUTHOR CONTRIBUTIONS

F.L. designed the experiments. F.L., T.L.W., F.J.K., and Q.Z. performed the experiments. F.L. and T.L.W. performed the computational analysis of the data. F.L. interpreted the experiments and data; additionally, R.S. interpreted the cell-cycle-related experiments and data. F.J.K., S.T., and A.C. provided experimental data related to cardiac bioprocessing and technology translation. F.L. wrote the paper with input from S.O., A.C., C.L.L.C., and S.R.

ACKNOWLEDGMENTS

We thank Stemcell Technologies for providing us with the Aggrewell differentiation plates. We thank Dr. David Elliot for the HES3 NKX2-5 reporter cell line, Dr. Jonathan Loh for the Donor 5 and X.13 iPS cell lines, and Dr. Alan Lam for the FR201 and FR202 iPS cell lines. We thank Dr. Mei Chih Liao, Dr. Gloryn Chia, and Dr. Jaichandran Sivalingam for their valuable scientific insights. This work was supported by A*Star (Agency of Science, Technology and Research) and received funding from the 2015 Biomedical Research Council Young Investigator Grant (project ID: 1515651022), Singapore.

Received: March 7, 2017
Revised: March 28, 2018
Accepted: March 28, 2018
Published: April 26, 2018

REFERENCES

An, W.F., Germain, A.R., Bishop, J.A., Nag, P.P., Metkar, S., Ketterman, J., Walk, M., Weiwer, M., Liu, X., Patnaik, D., et al. (2014).

Discovery of potent and highly selective inhibitors of GSK3b. In Probe Reports from the NIH Molecular Libraries Program (National Center for Biotechnology Information (US)).

Bárta, T., Vinarský, V., Holubcová, Z., Doležalová, D., Verner, J., Pospíšilová, Š., Dvořák, P., and Hampl, A. (2010). Human embryonic stem cells are capable of executing G1/S checkpoint activation. Stem Cells *28*, 1143–1152.

Cadigan, K.M. (2012). TCFs and Wnt/β-catenin signaling. more than one way to throw the switch. Curr. Top. Dev. Biol. *98*, 1–34.

Chen, X., Chen, A., Woo, T.L., Choo, A.B., Reuveny, S., and Oh, S.K. (2010). Investigations into the metabolism of 2D colony and suspended microcarrier cultures of human embryonic stem cells in serum free media. Stem Cells Dev. *19*, 1781–1792.

Desmarais, J.A., Hoffmann, M.J., Bingham, G., Gagou, M.E., Meuth, M., and Andrews, P.W. (2012). Human embryonic stem cells fail to activate CHK1 and commit to apoptosis in response to DNA replication stress. Stem Cell *30*, 1385–1393.

Desmarais, J.A., Unger, C., Damjanov, I., Meuth, M., and Andrews, P. (2016). Apoptosis and failure of checkpoint kinase 1 activation in human induced pluripotent stem cells under replication stress. Stem Cell Res. Ther. *7*, 17.

Ding, Y., Su, S., Tang, W., Zhang, X., Chen, S., Zhu, G., Liang, J., Wei, W., Guo, Y., Liu, L., et al. (2014). Enrichment of the β-catenin-TCF complex at the S and G2 phases ensures cell survival and cell cycle progression. J. Cell Sci. *127*, 4833–4845.

Gonzalez, J.M., Morgani, S.M., Bone, R.A., Bonderup, K., Abelchian, S., Brakebusch, C., and Brickman, J.M. (2016). Embryonic stem cell culture conditions support distinct states associated with different developmental stages and potency. Stem Cell Reports *7*, 177–191.

Gupta, P., igan, K., Jadhav, S., Bellare, J., and Verma, P. (2017). Effect of lactate and pH on mouse pluripotent stem cells: importance of media analysis. Biochem. Eng. J. *118*, 25–33.

Hikasa, H., and Sokol, S.Y. (2011). Phosphorylation of TCF proteins by homeodomain-interacting protein kinase 2. J. Biol. Chem. *286*, 12093–12100.

Hodar, C., Assar, R., Colombres, M., Aravena, A., Pavez, L., Gonzalez, M., Martinez, S., Inestrosa, N.C., and Maass, A. (2010). Genome-wide identification of new Wnt/beta-catenin target genes in the human genome using CART method. BMC Genomics *11*, 348.

Huang, T.-S., Li, L., Moalim-Nour, L., Jia, D., Bai, J., Zemin, Y., Bennett, S.A.L., Fugeys, D., and Wang, L. (2015). A regulatory network involving b-Catenin, E-Cadherin, PI3K/Akt, and slug balances self-renewal and differentiation of human pluripotent stem cells in response to Wnt signaling. Stem Cells *33*, 1419–1433.

Jacobs, K., Zambelli, F., Mertzanidou, A., Smolders, I., Geens, M., Nguyen, H.T., Barbé, L., Sermon, K., and Spits, C. (2016). Higher-density culture in human embryonic stem cells results in DNA damage and genome instability. Stem Cell Reports *6*, 330–341.

Kapinas, K., Grandy, R., Ghule, P., Medina, R., Becker, K., Pardee, A., Zaidi, S.K., Lian, J., Stein, J., van Wijnen, A., and Stein, G. (2013). The abbreviated pluripotent cell cycle. J. Cell. Physiol. *228*, 9–20.

Kempf, H., Olmer, R., Haase, A., Franke, A., Bolesani, E., Schwanke, K., Robles-Diaz, D., Coffee, M., Göhring, G., Dräger, G., et al. (2016). Bulk cell density and Wnt/TGFbeta signalling regulate mesendodermal patterning of human pluripotent stem cells. Nat. Commun. *7*, 13602.

Laco, F., Low, J.-L., Seow, J., Woo, T.L., Zhong, Q., Seayad, J., Liu, Z., Wei, H., Reuveny, S., Elliott, D.A., et al. (2014). Cardiomyocyte differentiation of pluripotent stem cells with SB203580 analogues correlates with Wnt pathway CK1 inhibition independent of p38 MAPK signaling. J. Mol. Cell. Cardiol. *80*, 56–70.

Lian, X., Hsiao, C., Wilson, G., Zhu, K., Hazeltine, L.B., Azarin, S.M., Raval, K.K., Zhang, J., Kamp, T.J., and Palecek, S.P. (2012). Robust cardiomyocyte differentiation from human pluripotent stem cells via temporal modulation of canonical Wnt signaling. Proc. Natl. Acad. Sci. USA *109*, E1848–E1857.

Lim, S., and Kaldis, P. (2013). Cdks, cyclins and CKIs: roles beyond cell cycle regulation. Development *140*, 3079–3093.

McCubrey, J.A., Steelman, L.S., Bertrand, F.E., Davis, N.M., Sokolosky, M., Abrams, S.L., Montalto, G., D'Assoro, A.B., Libra, M., Nicoletti, F., et al. (2014). GSK-3 as potential target for therapeutic intervention in cancer. Oncotarget *5*, 2881–2911.

Mendjan, S., Mascetti, V.L., Ortmann, D., Ortiz, M., Karjosukarso, D.W., Ng, Y., Moreau, T., and Pedersen, R.A. (2014). NANOG and CDX2 pattern distinct subtypes of human mesoderm during exit from pluripotency. Cell Stem Cell *15*, 310–325.

Naujok, O., Lentes, J., Diekmann, U., Davenport, C., and Lenzen, S. (2014). Cytotoxicity and activation of the Wnt/beta-catenin pathway in mouse embryonic stem cells treated with four GSK3 inhibitors. BMC Res. Notes *7*, 273.

Qie, S., and Diehl, J.A. (2016). Cyclin D1, cancer progression, and opportunities in cancer treatment. J. Mol. Med. *94*, 1313–1326.

Rao, J., Pfeiffer, M.J., Frank, S., Adachi, K., Piccini, I., Quaranta, R., Araúzo-Bravo, M., Schwarz, J., Schade, D., Leidel, S., et al. (2015). Stepwise clearance of repressive roadblocks drives cardiac induction in human ESCs. Cell Stem Cell *18*, 341–353.

Sato, N., Meijer, L., Skaltsounis, L., Greengard, P., and Brivanlou, A.H. (2004). Maintenance of pluripotency in human and mouse embryonic stem cells through activation of Wnt signaling by a pharmacological GSK-3-specific inhibitor. Nat. Med. *10*, 55–63.

Sepac, A., Si-Tayeb, K., Sedlic, F., Barrett, S., Canfield, S., Duncan, S.A., Bosnjak, Z.J., and Lough, J.W. (2012). Comparison of cardiomyogenic potential among human ESC and iPSC lines. Cell Transpl. *21*, 2523–2530.

Shy, B.R., Wu, C.I., Khramtsova, G.F., Zhang, J.Y., Olopade, O.I., Goss, K.H., and Merrill, B.J. (2013). Regulation of Tcf7l1 DNA binding and protein stability as principal mechanisms of Wnt/β-catenin signaling. Cell Rep. *4*, 1–9.

Siller, R., Greenhough, S., Naumovska, E., and Sullivan, G.J. (2015). Small-molecule-driven hepatocyte differentiation of human pluripotent stem cells. Stem Cell Reports *4*, 939–952.

Tan, J.Y., Sriram, G., Rufaihah, A.J., Neoh, K.G., and Cao, T. (2013). Efficient derivation of lateral plate and paraxial mesoderm subtypes from human embryonic stem cells through GSKi-mediated differentiation. Stem Cells Dev. *22*, 1893–1906.

Tighe, A., Ray-Sinha, A., Staples, O.D., and Taylor, S.S. (2007). GSK-3 inhibitors induce chromosome instability. BMC Cell Biol. *8*, 34.

Vitale, I., Manic, G., De Maria, R., Kroemer, G., and Galluzzi, L. (2017). DNA damage in stem cells. Mol. Cell *66*, 306–319.

Ye, S., Tan, L., Yang, R., Fang, B., Qu, S., Schulze, E.N., Song, H., Ying, Q., and Li, P. (2012). Pleiotropy of glycogen synthase kinase-3 inhibition by CHIR99021 promotes self-renewal of embryonic stem cells from refractory mouse strains. PLoS One *7*, e35892.

Yi, F., Pereira, L., Hoffman, J.A., Shy, B.R., Yuen, C.M., Liu, D.R., and Merrill, B.J. (2011). Opposing effects of Tcf3 and Tcf1 control Wnt stimulation of embryonic stem cell self-renewal. Nat. Cell Biol. *13*, 762–770.

Yoshino, Y., and Ishioka, C. (2015). Inhibition of glycogen synthase kinase-3 beta induces apoptosis and mitotic catastrophe by disrupting centrosome regulation in cancer cells. Sci. Rep. *5*, 13249.

Stem Cell Reports, Volume *10*

Supplemental Information

Unraveling the Inconsistencies of Cardiac Differentiation Efficiency Induced by the GSK3β Inhibitor CHIR99021 in Human Pluripotent Stem Cells

Filip Laco, Tsung Liang Woo, Qixing Zhong, Radoslaw Szmyd, Sherwin Ting, Fahima Jaleel Khan, Christina L.L. Chai, Shaul Reuveny, Allen Chen, and Steve Oh

Supplemental Experimental Procedures

Work flow, method and analyses of single embryoid body cardiac differentiation

We established a high throughput single embryoid body screening method with a cardiac reporter cell line HES3 NKX2-5$^{eGFP/w}$ to measure embryoid body formation, cell growth, cell cycle, cytotoxicity, mesoderm to cardiac differentiation and cellular signalling pathway of Wnt/beta-catenin. Using this system we investigated the effect of the dose and induction timing of GSK3β inhibitor CHIR and application of subsequent Wnt inhibitors on the process of mesoderm and cardiomyocyte differentiation. Moreover, the high throughput system enabled the investigation of the effect of different cell culture condition such as the initial cell culture densities and different cell lines during the embryoid body formation and differentiation process (Figure S7A). Critical findings were confirmed and repeated with a panel of 11 hPSC lines in monolayer culture systems.

In our workflow we evaluated monolayer cell cultures of human embryonic reporter stem cells line HES3 NKX2-5$^{eGFP/w}$ for pluripotency, viability, cell culture density and cell cycle. The cell cultures were dissociated and force aggregated to form single EBs. Several attempts to generate EBs by simply force aggregation of suspended cells failed; addition of Rock inhibitor Y27632 allowed HES3 to form aggregates but the resulting EBs were brittle and did not differentiate to cardiomyocytes (Figure S7B). A combination of CHIR, Y27632 and extracellular matrix (ECM) substrates such as Matrigel® was able to improve single EB formation (Figure S7B). We optimised time and dose concentration of HES3 derived cardiomyocytes and were able to show that single induction of CHIR induced cardiac differentiation efficiency of >85% Nkx2-5 expression (Figure S7C, Table 1S). The small molecule Wnt inhibitors were found not to be essential as their Wnt inhibitory function could be replaced by timely CHIR depletion. However, our full data set showed that small molecules inhibitors such as IWR-1 increase significantly the NKX-2.5 expression when CHIR was applied at higher concentration or for extended time course (Table S1). In addition, timely induction of IWR-1 was essential for the cardiac differentiation efficiency of certain cell lines in monolayer experiments (e.g. 4skin, DF6) which followed Lian et al. 2011 method (Table S2). A standard protocol was applied with an initial supplementation of Matrigel® (50mg/ml), 5µM Y27631 and 6 µM CHIR for 24 h to induce a robust EB formation and differentiation (Figure S7D). During the differentiation process, EBs were lysed at early and late developmental stages (Day 0-6) and protein levels were quantitatively analysed by antibody detection via capillary electrophoresis western blots (Figure S7A).

EB size was evaluated by measuring the whole EB area (Figures S7E-F). The measured area was found to correlate with the cell number count of dissociated EBs at 10100 cells/0.1mm^2 (Figure S7G).

GFP/NKX2-5 expression is an essential marker for progenitor and early cardiomyocytes (Lyons et al., 1995). Fluorescence microscope assisted screens of EBs were able to identify and calculate the portion of the HES3 NKX2-5$^{eGFP/w}$ GFP area of the whole EB area (Figures S7E-F). Flow cytometry analysis showed that HES3 NKX2-5$^{eGFP/w}$ GFP staining was found to co-stained with Troponin T (Figure S7G). Thus cardiac differentiation efficiency of HES3 NKX2-5$^{eGFP/w}$ reporter cell line was evaluated from day 6 by imaging the GFP expression area of the EB (GFP area). GFP image analyses were found to correlate with GFP flow cytometry of single cell dissociated EBs (r^2= 0.91) (Figure S7G).

Cell Culture

Embryonic stem cell lines HES3 and HES3$^{NKX2-5eGFP/w}$ (reporter cell line for cardiac differentiation was kindly provided by David Elliot Monash University Australia), WA01 (H1), WA07 (H7), human induced pluripotent stem cell (iPS) lines iPS(IMR90)-1 (IMR-90), iPS(Foreskin)-1 (4Skin), iPS-DF6-9-9T (DF6), IISH1i-BM1 (BM1),and IISH3i-CB6 (CB6) (WiCell Research Institute), Donor 5 and X.13 (derived at the Institute of Molecular and Cell Biology, Singapore, kindly provided by Dr. Jonathan Loh) and FR201 and FR202 (derived at the Bioprocessing Technology Institute, Singapore, kindly provided by Dr. Alan Lam) were cultured on Geltrex® coated tissue culture plates. All cell lines used for Aggrewell® or Single EB experiments were cultured in Essential 8™ media (Life Technologies). All cell lines used in monolayer and microcarrier experiments were cultured in mTeSR™1 (Stemcell Technologies). HES3 and HES3$^{NKX2-5eGFP/w}$ were cultured in both media types for comparison in different

differentiation protocols. Media was refreshed daily and cultures were passaged mechanically with STEMPRO® EZPassage tool (Life Technologies) every 3–6 days at about 70% cell plate confluency. Cell cultures were incubated at 37°C in a humidified atmosphere with 5% CO2.

Single and Aggrewell® EB formation

Protocol was adapted from (Ng et al., 2005) and the Stemcell Technologies™ manual for Aggrewell® EB formation. Briefly: HES3$^{NKX2\text{-}5eGFP/w}$ cells were dissociated with TrypLE (Invitrogen) and seeded at 1.5 × 10^4 cells/well in ultra-low attachment 96-well clear round bottom plates (Corning) in bSFS medium 150µl/Well. bSFS composition (Ting et al., 2013) : DMEM supplemented with 2mML-glutamine, 0.182 mM sodium pyruvate, 1% non-essential amino acids, 5.6 mg/l transferrin (all from Life Technologies), 0.1 mM β-mercaptoethanol (Sigma), 20 µg/L sodium selenite (Sigma), 0.25% (w/vol) Bovine Serum Albumin (MP) and 0.25% (w/v) Hysoy (Sheffield Bioscience)] HES3$^{NKX2\text{-}5eGFP/w}$, H1, H7, IMR-90, DF6 and CB6 cells were seeded at 4.5× 10^6 cells/well in Aggrewell® plates (Corning) in bSFS medium 2.5ml/Well. Cells were forced to aggregate by spinning down at 800 rpm for 1min and incubated for 24 h with 1.5-12 µM CHIR99021 (Selleckchem), 5 µM Y27632 (Selleckchem) and 50 µg/ml Matrigel® (BD, UK) at 37°C and 5% CO2 to allow EB formation. Media changes and initial cell seeding in 96-well plates was assisted by liquid handling unit Viaflow Assist (Integra, US) for volume and cell seeding accuracy.

Microcarrier HES3 Culture and Cardiac Differentiation

Protocol is described by (Ting et al., 2014) and available at http://dx.doi.org/10.1016/j.scr.2014.06.002

Fluorescence microscopy assisted EB screening

Dual images of phase contrast and fluorescence for green fluorescence protein detection (GFP) were taken of each EB in 96-wells U-bottom plates with an inverted Nikon Eclipse Ti-E microscope b/w camera. The 96-well plate was mounted on a fully automated movable stage which allowed precise positioning and focusing of each well and EB (Nikon). The culture plates were kept at 37˚C with 5% CO_2 via an on-stage incubator. A full screen of 96-wells took 3 min with our high speed processing and storage hardware (Nikon). Images were taken at a resolution of 1.62 µm/pixel at fixed settings for phase contrast imaging: light intensity 60%, exposure 2 ms, for fluorescence imaging: light intensity 29%, exposure 700 ms. Images were saved as .tif stack format for further processing and quantification with Image J®. Images were processed to quantify the GFP size of HES-3 *NKX2-5*$^{eGFP/w}$, and EB size with Image J®. Briefly: Fluorescence and phase contrast images stacks were reduced to 8-bit greyscale, background subtracted, optionally inverted, processed to black/white images with image threshold. EB or GFP pixel area was calculated with Image J® particle analyser. Pixel area was calculated back to area (mm^2).The area was converted to the actual cell number my measuring the dissociated cell numbers of different sized EBs. A pixel area of 0.1 mm^2 = 10,100 Cells (±951 Cells). GFP percentage was calculated following the equation: GFP percentage = (GFP area)/(EB area). Only EBs between 0.08-0.2 mm^2 were used for GFP area calculations to eliminate technical measurement errors. 4-8 EBs were used as technical replicates per tested condition to allow for statistical analyses.

Flow Cytometry

Cells were harvested, and dissociated into single cells using TrypLE™ Express (Life Technologies) for 7–10 min in a heating block under constant shaking (Thermomixer comfort, Eppendorf) at 37°C,. Dissociated samples were pipetted through a 20 µm nylon mesh Multi Screen® (Millipore). Cells were fixed in Fix and Perm solution® medium A (Life Technologies) for 15 min at room temperature (RT). Cells were incubated with primary antibodies (Supplemental Table 3) in 1% bovine serum albumin (BSA) with 0.2% Triton X-100 in PBS for 30 min. After that cells were washed with blocking buffer 1% BSA in PBS, and incubated with a 1:500 dilution of Alexa Fluor 647 conjugated goat anti-mouse secondary antibody (Life Technologies), Alexa Fluor 488 conjugated goat anti-rabbit secondary antibody (Life Technologies) for 20 min in blocking buffer. Cells were washed with blocking buffer and analysed on a flow cytometer (GUAVA easy Cyte 8HT, Millipore) using standard filter sets for secondary antibodies and green fluorescence protein (GFP) of the HES3 $^{NKX2\text{-}5eGFP/w}$ cell line. The GFP expression of HES3

NKX2-5eGFP/w cells from single EB experiment were live analysed and did not require fixation and staining. Analyses were performed with Flowjo®.

Immunohistochemistry
Cultured cells were fixed with 4% paraformaldehyde and blocked for 1 h in 3% BSA/PBS. Primary antibodies (Table S3) were incubated overnight at 4 °C in 0.2% Triton X-100, 3% BSA/PBS. Thereafter, Alexa Fluor 488, 594 and 647 conjugated goat anti-mouse/rabbit secondary antibodies (Life Technologies) were incubated for 2 h at 1:500 dilution in 3% BSA/PBS. Cell imaging was performed with a Nikon eclipse *Ti*-E inverted fluorescence microscope. Pseudo colours were added for a better visualization of the cyto -antibody stains with Image J®.

Metabolic MTT assay
MTT (5 mg/ml) solution (Sigma) was added to dissociate 1.5×10^4 cells/well and differentiating EBs in 96-wells and incubated at 37 °C in a humidified atmosphere of 5% CO2 for 3 h, after which the MTT desorb solution (acidified isopropanol) was added to each well. The plate was shaken for 15 min to dissolve the formazan. The absorbance was measured at 550–570 nm in an Infinite® M200 (Tecan) microplate reader.

Metabolite analyses
Glucose, glutamine, lactate, and ammonia concentrations were analysed using Bioprofile 100 plus (NOVA). pH was measured with a pH meter (Camlab). Methods for analyses and calculating specific metabolites consumption / production rates are described previously by (Chen et al., 2010).

Cell Cycle analyses
Cell cycle analyses were performed by using NucleoCounter NC-3000 (Chemometec) according to the manufacturer's handbook. Briefly: Cells and EBs were dissociated with TrypLE™ Express (Life Technologies) and fixed with 70% ethanol in PBS for 2 h at 2°C followed by incubation of 1 µg/ml DAPI (Sigma), 0.1% Trition-X (Sigma) in PBS for 5 min at 37 °C. DAPI count, size and intensity staining was measured with a NucleoCounter NC-3000 (Chemometec). Cell cycle plots were analysed with FlexiCyte™ software (Chemometec) to calculate cell cycle states in percentage of sub-G1, G1-, S-, G2/M-phase.

Cell Cycle arrest/synchronization
hPSCS were arrested at the G1/S phase by adding 2 mM Thymidine (Sigma) for 18 h in culture media under cell culture condition. G2 arrest was induced by adding 10ng/ml Nocodazole (Sigma) for 12h in culture media under cell culture condition. After the incubation cells were washed with PBS prior further culture or differentiation.

Cell Count
Cell number and cell viability was determined by the nuclei count method with DAPI and Acridine Orange using NucleoCounter NC-3000 (Chemometec) according to the manufacturer's handbook.

Cell lyses and protein quantification assay
Cytosol and nuclear protein fractions were collected and lysed with NE-PER Nuclear and Cytoplasmic Protein Extraction Reagents (Life Technology) with addition of Halt Protease & Phosphatase Inhibitor cocktail (Life Technology) and 1 mM PMSF according to the manufacturer's protocol. Whole protein was extracted with lysis buffer CST (Cell Signaling Technology) containing 1mM phenylmethylsulfonyl fluoride (PMSF) (Sigma) and 1 x Halt Protease & Phosphatase Inhibitor cocktail (Life Technology). Cell lysis solutions were incubated for 15min at 4°C with periodically shaking followed by 10 min spin down at 16000rpm in a microcentrifuge. The supernatant was collected for protein quantification, capillary western blot and stored at -80°C. The protein content of cell lysis solutions and albumin protein standards (Life Technology) were analysed with a colorimetic DC Protein Assay (Bio-rad) according to the manufacture protocol. Light absorbance was measured against a wavelength of 750nm with an Infinite® M200 (Tecan) microplate reader. Protein mass (mg/ml) of the lysis solution was calculated from the albumin protein standards curve.

Capillary western blot and protein antibody Immuno detection, quantification and statistical analyses

Protein identification and quantification was carried out using a Simple Western system (Peggy Sue™, Protein Simple, R&D), a fully automated western capillary based protein separation and detection system (Harris, 2015). All materials with the exception of the primary antibodies were purchased from Protein Simple. The manufactures protocol for size separation and machine settings for size separation (12-230kD) were applied (www.proteinsimple.com). Briefly: Proteins lysates with 1 mg/ml protein content were denaturised with DTT and SDS at 95°C. Samples were size separated through a gel matrix that allow protein identification between 12-230kD. The separated proteins were immobilized to the capillary wall by photo-activation. Followed by blocking, primary antibody (Supplementary Table 3) and secondary horseradish peroxidase conjugated antibody incubation. Protein detection was initiated by induction of Luminol/Peroxidase substrate. The resulting chemiluminescent was measured with a photosensitive camera over a 15 min incubation time. The protein band identification and the quantification of the chemiluminescence were performed with Compass™ software (Proteinsimple, R&D) according to the manufactures protocol. Chemiluminescence values were transformed into western bot image data and line/bar charts with Compass™ software and Excel®. Protein samples were grouped from individual EB and monolayer experiments. A minimum of 48 EBs were used to include a wide range of experimental variations. Protein samples were prepared with up to 3 biological replicates for capillary electrophoresis and antibody detection. A maximum of 12 experimental sample conditions were run simultaneously in individual capillaries. 2-4 Antibodies were detected per sample condition in multiplex analyses. Up to 8 runs per biological sample were performed. Differences between the capillaries and subsequent runs were monitored by an internal system controls and loading controls such as GAPDH, GSK3b, lamin A/C and actin. Relative expression values were obtained by normalizing against loading controls and internal standard across all 8 columns. The protein quantification standard deviation was a found to be 10-25% across all capillaries.

Quantitative RT-PCR

Total RNAwas isolated using the RNeasy Mini Kit (Qiagen) from EBs following the supplier's protocol. Reverse transcription was carried out with 1 μg total RNA using SuperScript III (Invitrogen). Real-time PCR was performed applying a standard two-step amplification protocol on an ABI 7500 system (Applied Biosystem) to detect mRNA expression (primer sequences are, Dkk-1F: 5′ ACCCAGGCTCTGCAGTCA 3′, Dkk-1R: 5′ CCTGCAGGCGAGACAGAT 3′; MESP-1F: 5′ GACGTGCTGGCTCTGTTG 3′, MESP-1R: 5′ TGTCACTTGGGCTCCTCAG 3′, T-bra F: 5′ AATTTGGTCCAGCCTTGGAAT 3′, T-bra R: 5′ CGTTGCTCACAGACCACAG 3′). Relative expression values were obtained by normalizing $C\tau$ values of the tested genes to the $C\tau$ values of the house keeping gene GAPDH using the $\Delta\Delta C\tau$ method (Schmittgen and Livak, 2008).

Statistical Analyses

All experiments including EB area, GFP area/percentage, flow cytometry, immunostaining quantification, cell cycle, qPCR, cell numbers and metabolic data were performed at least 3 times. The number of experiments is indicated at the figure caption as n=experiments in independent experiments. All data are expressed as the mean ±SEM using the statistical software GraphPad Prism®, version 4.1. Comparisons of two data sets were statistically analysed with Student's t-test. Multiple comparisons between more than three groups were performed using analysis of variance with one-way Anova multiple comparison tests. A p value is shown when the difference between compared groups is significant. The statistical significance was indicated by *= ($p < 0.05$). The capillary western blot data provides information from a single experiment. Each experiment was analysed individually and statistics were performed from biological replicates and technical replicates system as described earlier. Key experiments leading to general claims were performed with at least 2 additional cell lines and/or with 2 alternative methods, which were included in the supplemental figures.

Supplemental References

Chen, X., Chen, A., Woo, T.L., Choo, A.B.., Reuveny, S., Oh, S.K.., 2010. Investigations into the metabolism of 2D colony and suspended microcarrier cultures of human embryonic stem cells in serum free media. Stem Cells Dev. 19.

Harris, V.M., 2015. Protein Detection by Simple WesternTM Analysis, in: Kurien, B.T., Scofield, R.H. (Eds.), Western Blotting: Methods and Protocols. Springer New York, New York, NY, pp. 465–468. doi:10.1007/978-1-4939-2694-7_47

Lyons, I., Parsons, L.M., Hartley, L., Li, R., Andrews, J.E., Robb, L., Harvey, R.P., 1995. Myogenic and morphogenetic defects in the heart tubes of murine embryos lacking the homeo box gene Nkx2-5. Genes Dev. 9, 1654–1666. doi:10.1101/gad.9.13.1654

Ng, E.S., Davis, R.P., Azzola, L., Stanley, E.G., Elefanty, A.G., 2005. Brief report Forced aggregation of defined numbers of human embryonic stem cells into embryoid bodies fosters robust , reproducible hematopoietic differentiation. Differentiation 106, 1601–1603. doi:10.1182/blood-2005-03-0987.Supported

Schmittgen, T., Livak, K., 2008. Analyzing real-time PCR data by the comparative C(T) method. Nat. Protoc. 3, 1101–8.

Ting, S., Chen, A., Reuveny, S., Oh, S., 2014. An intermittent rocking platform for integrated expansion and differentiation of human pluripotent stem cells to cardiomyocytes in suspended microcarrier cultures. Stem Cell Res. 13, 202–213. doi:10.1016/j.scr.2014.06.002

Ting, S., Lecina, M., Chan, Y.-C., Tse, H.F., Reuveny, S., Oh, S.K., 2013. Nutrient supplemented serum-free medium increases cardiomyogenesis efficiency of human pluripotent stem cells. World J. Stem Cells 5, 86–97. doi:10.4252/wjsc.v5.i3.86

Supplemental Figure S1

A

Images HES3 (Day 0)	Culture Confluence	Cell Number	viability	sub-G1	G1	S	G2/M	pH	qGlucose (g/$1e^6$cells/Day)	qGlutamin (mmol/$1e^6$cells/Day)	qLactate (g/$1e^6$cells/Day)	Yield (Lac/Glc)
a	40%	1.62E+06	90%	6%	51%	21%	21%	7.07	0.56	0.46	1.00	1.78
b	60%	2.94E+06	93%	5%	58%	17%	19%	6.96	0.40	0.23	0.62	1.54
c	80%	3.06E+06	91%	3%	59%	17%	19%	6.88	0.42	0.20	0.65	1.55
d	100%	4.06E+06	92%	4%	59%	19%	16%	6.88	0.29	0.12	0.48	1.64
Medium	mTeSR							7.2	2.5	3	0	

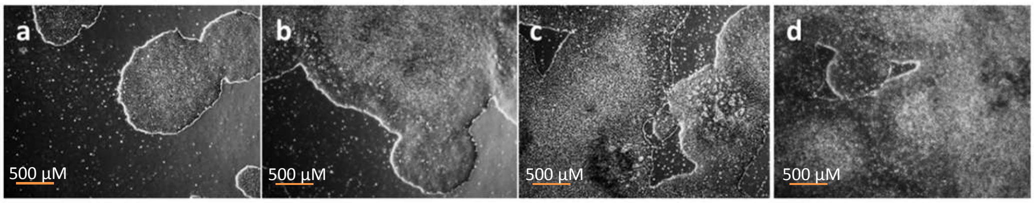

Images after 24h induction (10µM CHIR99021)	Culture Confluence	Cell Number	viability	sub-G1	G1	S	G2/M	pH	qGlucose (g/$1e^6$cells/Day)	qGlutamin (mmol/$1e^6$cells/Day)	qLactate (g/$1e^6$cells/Day)	Yield (Lac/Glc)
e	50%	2.66E+06	84%	3%	24%	19%	53%	8.06	0.36	0.08	0.47	1.28
f	70%	3.64E+06	86%	4%	19%	28%	48%	7.97	0.28	0.04	0.39	1.39
g	75%	2.96E+06	83%	6%	18%	28%	48%	7.99	0.35	0.06	0.50	1.41
h	70%	2.06E+06	79%	6%	18%	18%	56%	7.95	0.51	0.05	0.70	1.37
Medium	bSFS+10µM CHIR99021							8.2	4.5	1	0	

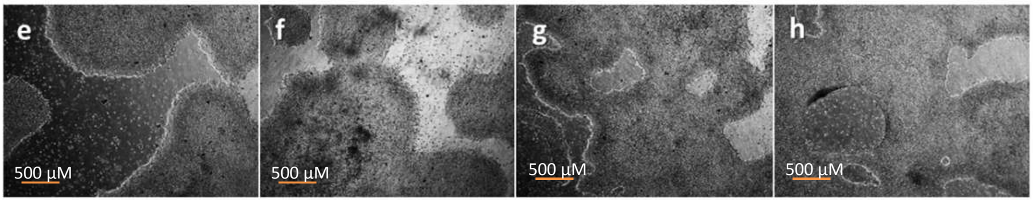

B

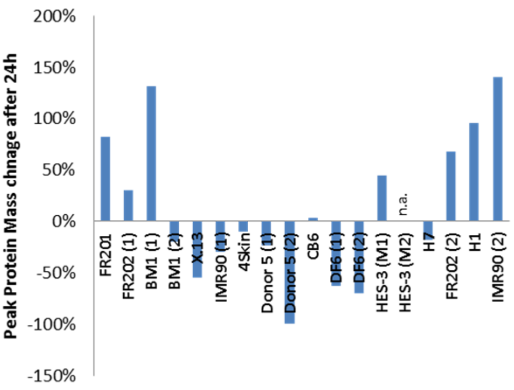

C

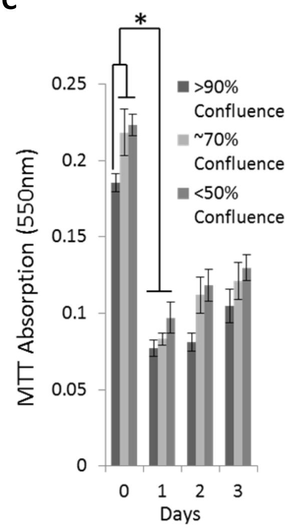

D

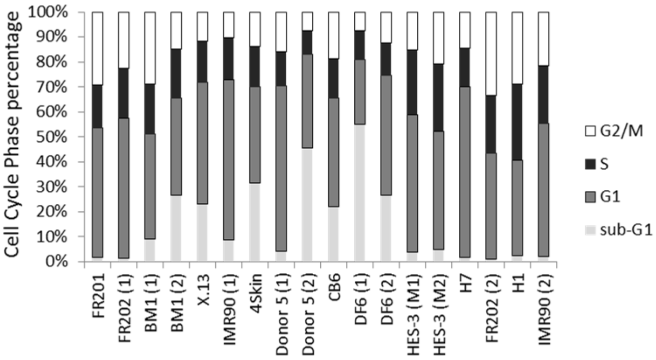

Supplemental Figure S1

Supplemental Figure S1: (A) Cell Cycle, Cell number, viability, pH and metabolite (Glucose, Glutamine and Lactate) measurements and microscope phase contrast images of 40-100% confluent HES3 cells in monolayer culture on day 0 with mTeSR^{TM}1 and after 1 day of treatment with 10µM CHIR in bSFS medium. (B) Maximal change of protein mass percentage after 24h treatment with 4-12 µM CHIR (C) Cell metabolism and growth (MTT absorbance) kinetics of EBs generated from passage 10 with 50%, 70% and 90% culture confluency induced with 6 µM CHIR for 24 h (n=3 ind. ex.). (D) Graphical illustration of cell cycle profiles of 11 hPSC lines.

Supplemental Figure S2

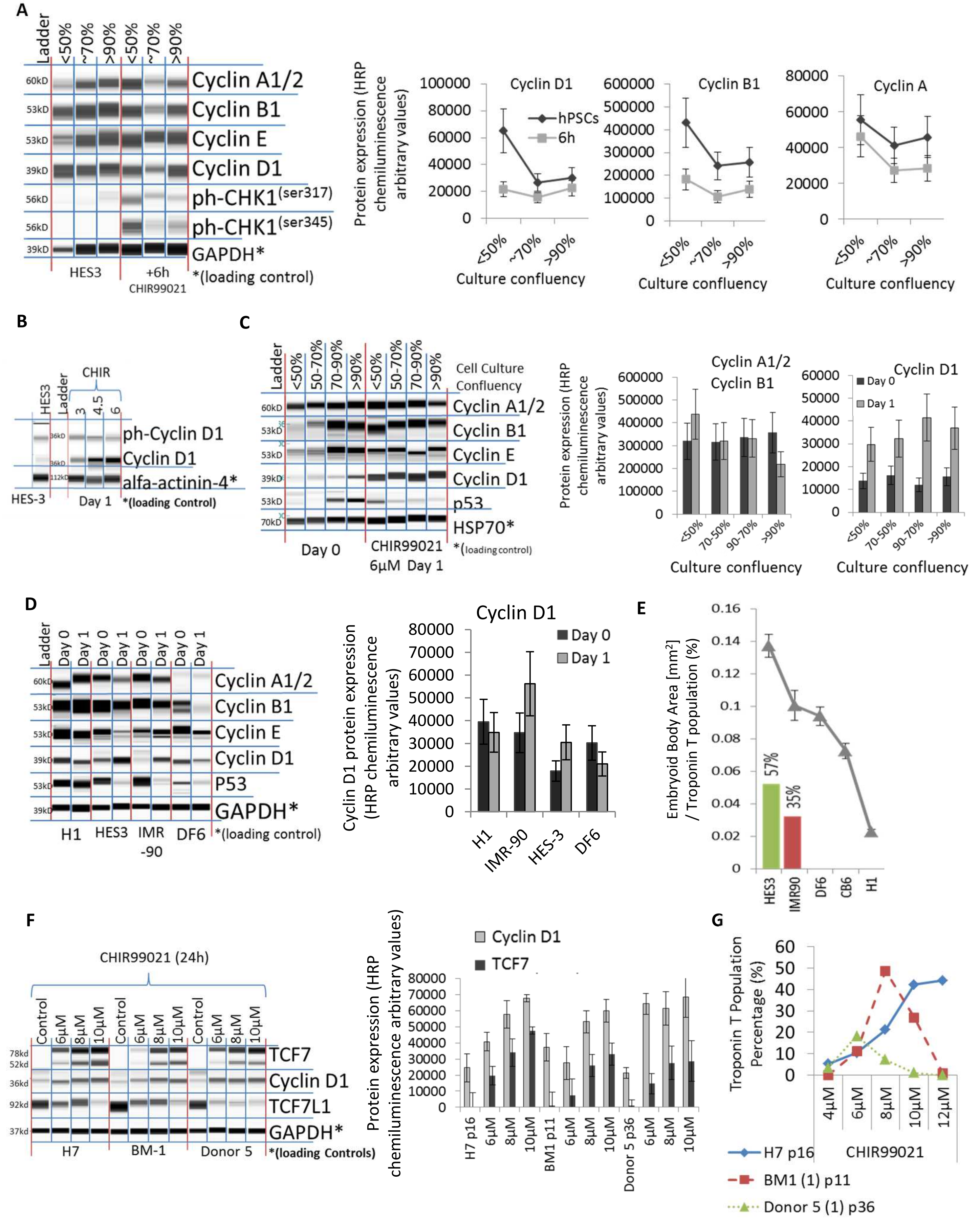

Supplemental Figure S2

Supplemental Figure S2: (A) Whole cell protein blot and quantitative analyses of hPSCs cultured at 50%, 70% and 90% culture confluency after treatment with 12 µM CHIR. (B) Whole cell protein blot of HES3 EB cell cultures after CHIR induction. Analyses showed dose dependency of Cyclin D1 protein expression. (C) Whole cell protein blot and quantitative analyses of HES3 cells cultured at <50%, 50-70%, 70-90%, >90% cell culture confluency and HES3 EBs after CHIR induction. (D) Whole cell protein blot and quantitative analyses of hPSCs and hPSC EBs after CHIR induction (6 µM H1, HES-3, IMR-90 and 3 µM DF6). (E) EB area size of 5 cell lines after 24 h treatment with CHIR (6 µM H1, HES-3, IMR-90 and 3 µM DF6) and their Troponin T population expression (%) expression on day 14. (F) Whole cell protein blot and quantitative analyses of H7, BM1 and Donor 5 cells after CHIR induction. Analyses showed dose dependency of TCF7 and Cyclin D1 protein expression levels. (G) Troponin T expression on day 14 of 3 H7, BM1 and Donor 5 cells after CHIR induction for 24h.

Supplemental Figure S3

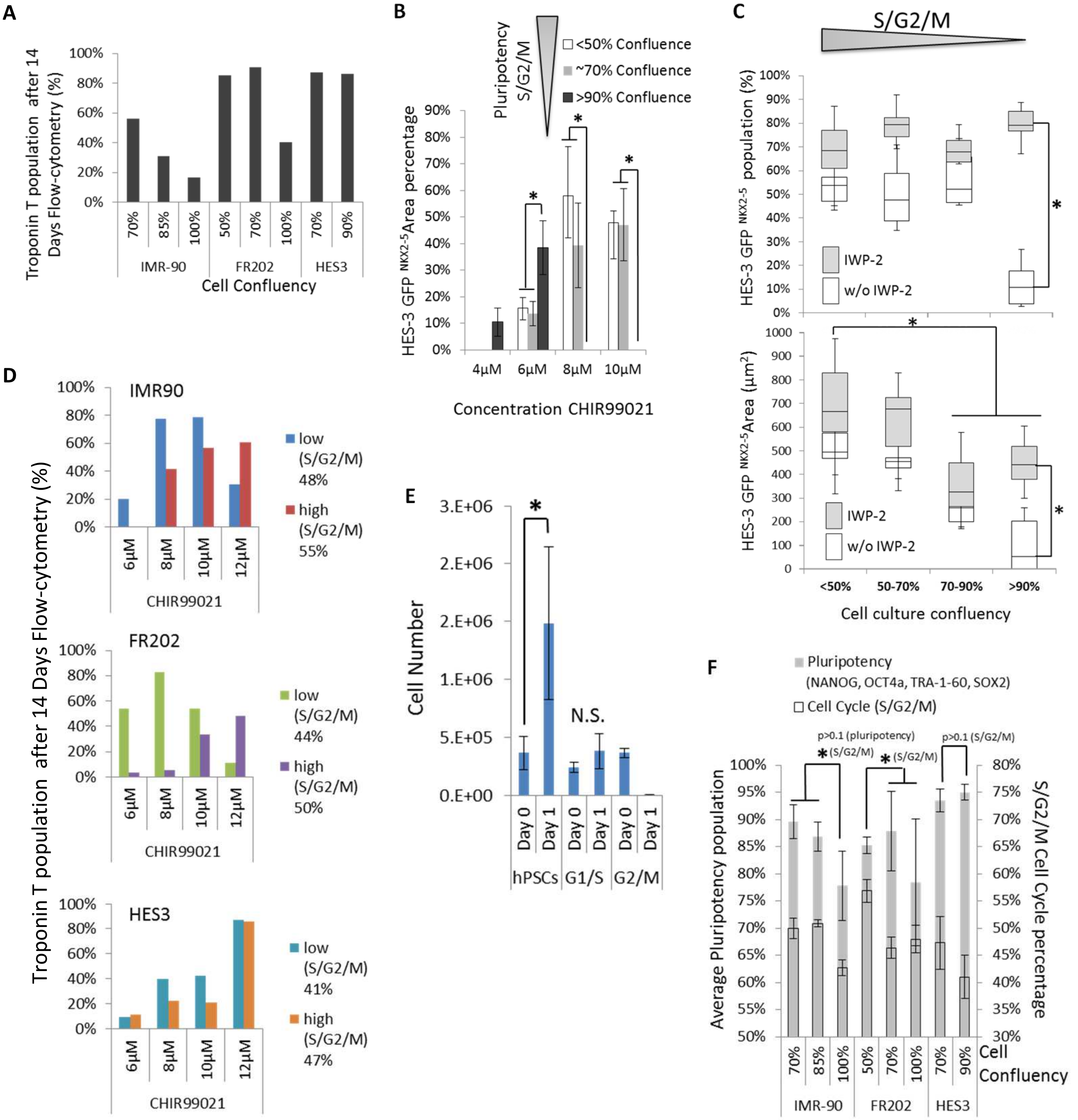

Supplemental Figure S3: (A) Troponin T cell population (%) of monolayer hPSC lines (IMR90, FR202 and HES3) on day 14 cultured at 50%-100% culture confluency and differentiated with 12 µM CHIR induction for 24 h (B) EB GFP $^{NKX2-5}$ expression on day 11 of HES3 cells cultured at <50%, 70%, 90% cell culture confluency of low to high S/G2/M cell cycle profiles and differentiated with 4-10 µM CHIR induction for 24h (n=4). (C) EB GFP $^{NKX2-5}$ flow cytometry population and total GFP $^{NKX2-5}$ positive EB area of HES3 cells cultured at <50%, 50-70%, 70-90%, >90% cell culture confluency and differentiated with 6 µM CHIR induction for 24 h and 5 µM IWP-2 induction on day 3 (n=8). (D) Troponin T population (%) on day 14 of monolayer hPSC lines (IMR90, FR202 and HES3) cultured at low 50-70% to high >90% cell culture confluency with a 6% difference in S/G2/M cell cycle profile and differentiated with CHIR induction for 24h. (E) Average cell number of pooled HES3, FR202 and IMR90 cell lines after cell cycle arrest at G1/S and G2 and 12 µM CHIR induction (F) Average pluripotency flow cytometry population (%) of NANOG, OCT4a, TRA-1-60 and SOX2 and the percentage of the S/G2/M cell cycle profile of IMR90, FR202 and HES3 cells cultured at variable cell densities (n=3).

Supplemental Figure S4

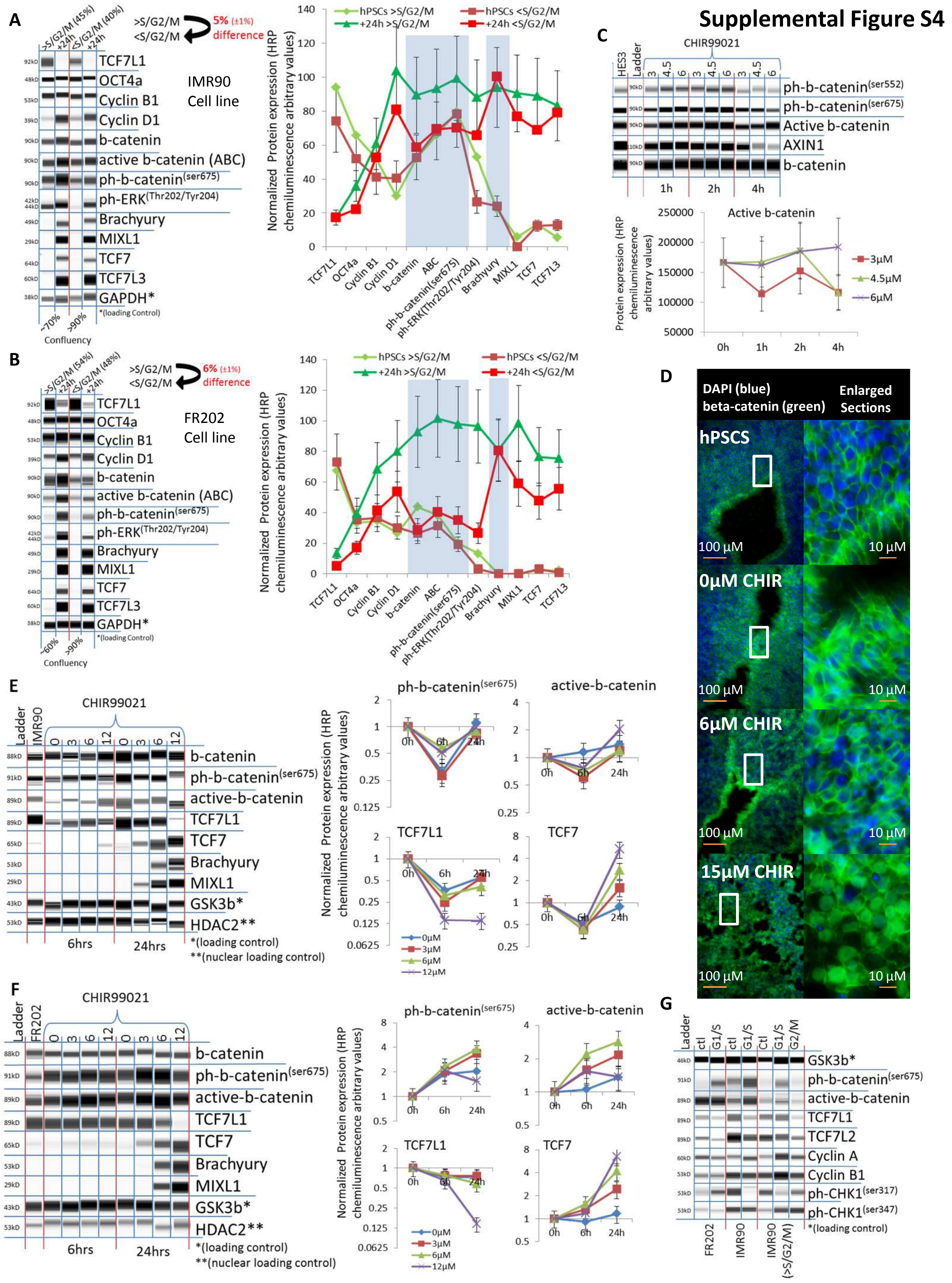

Supplemental Figure S4

Supplemental Figure S4: (A) Whole cell protein blot and quantitative analyses of IMR90 cells cultured at >90% culture confluency with a low S/G2/M (40%) cycle profile and ~70% culture confluency cell with a high S/G2/M (45%) cycle profile after 10 μM CHIR induction. High culture density with a low S/G2/M cell cycle poorly expressed catenin proteins. T-Brachyury was not affected by cell culture density and S/G2/M cell cycle profile differences. (B) Whole cell protein blot and quantitative analyses of FR202 cells cultured at >90% culture confluency with a low S/G2/M S/G2/M (48%) cycle profile , >90% culture confluency and ~60% culture confluency cell with a high S/G2/M (54%) cycle profile after 12 μM CHIR induction. High culture density with a low S/G2/M cell cycle poorly expressed catenin proteins. T-Brachyury was not affected by cell culture density and S/G2/M cell cycle profile differences. (C) Whole cell protein blot and quantitative analyses of HES3 and HES3 EBs after CHIR induction. (D) Fluorescence microscopy of immuno-cytochemistry stained FR202 cells against active beta-catenin (green) and DAPI (blue) after 24 h treatment with CHIR. DAPI co-staining with beta-catenin indicates nuclear translocation. (E) Nuclear fraction protein blot and quantitative analyses of IMR90 cells after CHIR induction. Analyses shows dose dependency of TCF proteins. (F) Nuclear fraction protein blot and quantitative analyses of FR202 cells after CHIR induction. Analyses shows dose dependency of TCF and catenin proteins. (G) Whole cell protein blot of Wnt signalling, and Cyclin proteins of the FR202 (S/G2/M=46%) and IMR90 (S/G2/M=43%) and (S/G2/M=51%) cell lines after cell cycle arrest.

Supplemental Figure S5

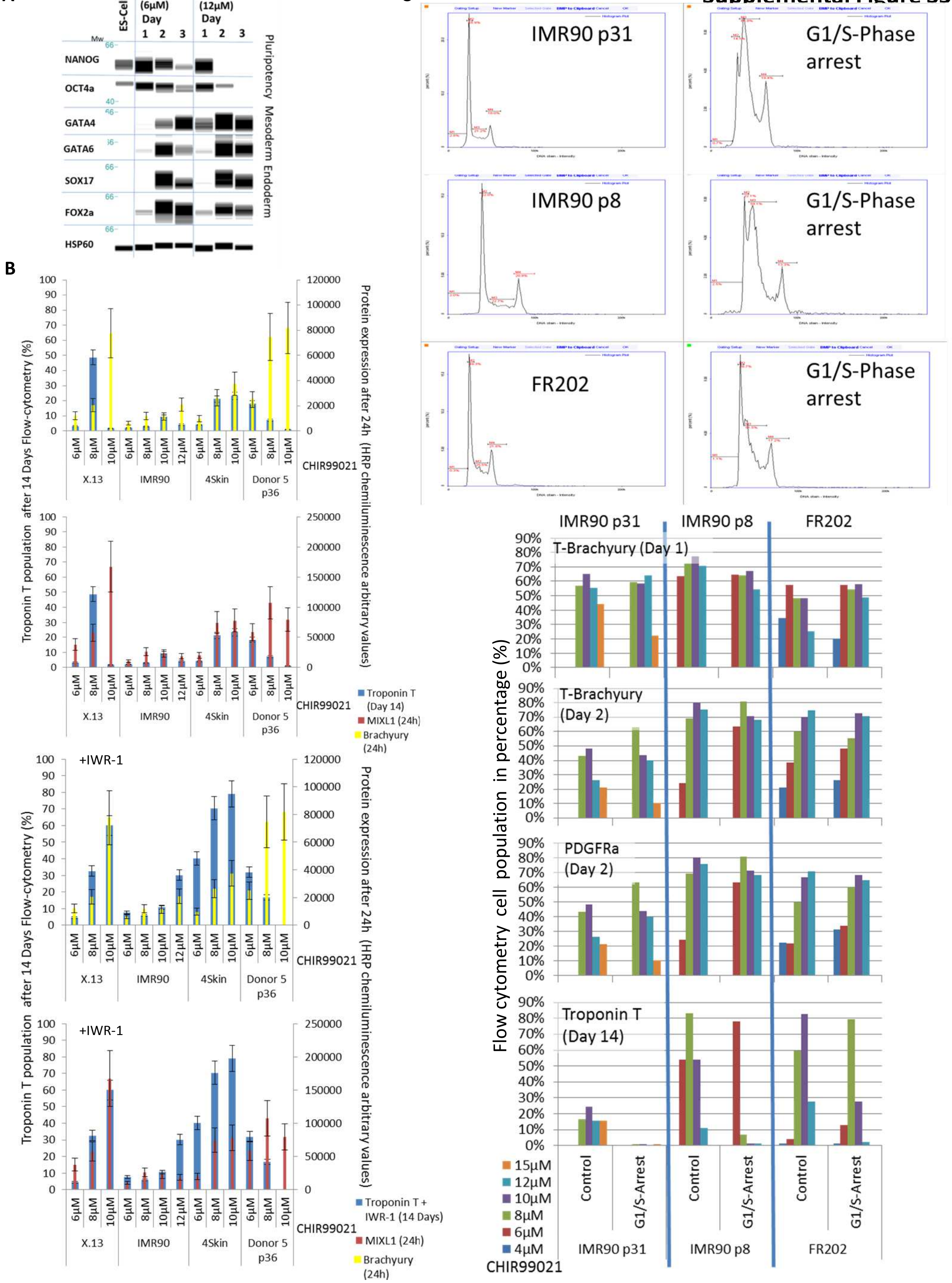

Supplemental Figure S5

Supplemental Figure S5: (A) Whole cell protein blot of pluripotency signalling proteins (NANOG, OCT4), and Endo-Mesoderm markers (GATA4/6, SOX17, FOX2a) expression of microcarrier attached IMR90 aggregates treated for 24h with CHIR. (B) HRP chemiluminescence expression analyses of T-Brachyury and MIXL1 proteins of X.13, IMR90, 4Skin and Donor 5 cells after CHIR induction, and flow cytometry population (%) of Troponin expressing cells on day 14. (C) Cell cycle profiles of IMR90 p31 and p8 and FR202 with and without G1/S arrest. Flow cytometry population (%) of T-Brachyury on day 1-2, PDGFRa on day 2 and Troponin T on day 14 of IMR90 p31, p8 and FR202 cells after G1/S cell cycle arrested and treatment with 4-15 μM CHIR for 24 h.

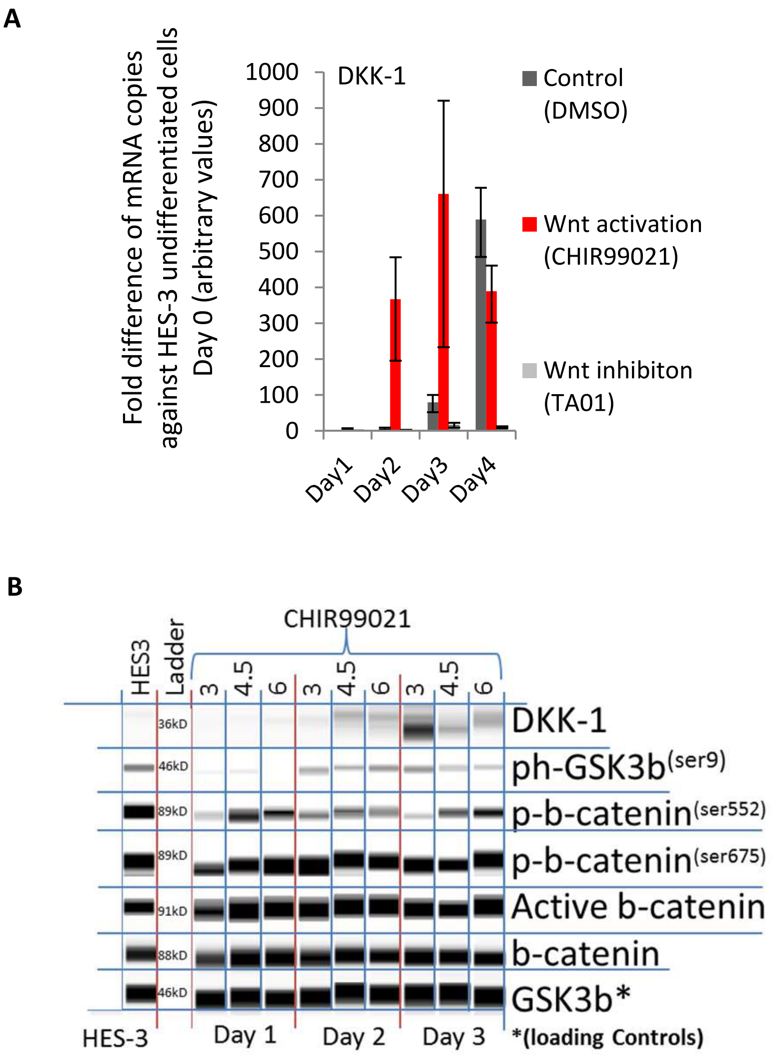

Supplemental Figure S6: (A) qPCR analyses of Wnt inhibitor DKK-1 with 5µM TA-01 (Wnt inhibition) and 3 µM CHIR (h). Measurements are compared to HES3 and displayed as fold increase/decrease over HES3 control, where 1 is equal to the HES3 control (n=3) (B) Whole cell protein blot of DKK-1/GSK3b/b-catenin signalling proteins of HES3 cells and EBs. b-catenin$^{(ser522,\ ser675)}$ levels declined after treatment with 3 µM CHIR for 24 h. All b-catenin levels were not significantly increased after CHIR induction. The increase of DKK-1 and ph-GSK on day 2/3 indicate Wnt inhibition.

Supplemental Figure S7

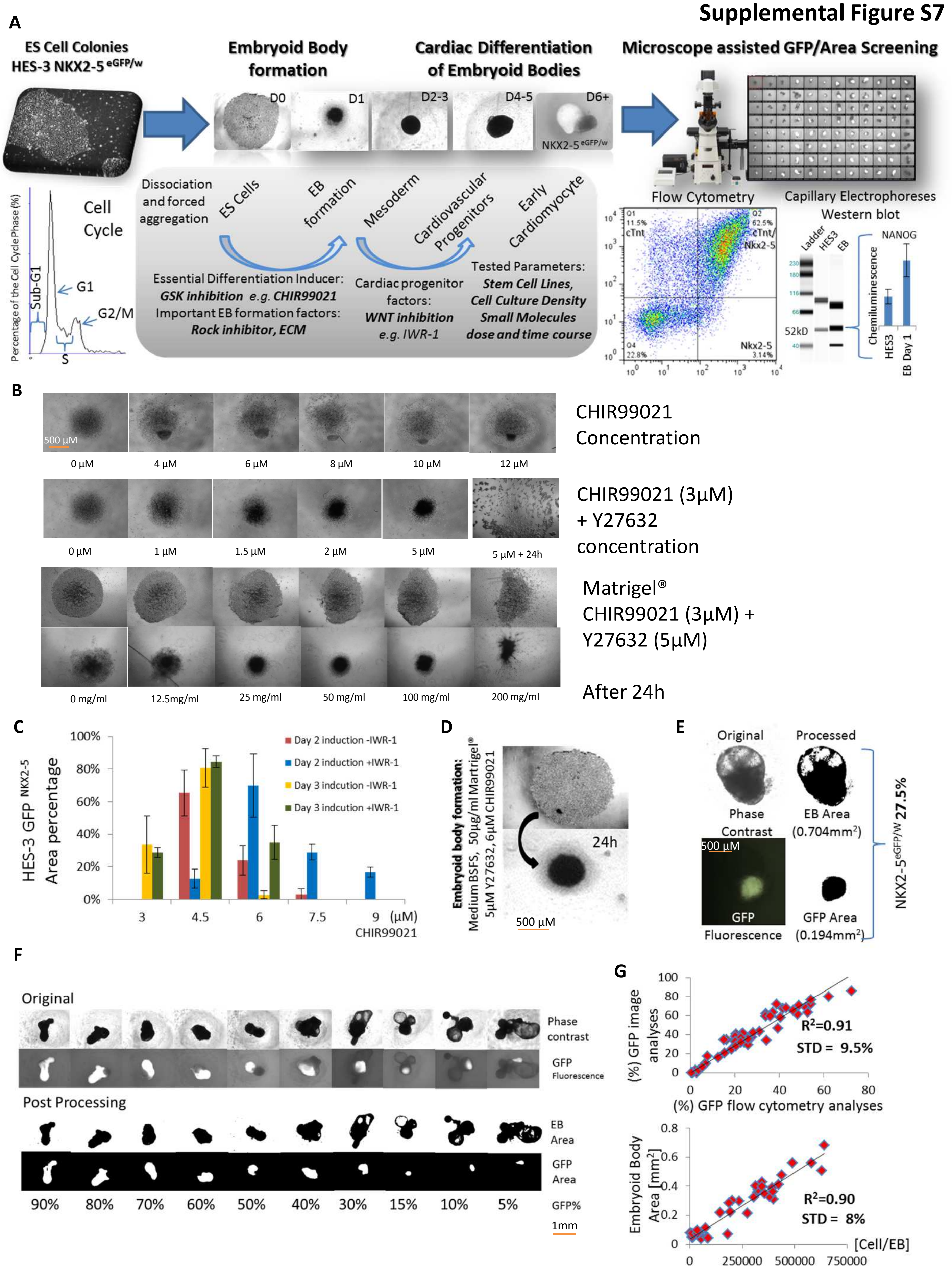

Supplemental Figure S7

Supplemental Figure S7: (A) Workflow: HES3 $^{NKX2\text{-}5eGFP}$ reporter cell line cultures were analysed for cell cycle, cell number, cell culture density and pluripotency. Thereafter 15000 cells harvested from these cultures were seeded in 96-wells to form EB by forced aggregation with Rock inhibitor and ECM supplementation and in presence of CHIR inducer which initiate differentiation via Wnt regulation towards mesoderm and early cardiomyocytes. EB analyses included size (measurement of area), differentiation (measurement of GFP area, white area), flow cytometry, cell count and cell protein analyses with capillary electrophoresis western blot. (B) Microscope phase contrast images of forced aggregated EBs after treatment with CHIR and Y27632 for 24 h (top 2 image rows). Microscope phase contrast images of forced aggregated HES3 cells and EBs after 24 h with Geltrex® (bottom 2 image rows). (C) Percentage of GFP $^{NKX2\text{-}5}$ area of HES3 EBs on day 10 days after treatment with 3-9 µM CHIR for 24h and 2.5µM IWR-1 induction on day 2/3. (D) Example of EB and GFP area analyses with ImageJ® for GFP area differentiation percentage. (E) Phase contrast image of 15000 forced aggregated HES3 cells and after EB formation with standard method parameters of 50mg/ml Matrigel®, 6 µM CHIR and 5 µM Y27632. (F) Original: microscope phase contrast (top) of EB and fluorescence images of GFP expression (white). Post processing: EB and GFP area analyses with Image J for GFP area percentage. (G) Correlation between EB differentiation efficiency measured by flow cytometry and GFP area percentage measured by imaging and calculating the GFP/EB area (Top) (n=51, >204 EBs). Correlation between EB size evaluation by measurement of area by imaging and cell count of dissociated EBs (Bottom) (n=44, >176 EBs).

TABLE S1

Cardiac differentiation optimasation:

Media Change — Days: 0 | 1 | 2 | 3-5 | 6

BSFS* Media, 5µM Y27632, 50µg/ml Matrigel, 12500 hESCs/Well (120µl Volume)

Day 1: CHIR99021 1.5µM; Days 3-5: IWR-1 2.5µM; Day 6: Media Change to BSFS*

Day 0 condition	Analyses Microscopy Day 10: EB Area (mm²)	STD	GFP Area (mm²)	STD	GFP Area (%)	STD	Flow Cytometry Analyses Day 10: GFP Flow (%)	STD	Cell Count Cells/µl	STD	GFP Yield Cells/µl
CHIR99021 (1.5µM)	0	0	0	0	0%	0%					
	0	0	0	0	0%	0%					
	0	0	0	0	0%	0%					
	0	0	0	0	0%	0%					
	0.120	0.001	0	0	0%	0%					
	0.104	0.005	0	0	0%	0%					
	0	0	0	0	0%	0%					
	0	0	0	0	0%	0%					
CHIR99021 3µM	0.149	0.042	0	0	0%	0%					
	0.128	0.032	0	0	0%	0%					
	0	0	0	0	0%	0%					
	0	0	0	0	0%	0%					
	0.275	0.123	0.057	0.043	21%	16%	34%	17%	87	4	29
	0.161	0.017	0	0	0%	0%					
	0.179	0.066	0.046	0.011	27%	4%	29%	3%	98	37	28
	0.113	0.014	0	0	0%	0%					
	**0.392**	0.069	0.020	0.02	1%	3%	11%	13%	59	46	6
	0.281	0.182	**0.157**	0.0735	**60%**	10%	**70%**	9%	88	48	**62**
	0.194	0.042	0.114	0.0370	**58%**	11%	59%	8%	55	12	32
	0.310	0.085	0.119	0.0748	36%	14%	51%	15%	**126**	46	**65**
CHIR99021 4.5µM	0.289	0.012	0.031	0.001	9%	1%	9%	2%	166	12	15
	0.280	0.001	0.029	0.001	10%	0%	9%	3%	173	7	15
	0.114	0.007	0	0	0%	0%					
	0	0	0	0	0%	0%					
	0.250	0.088	0.163	0.034	67%	9%	**81%**	12%	**126**	24	**102**
	0.174	0.065	0.065	0.037	36%	7%	65%	14%	104	11	68
	0.269	0.046	**0.282**	0.006	**93%**	9%	**84%**	4%	75	11	64
	0.210	0.041	0.020	0.010	9%	4%	13%	6%	91	17	11
	0.291	0.100	0	0	0%	0%					
	**0.623**	0.081	0	0	0%	0%					
	0.191	0.006	0	0	0%	0%					
	0.256	0.027	0.105	0.032	41%	15%	26%	1%	83	7	22
CHIR99021 6µM	0.402	0.053	0.201	0.054	50%	10%	32%	5%	209	44	67
	0.466	0.040	0.246	0.041	53%	10%	33%	5%	202	39	68
	0.456	0.074	0.236	0.019	53%	11%	50%	5%	**232**	58	**116**
	0.203	0.080	0	0	0%	0%					
	0.295	0.019	0.013	0.013	1%	2%	3%	2%	117	9	3
	0.391	0.059	0.154	0.0747	38%	15%	24%	9%	172	14	41
	0.269	0.046	0.119	0.0286	44%	5%	35%	6%	104	16	36
	0.339	0.049	**0.265**	0.0075	**79%**	10%	**70%**	14%	122	18	85
	0.335	0.158	0	0	0%	0%					
	**0.981**	0.228	0	0	0%	0%					
	0.126	0.014	0	0	0%	0%					
	0.146	0.012	0	0	0%	0%					
CHIR99021 7.5µM	0.253	0.072	0	0	0%	0%					
	0.276	0.048	0	0	0%	0%					
	0.430	0.017	0.221	0.019	51%	6%	23%	4%	149	13	34
	0.534	0.086	0.137	0.070	25%	11%	27%	16%	**286**	58	**76**
	0.448	0.044	0	0	0%	0%					
	0.510	0.102	0.024	0.024	5%	10%	3%	4%	152	41	4
	0.207	0.011	0	0	0%	0%					
	0.405	0.022	**0.260**	0.002	**64%**	3%	**29%**	5%	59	13	17
	0.113	0.009	0	0	0%	0%					
	**0.913**	0.135	0	0	0%	0%					
	0	0	0	0	0%	0%					
	0.104	0.006	0	0	0%	0%					
CHIR99021 9µM	0.301	0.055	0	0	0%	0%					
	0.274	0.045	0	0	0%	0%					
	0.315	0.088	0.037	0.050	10%	12%	6%	3%	211	68	13
	0.575	0.072	**0.223**	0.038	39%	6%	**30%**	7%	**244**	50	**73**
	0.550	0.049	0	0	0%	0%					
	0.232	0.144	0	0	0%	0%					
	0.285	0.088	0	0	0%	0%					
	0.419	0.079	0.183	0.040	**44%**	9%	17%	3%	116	35	19
	0.111	0.003	0	0	0%	0%					
	**0.806**	0.206	0	0	0%	0%					
	0	0	0	0	0%	0%					
	0	0	0	0	0%	0%					

TABLE S2

hPSCS analyses and cardiac differentiation results with CHIR99021

Cell lines		FR201	FR202	BM1 (1)	BM1 (2)	X.13	IMR-90 (1)	4Skin	Donor 5 (1)	Donor 5 (2)	CB6	DF6 (1)	DF6 (2)	HES-3 (M1)	HES-3 (M2)	H7	FR202 (I)	H1 (I)	IMR-90 (2)
Passage Number		P10-4	P10-4	p11	p13	p11-8	p5-32-8-7	p12-13(F)-10	batch 1 p36	batch 2 p35	p18	batch 1 p28	batch 2 p29	p2-14-7	p2-14-7	p17	p10-5-7	p14-19-9	p5-32-8-8
**Day 0**																			
Pluripotency	Oct4a	83%	80%	90%	90%	76%	77%	88%	90%	50%	61%	48%	38%	98%	98%	87%	93%	97%	88%
	Nanog	83%	83%	90%	88%	62%	76%	89%	81%	36%	55%	48%	30%	97%	98%	77%	92%	96%	89%
Cell Confluency		75%	85%	95%	95%	98%	86%	83%	88%	n.a.	n.a.	76%	80%	96%	62%	85%	70%	70%	70%
Metabolism	sub-G1	2%	2%	9%	26%	23%	9%	31%	4%	51%	22%	54%	27%	4%	5%	2%	1%	2%	2%
	G1	52%	56%	42%	38%	48%	64%	38%	67%	42%	43%	25%	47%	55%	47%	66%	41%	38%	52%
	S	17%	20%	20%	19%	16%	17%	16%	14%	11%	16%	12%	13%	26%	27%	15%	23%	30%	23%
	G2/M	29%	22%	29%	15%	12%	10%	14%	16%	8%	19%	7%	12%	15%	21%	14%	33%	29%	21%
Cell Cycle (S+G2/M)		**47%**	**42%**	**49%**	**34%**	**28%**	**27%**	**30%**	**30%**	**19%**	**34%**	**19%**	**25%**	**41%**	**47%**	**29%**	**55%**	**59%**	**44%**
DC Protein Assay Protein Mass (ug/ml)		6.844203	6.612319	2.914855	7.014493	17.02174	12.9837	9.338768	8.387681	8.282609	7.373188	8.815217	9.865942	13.97826	4.17029	13.12681	2.83	4.03	2.01
Protein expression (Chemilunisecence)																			
	TCF7L1	144665.9	181283	326034	160757.4	128404	228057.6	165814.1	179474	n.a.	180852	199080	n.a.	222527	341141	179379.3	202406.3	n.a.	429887
	TCF7L2	12049.75	9486	37742	33508.27	28838.28	40234.62	18341.51	34508	n.a.	23451	31901.03	n.a.	44533.44	87471	34039.35	25050.81	n.a.	69996.1
	b-catenin	124188.7	n.a.	n.a.	107816.7	90726.97	131823.5	176241.9	n.a.	n.a.	139845.4	133235.6	n.a.	121072.9	n.a.	130641.2	120201	n.a.	119211
**Day 1**	CHIR99021																		
Protein DC Assay (µg/ml)	0µM	n.a.	n.a.	n.a.	n.a.	5.966305	n.a.	n.a.	n.a.	n.a.	n.a.	n.a.	n.a.	10.72105	n.a.	n.a.	n.a.	n.a.	n.a.
	4µM	n.a.	n.a.	n.a.	n.a.	7.515218	8.893478	n.a.	6.376631	<0.2	n.a.	n.a.	1.136232	19.77264	n.a.	7.699275	n.a.	n.a.	n.a.
	6µM	1.576993	2.009964	6.302174	4.113768	6.567391	8.68913	5.726087	5.563044	<0.2	7.588406	3.303261	2.977536	20.19203	n.a.	8.099638	4.748458	5.687044	4.83
	8µM	12.47826	8.605435	6.734783	5.602989	7.717391	9.089131	8.408696	5.929891	<0.2	5.267391	2.080435	1.780435	n.a.	n.a.	8.307971	4.730791	5.982427	4.42
	10µM	n.a.	n.a.	5.254348	3.06413	6.258696	8.432609	5.584239	4.213043	n.a.	0.76558	1.926087	0.513043	n.a.	n.a.	10.75815	3.918116	7.867639	4.04
	12µM	n.a.	n.a.	1.280072	4.0125	n.a.	8.43913	5.205978	n.a.	n.a.	n.a.	0.563768	n.a.	14.47924	n.a.	7.326087	3.228454	n.a.	4.42
Peak Protein Mass Change (%) 24h		**82%**	**30%**	**131%**	**-20%**	**-55%**	**-30%**	**-10%**	**-24%**	**-100%**	**3%**	**-63%**	**-70%**	**44%**		**-18%**	**68%**	**95%**	**140%**
**Day 14**																			
Peak Nkx2-5 expression (%)		13.9	4.95	44.7	48.7	41.8	31	29.2	14.4	cell death	6.41	13.4	2.81	95.5	92.8	59.3	47	73	83
CHIR99021 + IWR-1																			
Peak Nkx2-5 expression (%)		21.4	8.22	74.3	75.8	57.9	32.7	72.5	25.1	cell death	2.15	42.9	cell death	95	n.a.	69.3	69	n.a.	n.a.
Troponin T (%)	4µM	n.a.	n.a.	n.a.	n.a.	1.51	0.661	n.a.	3.46	n.a.	2.1	n.a.	n.a.	n.a.	n.a.	5.23	n.a.	n.a.	n.a.
	6µM	2.68	1.79	11.1	7.29	3.19	2.08	4.02	18.2	n.a.	0.757	14.1	3.52	9.37	11.1	10.4	0.6	2.6	54.1
	8µM	4.08	5.26	48.7	46.2	48.7	2.9	21.2	7.2	n.a.	1.55	10.7	n.a.	39.4	22.5	21.2	41.5	60.3	83.1
	10µM	29.3	17.7	26.9	5.88	1.53	9.54	23.2	1.02	n.a.	7.46	1.23	n.a.	42.5	21.1	42.2	56.6	73.7	54.2
	12µM	*	*	0.81	0.955	n.a.	2.06	10.3	n.a.	n.a.	n.a.	n.a.	n.a.	87.1	86.1	44.2	60.5	n.a.	11.2
Peak Troponin T expression (%)		**29.3**		**48.7**	**46.2**	**48.7**	**9.54**	**23.2**	**18.2**	**0**	**7.46**	**14.1**	**3.52**	**87.1**	**86.1**	**44.2**	**60.5**	**73.7**	**83.1**
CHIR99021 + IWR-1																			
Peak Troponin T expression (%)		39.3	27.5	75.6	71.3	60	17.8	79.1	31.9	cell death	6.36	41.1	cell death	88	n.a.	51	91	n.a.	n.a.

*outlier! Missing date from 12µM experiment; data not included in day 14 calculations

TABLE S2

hPSCS analyses and cardiac differentiation results with CHIR99021

Charts from Table S2

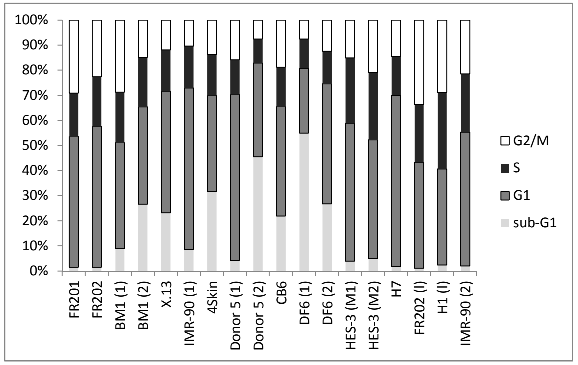

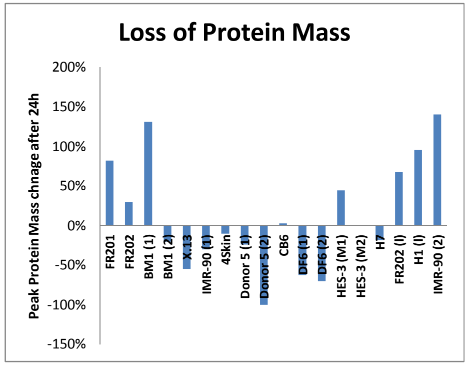

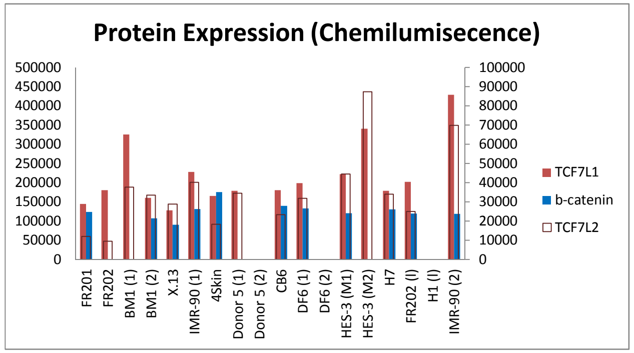

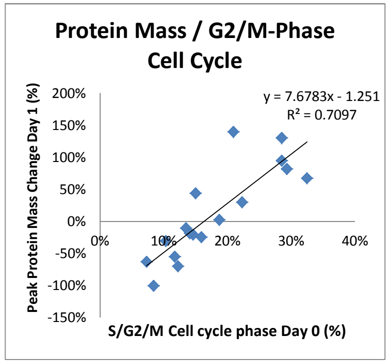

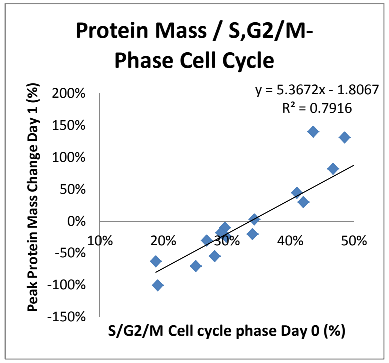

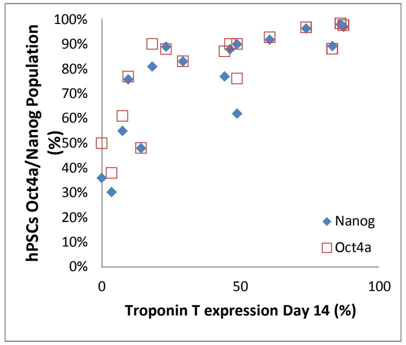

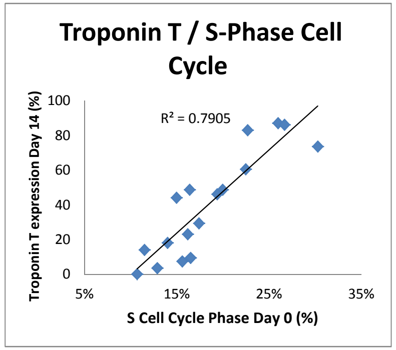

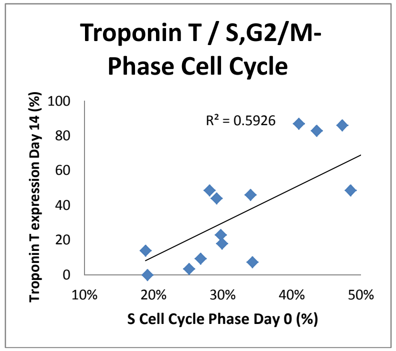

TABLE S3

All Data for CST lyses with a Protein weight of 0.5mg/ml, 12-130KD Protein Simple Kit

Antibody	Company	#	Clone	Iso-tope	2nd Ab	Ex-pected kD	Protein Simple kD	Back-ground	WB IPS/ES	WB Differen-tiation	Cardio-myocytes Flow cytometry	IF
**α-actinin-4**	Santa Cruz	390180	(B-11)	IgG2a	m	105	91-97		1:50	1:50		
**b-actin**	Cell Signaling	4967	pAb	-	r	45	45		1:500	1:500		
**AKT**	Cell Signaling	9272	pAb	-	r	60	62		1:50	1:50		
**Phospho-AKT (ser473)**	Cell Signaling	4060	(D9E)	IgG	r	60	64		1:50	1:50		
**Axin 1**	Cell Signaling	2087	(C76H11)	IgG	r	110	112-117		1:75	1:75		
**b-catenin (IF)**	Cell Signaling	2677	(L54E2)	IgG1	m	-	-	-	-	-		1:200
**b-catenin**	Cell Signaling	2698	(L87A12)	IgG1	m	92	79-82		1:500	1:500		
**b-catenin**	Cell Signaling	8480	(D10A8)	IgG1	r	92	80		1:1500	1:1500		
**Active-b-catenin**	Cell Signaling	8814	(D13A1)	IgG	r	92	82-87		1:1500	1:1500		1:200
**Phospho-b-catenin (ser675)**	Cell Signaling	5176	(D2F1)	IgG	r	92	82-85		1:250	1:250		
**Phospho-b-catenin (ser552)**	Cell Signaling	9566	pAb	-	r	92	81-90		1:75	1:75		
**Ph-b-catenin (ser33/37/thr41)**	Cell Signaling	9561	pAb	-	r	92	90-96		1:20	1:20		
**Phospho-b-catenin (ser45)**	Cell Signaling	9564	pAb	-	r	92	85-89		1:50	1:50		
**b-tubulin**	Cell Signaling	2146	pAb	-	r	55	58		1:400	1:400		
**bFGF**	Cell Signaling	3196	(19A9)	IgG	r	19	26-29		1:25	-		
**Phospho-cdc2 (Tyr15)**	Cell Signaling	4539	(10A11)	IgG	r	34	40		1:25	-		
**Phospho-Chk1 (Ser345)**	Cell Signaling	2348	(133D3)	IgG	r	56	68		1:25	-		
**Cyclin A1/2**	Santa Cruz	751	(H-432)	IgG	r	54	50/59		1:100	-		
**Cyclin B1**	Cell Signaling	4135	(v152)	IgG1	m	55	64		1:100	-		
**Cyclin D1**	Cell Signaling	2978	(92G2)	IgG	r	36	40		1:25	1:25		
**Phospho-Cyclin D1 (Thr286)**	Cell Signaling	3300	(D29B3)	IgG	r	36	40		1:25	1:25		
**Cyclin E**	Santa Cruz	25303	(E-4)	IgG1	m	53	59		1:100	-		
**Dishevelled 2**	Cell Signaling	3224	(30D2)	IgG	r	90-95	105		1:100	1:100		
**DKK-1**	LSBio	ls-b8698	EPR4759		r	36	38-41		1:250	1:250		
**FoxA2/HNF3b**	Cell Signaling	8186	(D56D6)	IgG	r	50	58		1:25	1:100		
**FoxO1**	Cell Signaling	2880	C29H4	IgG	r	78-82	85		1:100	1:100		
**FoxO3a**	Cell Signaling	12829	(D19A7)	IgG	r	82-97	90		1:100	1:100		
**GAPDH**	Santa Cruz	365062	(G-9)	IgG1	m	37	39-41		1:40000	1:40000		
**GAPDH**	Cell Signaling	2118	(14C10)	IgG	r	37	39-41		1:4000	1:4000		
**GATA4**	Cell Signaling	14353	pAB	-	r	55	55-59		1:25	1:25		
**GATA6**	Cell Signaling	5851	(D61E4)	IgG	r	55	58-62		1:25	1:25		
**GSK-3b**	Cell Signaling	12456	(D5C5Z)	IgG	r	46	55		1:1000	1:1000		
**HDAC2**	Cell Signaling	5113	(3F3)	IgG1	m	60	60		1:200	1:200		
**Phospho-GSK-3b (ser9)**	Cell Signaling	9323	(5B3)	IgG	r	46	55		1:50	1:50		
**Phospho-GSK-3b (thr380)**	Cell Signaling	3548	pAb	-	r	46	55		1:25	-		
**HSP60**	Cell Signaling	12165	(D6F1)	IgG	r	60	55-59		1:500	1:500		
**HSP70**	Santa Cruz	32239	(3A3)	IgG	m	70	77		1:1500	1:1500		
**LaminA/C**	Cell Signaling	4777	(4C11)	IgG2a	m	74,63	75		1:25	-		
**LEF1 (TCF7L3)**	Cell Signaling	2230	(C12A5)	IgG	r	25, 58	60		1:25	1:50		
**Phospho-LRP6 (ser1490)**	Cell Signaling	2568	pAb	-	r	180/210	200-210		1:25	1:25		
**LRP6**	Cell Signaling	2560	(C5C7)	IgG	r	180/210	200-210		1:200	1:200		
**MIXL1**	Santa Cruz	390976	(A-8)	IgG	m	25	29		1:25	1:25		
**MEF2c**	Cell Signaling	5030	(D80C1)	IgG	r	50-60	58-62		1:50	1:50		
**MESDC2**	Cell Signaling	2763	pAb	-	r	25/26	36		1:25	1:25		
**NANOG**	Cell Signaling	4903	(D73G4)	IgG	r	42	56		1:50	1:50		
**NKX2.5**	Cell Signaling	8792	(E1Y8H)	IgG	r	30-42	47-49		1:50	1:50	1:300	
**p53**	Santa Cruz	126	(DO-1)	IgG2a	m	53	57		1:20	-		
**Phospho-p53 (Ser15)**	Cell Signaling	9286	(16G8)	IgG1	m	53	57		1:10	-		
**PDGFRa**	Cell Signaling	5241	(D13C6)	IgG	r	-	-	-	-	-	1:200	
**Oct4a**	Cell Signaling	2840	(C30A3)	IgG	r	45	47-49		1:50	1:50		
**SMAD1**	Cell Signaling	6944	(D59D7)	IgG	r	62	64		1:100	1:100		
**Phospho-SMAD1 (ser206)**	Cell Signaling	5753	(D40B7)	IgG	r	62	64		1:25	1:25		
**Slug**	Cell Signaling	9585	(C19G7)	IgG	r	30	44		1:50	1:50		
**Snail**	Cell Signaling	3879	(C15D3)	IgG	r	29	42		1:50	1:50		
**SOX2**	Cell Signaling	3579	D6D9	IgG	r	-	-	-	-	-	1:400	
**SOX17**	Cell Signaling	13863	pAb	-	r	55	54/65		1:25	1:75		
**T-Bra**	Santa Cruz	374321	(A-4)	IgG2b	m	49	52		1:100	1:100		
**T-Bra**	R&D	IC2085G	pAb	IgG	g	49	52	-	1:10	1:10	1:50	
**T-Bra**	Cell Signaling	81694	(D2Z3J)	IgG	r	49	52		1:400	1:400	1:100	
**TCF1 (TCF7)**	Cell Signaling	2203	(C63D9)	IgG	r	48/50	54/66/90		1:25	1:50		1:200
**TCF3 (TCF7L1)**	Cell Signaling	2883	(D15G11)	IgG	r	78	95		1:100	1:100		
**TCF4 (TCF7L2)**	Cell Signaling	2569	(C48H11)	IgG	r	58, 79	63/91		1:50	1:50		
**TRA-1-60**	Cell Signaling	4746	-	IgM	m	-	-	-	-	-	1:300	
**Troponin T**	ThermoScientific	MA5-12960	(13-11)	IgG	m	39	47-48		1:50	1:50	1:400	
**Phospho-Wee1 (Ser642)**	Cell Signaling	4910	(D47G5)	IgG	r	95	110		1:25	-		
**Wnt3a**	Cell Signaling	2721	(C64F2)	IgG	r	42	44		1:25	1:25		
**Mitotic Spindle Apparatus**	R&D	test sample		IgG	m	-	-	-	-	-	-	1:50
